# ISGylation of DRP1 closely balances other post-translational modifications to mediate mitochondrial fission

**DOI:** 10.1038/s41419-024-06543-7

**Published:** 2024-03-02

**Authors:** Palamou Das, Oishee Chakrabarti

**Affiliations:** 1 Biophysics & Structural Genomics Division, Saha Institute of Nuclear Physics, 1/AF Bidhannagar, Kolkata, 700064 India; 2 Homi Bhabha National Institute, Mumbai, India

**Keywords:** Ubiquitylation, Protein quality control

## Abstract

Dynamin related protein 1 (DRP1), a pivotal mitochondrial fission protein, is post-translationally modified by multiple mechanisms. Here we identify a new post-translational modification of DRP1 by the ubiquitin-like protein, interferon-stimulated gene 15 (ISG15). DRP1 ISGylation is mediated by ISG15 E3 ligase, HERC5; this promotes mitochondrial fission. DeISGylation of DRP1 however leads to hyperfusion. Heterologous expression of SARS-CoV2 PLpro, a deISGylating enzyme, results in similar mitochondrial filamentation, significant decrease in total DRP1 protein levels and efflux of mtDNA. We report that deISGylated DRP1 gets ubiquitylated and degraded by TRIM25, instead of PARKIN and MITOL. While the cytosolic pool of DRP1 is primarily ISGylated, both mitochondrial and cytosolic fractions may be ubiquitylated. It is known that phosphorylation of DRP1 at S616 residue regulates its mitochondrial localisation; we show that ISGylation of phospho-DRP1 (S616) renders fission competence at mitochondria. This is significant because DRP1 ISGylation affects its functionality and mitochondrial dynamics in Alzheimer’s disease pathophysiology.

## Introduction

Balanced mitochondrial dynamics is one of the crucial factors for maintaining cellular homeostasis. Mitochondrial fusion allows even distribution of membrane as well as matrix components, provides connectivity (electrical, mechanical as well as energetic) across the network. On the other hand, fission helps distribution of mitochondria to daughter cells, maintain quality control and organellar turnover [1]. Mitochondrial fusion is mediated by the outer membrane components [mitofusins 1 and 2 (MFN1, MFN2)] and inner membrane protein [optic atrophy 1 (OPA1)]. Mutations have been identified in the fusion proteins MFN2 and OPA1 causing various diseases. Mutation in MFN2 have been detected in neuropathies, like Charcot–Marie–Tooth type 2 A (CMT2A) and other type of Hereditary Motor and Sensory Neuropathies (HMSNs), though the exact mechanism of protein deregulation is being understood only recently [2, 3]. Alterations in MFN2 activity is also implicated in cardiac dysfunction and Type 2 diabetes [4]. Mutations in OPA1 are associated with Autosomal Dominant Optic Atrophy (ADOA type 1), a juvenile a neuroophthalmic disorder [5, 6]. Some patient mutations have also been identified in Dynamin-related protein 1 (DRP1) [7], though a phenotype to functional correlation remains largely speculative. Needless to state that the fission associated genes are less studied than the ones participating in mitochondrial fusion.

DRP1, along with its various adaptor proteins FIS1 (mitochondrial fission protein 1), MFF (mitochondrial fission factor), MiD49 and MiD51 (mitochondrial dynamics proteins of 49 and 51 KDa) are suggested to participate in the mitochondrial fission process [8]. Binding with adaptor proteins potentiates recruitment of DRP1, oligomerisation of its monomers at the place of scission and helps execute the final fission of a mitochondrion into two [9]. While it is well understood that DRP1 is one of the crucial factors responsible for mitochondrial fission, its regulation in maintaining cellular homeostasis is yet to be fully explored. DRP1 function is also known to be modulated by a number of post translational modifications, like phosphorylation, ubiquitylation and SUMOylation [10, 11]. It is hence plausible to hypothesize that maintenance of an intricate balance between these modifications would affect DRP1 stability, function and eventually control mitochondrial dynamics.

Interferon stimulated gene 15 (ISG15), one of the notable players of IFN-I response [12, 13] belongs to the Ubiquitin (Ub) family of post-translational modifiers [14]. The ISG15 precursor is a 17KDa protein, consisting of two linked Ub like domains; this is further processed proteolytically to form a 15 KDa mature protein [15]. Covalent conjugation of ISG15 to its target proteins is referred as ISGylation [16]. Unlike with Ub-mediated modifications, our understanding of the fate of ISGylated and deISGylated target proteins is still premature.

IFN-stimulated genes (ISGs) are known for their antiviral properties [17]. One such molecular player, ISG15 expression is stimulated upon viral infections, with or without the activation of elements downstream of STING [18]. Further ISG15-modification of viral and host proteins can alter viral replication and host immune response; though the actual molecular mechanisms are still largely unknown [14, 19, 20]. Recent literature suggests that the CoV2 PLpro preferentially cleaves off ISG15 moieties from post-translationally modified proteins and hence acts as a deISGylase [21, 22]. It is plausible that by deISGylating host proteins, CoV2 could be deregulating the host immune response.

Mitochondrial DNA (mtDNA), a 17 kb double stranded circular DNA present in the mitochondrial matrix, when localised outside the organelle, is known to activate IFN-I response [23]. Extra-mitochondrial mtDNA can be released by multiple mechanisms under various conditions. mtDNA efflux occurs during apoptosis through BAX/BAK macropores [24]. mtDNA may be released through pores formed by the voltage dependent anion channel (VDAC) oligomers; this is specifically reported as factor a contributing to the disease severity in systemic lupus erythematosus model system [25]. Mitochondrial outer membrane permeabilisation can also lead to mtDNA release during apotosis [26]. Under all these circumstances, cytosolic mtDNA activates the cGAS-STING (cyclic GMP-AMP synthase - stimulator of interferon genes) pathway and in turn induces IFN-I response. Type I IFN response genes are established players of frontline defence against various viral infections [27]. Presence of extra-mitochondrial mtDNA and activation of IFN-I response elements downstream of the cGAS-STING pathway is detected during infections by viruses, like Influenza A, Herpes simplex and Dengue [28–30] Further, this type of inflammatory response due to efflux of mtDNA is also reported in neurological disorders and cancers [31, 32]. More recently, it is suggested that CoV2 infection can stimulate the cGAS-STING pathway [33]. However, the mechanism by which CoV2 activates this pathway remains unknown. A subset of patients infected with CoV2 has shown marked upregulation of pro-inflammatory responses, commonly referred as “cytokine storm”; this positively correlates with increased fatality. While IFN-I is believed to protect the host against virus, it is also speculated to cause deleterious effects [34]. However, the source of IFN-I in the lungs of infected patients remains ill-defined.

Here we show that for the first time a new post-translational modification, ISGylation of DRP1. Its levels are maintained in cells by a critical balance between the post-translationally modified forms. The cytosolic pool is primarily ISGylated, while ubiquitylated forms are present on both the cytosolic and mitochondrial fractions. DeISGylating enzyme, CoV2 PLpro, disturbs this homeostasis, skews the balance towards ubiquitylation of DRP1 and its eventual degradation. In the presence of CoV2 PLpro, mtDNA is released from mitochondria. This is accompanied by the activation of IFN-I response genes, including ISG15. mtDNA efflux occurs from hyperfused filamentous mitochondria, that are morphologically altered due to decrease in the levels of DRP1. S616 phophorylated form of DRP1 requires ISGylation for its fission potential at the mitochondria. Our analysis posits the role of various post-translational modifications of DRP1 as one of the crucial regulators governing this protein’s differential cellular distribution and function. DRP1 ISGylation is physiologically relevant in Alzheimer’s disease pathogenesis.

## Results

### ISGylation of DRP1 via HERC5 regulates mitochondrial dynamics

**Fig. 1:**
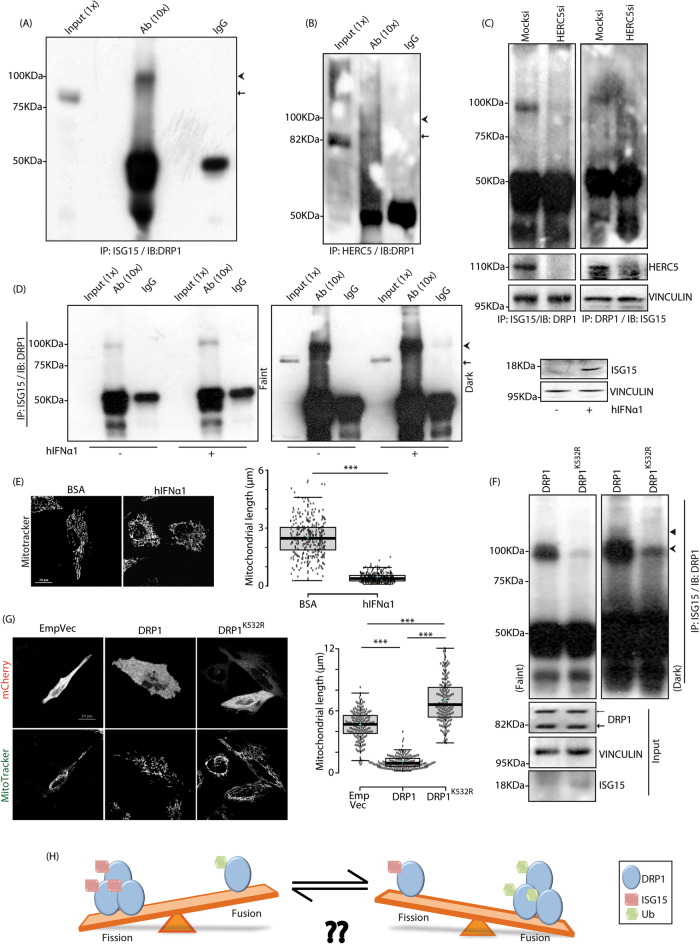
ISGylation of DRP1 by HERC5 and effect on mitochondrial dynamics. **A** A549 cell lysates were immunoprecipitated (IP) with anti-ISG15 antibody. Western blot (IB) analysis of extract with anti-DRP1 antibody shows co-immunoprecipitation (IP) of ISG15-modified DRP1. Note a shift in band size detected between input and immunoprecipitated samples. The proportion of lysate loaded as input and used for immunoprecipitation is denoted in brackets by ‘X’.  endogenous DRP1,  ISGylated endogenous DRP1. **B** Cell lysates co-immunoprecipitated to verify interaction between HERC5 with DRP1. The proportion of lysate loaded as input and used for immunoprecipitation is denoted in brackets by ‘X’.  endogenous DRP1,  ISGylated endogenous DRP1. **C** Mock or HERC5 siRNAs-treated cell lysates were immunoprecipitated (IP) with anti-ISG15 antibody (left panel). Note Western blot (IB) analysis with anti-DRP1 antibody shows decrease in ISGylated-DRP1 in HERC5-depleted samples. Reverse co-IP on the right corroborates the same. HERC5 and VINCULIN confirm knockdown and loading efficiencies. **D** A549 cells treated with 10 ng/ml of human IFNα1 (hIFNα1) for 48 h were lyzed and checked for ISGylation of DRP1 by co-IP between ISG15 and DRP1. Note that treatment with hIFNα1 induces enhanced ISGylation of DRP1. Similarly hIFNα1 treatment results in higher ISG15 expression, VINCULIN served as loading control. The proportion of lysates and immunoprecipitates denoted by ‘X’ in brackets.  endogenous DRP1,  ISGylated endogenous DRP1. **E** Cells treated with the hIFNα1 as described in panel D and treated with MitoTracker Red FM were imaged under live-cell conditions. Scale bar, 20μm. Box plots showing quantification of mitochondrial length for the experiment. ~150 cells from 3 independent experiments were analysed. The central line and the plus (+) symbol in each box show the median and mean value, respectively. ***p ≤ 0.001 using unpaired 2-tailed Student’s t-test. **F** A549 cells were transfected with indicated mCherry-tagged constructs and co-immunoprecipitated against ISG15 and DRP1. Note that K532R mutation in DRP1 compromises its ISGylation; ISGylated DRP1 band being more prominent in the dark exposure. ISG15 and VINCULIN served as loading control.  ISGylated endogenous DRP1, ◄ ISG15-modified mCherry-tagged DRP1,  endogenous DRP1, ← mCherry-tagged DRP1. **G** Cells were transfected with indicated mCherry-tagged constructs and treated with MitoTracker Green FM were imaged under live-cell conditions. Scale bar, 20μm. Box plots showing quantification of mitochondrial length for the experiment. ~200 cells from 3 independent experiments were analysed. The central line and the plus (+) symbol in each box show the median and mean value, respectively. ***p ≤ 0.001 using unpaired 2-tailed Student’s t-test. **H** Experimental hypothesis regarding differential effects of post-translational modifications (ISGylation and ubiquitylation) of DRP1 on mitochondrial dynamics.

Various post-translational modifications like, ubiquitylation, phosphorylation and SUMOylation are known for mitochondrial fission protein DRP1 [10]. We analysed if DRP1 could be modified by the Ub-like protein, ISG15. Co-immunoprecipitation analyses between DRP1 and ISG15 indicated interaction between the two proteins in A549, lung adenocarcinoma-derived hypotriploid alveolar basal epithelial cells; a band corresponding to mono-ISGylated DRP1 was evident (Figs. 1A, S1A). HERC5 is one of the major ISG15 ligating enzymes in cells [35]. Co-immunoprecipitation results suggested interaction between HERC5 and DRP1 (Figs. 1B, S1B). Further, knocking down HERC5 severely compromised ISGylation of DRP1 (Fig. 1C). Treating cells with hIFNα1 resulted in elevated ISG15 protein levels and a simultaneous increase in ISGylated DRP1 (Fig. 1D). To further validate whether the ISGylation profile of DRP1 has any effect upon mitochondrial dynamics, imaging studies showed that mitochondrial length and volume diminished significantly in the presence of hIFNα1 (Figs. 1E, S1C) with a corresponding increase in the number of total mitochondria per cell. Similarly, ISG15 overexpression in cells, led to decreased mitochondrial length (Figures S1D, S1E). Lysine to arginine mutation in DRP1 (DRP1^K532R^) severely compromised its ISGylation (Fig. 1F); in presence of the mutant, stimulation with hIFNα1 partially rescued DRP1 ISGylation (Figure S1F). Detection of ISG15-modified DRP1^K532R^ upon hIFNα1 stimulation could be because of the presence of endogenous DRP1 or due to ISGylation at another lysine residue. Compromised DRP1 ISGylation in the presence of DRP1^K532R^ phenotypically resulted in significant increase in mitochondrial length and volume (Figs. 1G, S1G), with a significant decrease in the number of total mitochondria per cell in presence of this DRP1 variant. Further, the knockdown of ISG15 led to increased mitochondrial legth and volume, and reduced total number of mitochondria per cell (Figures S1H, S1I). This led us to hypothesize that the mitochondrial fission-fusion balance could in turn be controlled by an interplay between post-translational modifications of DRP1 (Fig. 1H).

### CoV-2 PLpro affects mitochondrial dynamics

**Fig. 2:**
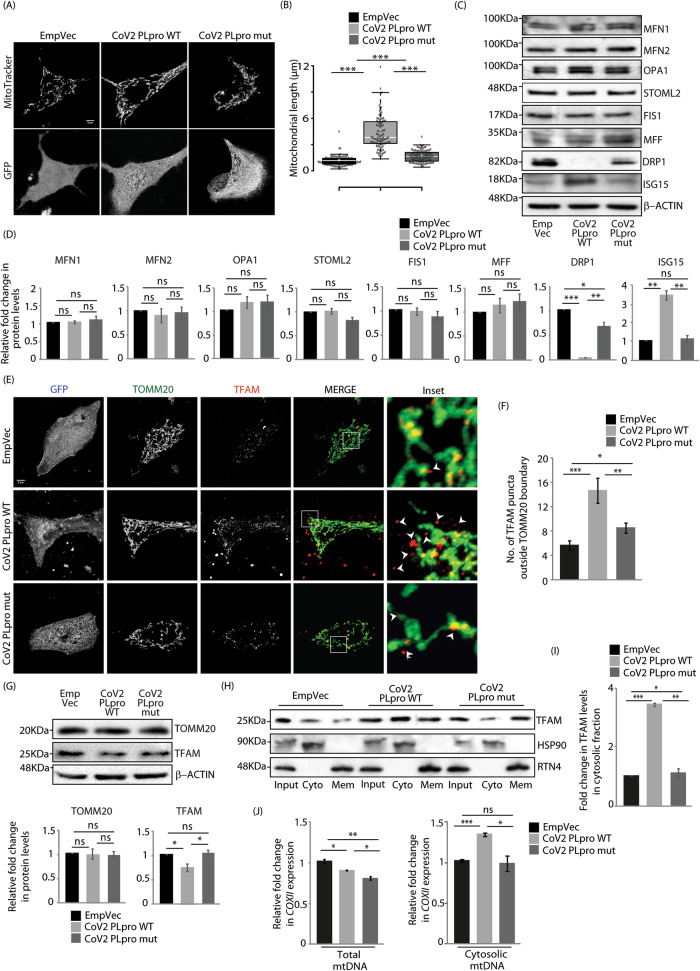
Reduced DRP1 levels and mtDNA release in presence of CoV-2 PLpro. **A** Cells transfected with the indicated GFP-tagged constructs and treated with MitoTracker Red FM were imaged under live-cell conditions. Scale bar, 5μm. **B** Box plot showing quantification of mitochondrial length for the experiment described in A. ~90 cells from 3 independent experiments were analysed. The central line and the plus (+) symbol in each box show the median and mean value, respectively. ***p ≤ 0.001 using unpaired 2-tailed Student’s t-test. **C** A549 cells transiently transfected with the indicated GFP-tagged constructs were immunoblotted against the indicated antibodies. **D** Graphs plot changes in expression of proteins analysed in panel C. Data represent the mean ± SEM of three independent experiments. ns, not significant (*p*>0.1), *p ≤ 0.05, **p ≤ 0.01, ***p ≤ 0.001 using unpaired 2-tailed Student’s t-test. **E** Cells were transfected with indicated GFP-tagged constructs and immunostained with antibodies against TOMM20 and TFAM. Z-stacks (0.15μm slices) were taken. Representative images show 3-D projections. Enlarged views of the areas within the white boxes shown (insets). White arrowheads mark TFAM puncta outside TOMM20 boundary. Scale bar, 5μm. **F** Graph represents data from ∼175 cells from 3 independent experiments. *p ≤ 0.05, **p ≤ 0.01, ***p ≤ 0.001 using unpaired 2-tailed Student’s t-test. Error bars,±SEM. **G** A549 cells transfected similarly as in panel E and were immunoblotted against TOMM20 and TFAM antibodies, β-ACTIN was used as loading control. Graphs plot show changes in expression of proteins analysed. Data represent the mean ± SEM of 3 independent experiments. ns, not significant (p > 0.6), *p ≤ 0.05 using unpaired 2-tailed Student’s t-test. **H** Cytosolic and membrane fractions obtained from digitonin permeabilised A549 cells were immunoblotted with TFAM antibody; inputs indicate total protein in cell lysates. HSP90 and RTN4 served as controls for cytosolic and membrane fractions, respectively. **I** Graph with data from panel H shows results of 3 independent experiments. *p ≤ 0.05, **p ≤ 0.01, ***p ≤ 0.001 using unpaired 2-tailed Student’s t-test. Error bars, ±SEM. **J** DNA isolated from whole cell and cytosolic extracts was subjected to SYBR Green-based qPCR to quantify nuclear (*GAPDH*) and mitochondrial (*COXII*) DNA using specific primers. Plots show abundance of total (left) and cytosolic (right) cellular mtDNA. ns, not significant (*p* = 0.7), *p ≤ 0.05, **p ≤ 0.01, ***p ≤ 0.001 using unpaired 2-tailed Student’s t-test. Error bars, ±SEM.

CoV2 PLpro, primarily involved in viral replication and transcription [36], preferentially cleaves off ISG15 modifications from substrate proteins [21]. Using this as a major deISGylating tool, cells were transfected with GFP-tagged wild-type CoV2 PLpro (CoV2 PLpro WT) or its catalytically inactive C111S mutant form (CoV2 PLpro mut). Experiments show an increase in average mitochondrial length and volume, along with a decrease in their numbers in presence of the wild-type protease (Figs. 2A, B, S2A). Total ATP produced was significantly less in cells with CoV2 PLpro WT; we also detected compromised mitochondrial contribution in the metabolic profile of these cells (Figures S2B, S2C). The alteration in mitochondrial morphology in the presence of the wild type protease was corroborated in human hepatoma (HepG2) cells, indicating a cell line-independent phenomenon (Figures S2D, S2E). Further, cell lysates were analysed for the expression profile of proteins involved in the regulation of mitochondrial dynamics (Fig. 2C, D). Most proteins implicated in mitochondrial fission-fusion dynamics (MFN1, MFN2, OPA1, STOML2, FIS1 and MFF) remained unaltered across samples. However, a significant decrease in DRP1 levels were observed in the presence of CoV2 PLpro WT, compared with the mutant or the control. Significant increase in monomeric ISG15 protein levels was also detected in the CoV2 PLpro WT samples. As expected, MFN2 ISGylation [37] is compromised in samples with wild-type protease (Figure S2F). Literature suggests that increased ISG15 levels correlate with the presence of cytosolic mtDNA [25]. Investigations showed an increase in the levels of cytosolic mtDNA in the presence of CoV2 PLpro WT when compared with its mutant. This was validated in multiple ways. First, A549 cells transfected with GFP-tagged CoV2 PLpro WT or its mutant were immunostained against the outer mitochondrial membrane (OMM) marker TOMM20 and TFAM (mitochondrial transcription factor A), and imaged (Figs. 2E, F, S2G). TFAM, being an mtDNA binding protein, is primarily detected inside TOMM20-positive mitochondria in the controls as well as the mutant expressing cells. In the presence of CoV2 PLpro WT, however, a significant increase in TFAM positive puncta was detected outside TOMM20 boundary. Compromised cristae architecture and presence of Pico Green positive puncta outside LIVE ORANGE mito supported mtDNA release from mitochondria (Figure S2H). Similar results were corroborated in HepG2 cells (Figures S3A, S3B). While the TOMM20 protein levels remains similar across samples, a decrease in TFAM levels was observed in CoV2 PLpro WT samples (Fig. 2G). Secondly, analyses of fractions generated from digitonin-mediated semi-permeabilisation of A549 cells indicated increase in cytosolic TFAM levels in presence of CoV2 PLpro WT, when compared with the mutant (Fig. 2H, I). A corresponding decrease in TFAM levels in the membrane fractions (containing mitochondria) was also detected in cells expressing the wild type protease (Figures S3C, S3D). Similar results were seen in semi-permeabilised HepG2 cells (Figures S3E, S3F) as well as in mitochondrial fractions obtained from A549 cells (Figures S3G, S3H). Thirdly, quantitative PCR (qPCR) was performed on cytosolic DNA and compared with the total cellular DNA. Results showed a significant increase in *COXII* (cytochrome c oxidase subunit II) and *mtND1* (mitochondrially encoded NADH dehydrogenase 1) levels in cytosolic DNA in the presence of CoV2 PLpro WT, compared with the mutant or the control (Figs. 2J, S3I). The presence of this nsp3 protease (wild type or mutant) also led to a decrease in the total mtDNA content. All these results taken together suggested an increase in the levels of extra-mitochondrial mtDNA in the cytosol when wild type CoV2 PLpro was expressed triggering IFN-I activation and signalling in response to the presence of cytosolic DNA (Figure S3J) [24, 26]. While cleaving off ISG15 modifications from host proteins can render them non-functional and help the virus evade immune response, activation of IFN-I downstream effectors in the presence of CoV2 PLpro suggests the presence of a very complicated and intricate balance of the virus with the host – much of which remains to be explored.

### mtDNA release from hyperfused mitochondria

**Fig. 3:**
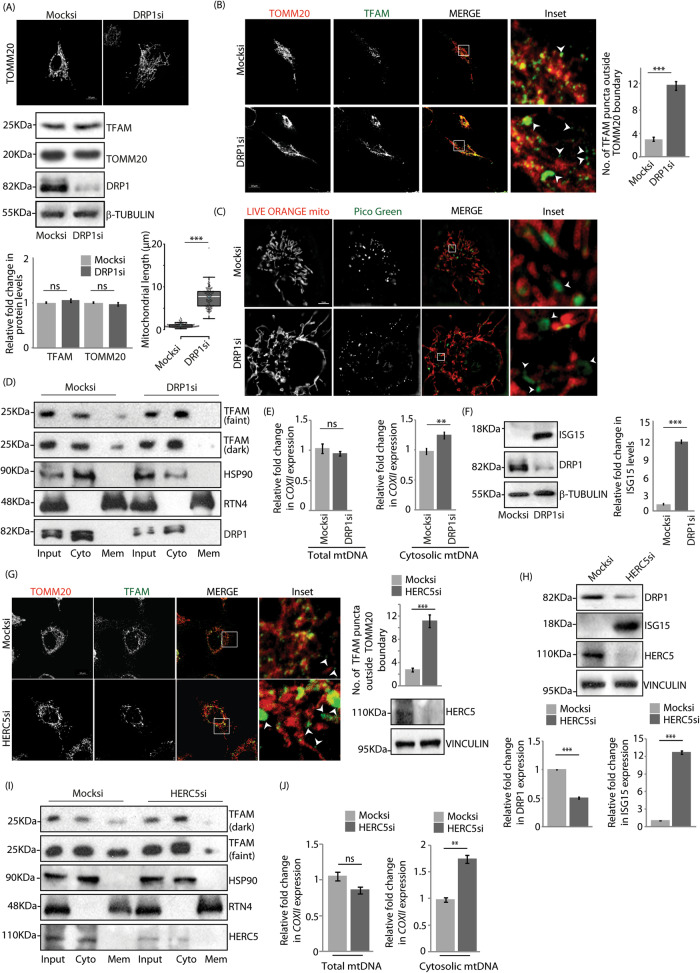
mtDNA release in presence of CoV2 PLpro phenocopy DRP1 and HERC5 depletion. **A** Cells treated with mock or DRP1 siRNAs were immunostained with anti-TOMM20 antibody and imaged. Scale bar, 10μm. Knockdown efficiency was confirmed by immunoblotting against DRP1, cell lysates were immunoblotted against TOMM20 and TFAM, β-TUBULIN was used as loading control. Graph quantifies expression of indicated proteins. Data represent the mean ± SEM of 3 independent experiments. ns, not significant (*p* > 0.2) using unpaired 2-tailed Student’s t-test. Box plot with data from image panel; the central line and the plus (+) symbol in each box show the median and mean value, respectively. ~75 cells from 3 independent experiments were analysed. ***p ≤ 0.001 using unpaired 2-tailed Student’s t-test. **B** Representative images of cells similarly treated as in panel A were immunostained against TOMM20 and TFAM. Z-stacks (0.15μm slices) were taken. Images show 3-D projections. Enlarged views of the areas within the white boxes shown (insets). White arrowheads mark TFAM puncta outside TOMM20 boundary. Scale bar, 10μm. Graph represents data from ∼100 cells from 3 independent experiments. ***p ≤ 0.001, using unpaired 2-tailed Student’s t-test. Error bars, ±SEM. **C** Cells similarly treated as in panel A were stained against LIVE ORANGE mito and PicoGreen to visualise cristae and dsDNA, respectively, and imaged in the slice 3D-SIM live mode. Enlarged views of the areas within the white boxes shown (insets). White arrowheads mark PicoGreen puncta outside LIVE ORANGE mito boundary. Scale bar, 10 μm. **D** Cytosolic and membrane fractions obtained from digitonin-permeabilised A549 cells were immunoblotted with indicated antibodies; inputs indicate total protein in cell lysates. Note increased TFAM protein levels in cytosolic fraction and decrease in membrane fraction upon DRP1 depletion, more prominent in the blot with darker exposure. HSP90 and RTN4 served as controls for cytosolic and membrane fractions, respectively. DRP1 levels indicate knockdown efficiency. **E** DNA isolated from whole cell and cytosolic extracts was analysed by qPCR. Nuclear (*GAPDH*) and mitochondrial (*COXII*) DNA was quantified using specific primers. Plots show abundance of total (left) and cytosolic (right) cellular mtDNA. ns, not significant (*p* = 0.07), **p ≤ 0.01 using unpaired 2-tailed Student’s t-test. Error bars, ±SEM. **F** Mock or DRP1 siRNAs-treated cells were immunostained for ISG15. DRP1 and β-TUBULIN confirm knockdown efficiency and equal sample loading. Graph depicts changes in ISG15 levels. Data represents 3 independent experiments. ***p ≤ 0.001 using unpaired 2-tailed Student’s t-test. Error bars, ±SEM. **G** Representative images of cells were treated with indicated siRNAs and immunostained with antibodies against TOMM20 and TFAM. Z-stacks (0.15 μm slices) were taken. Images show 3-D projections. Insets shown. White arrowheads mark TFAM puncta outside TOMM20 boundary. Scale bar, 10μm. Graph represents data from ∼75 cells from 3 independent experiments. ***p ≤ 0.001, using unpaired 2-tailed Student’s t-test. Error bars, ±SEM. Imunoblot of lysates post-imaging confirm HERC5 depletion, VINCULIN was used as loading control. **H** Lysates similarly generated as in panel G were immunostained against DRP1, ISG15, HERC5 and VINCULIN. Graphs show the changes in the expression of DRP1 and ISG15. Data represents 3 independent experiments. ***p ≤ 0.001 using unpaired 2-tailed Student’s t-test. Error bars, ±SEM. **I** Cytosolic and membrane fractions obtained from digitonin permeabilised A549 cells were immunoblotted with TFAM antibody; inputs indicate total protein in cell lysates. Note higher levels of TFAM present in cytosolic fraction of HERC5-depleted samples. Blots representative of 3 independent experiments. HSP90 and RTN4 served as controls for cytosolic and membrane fractions, respectively. HERC5 levels confirm knockdown efficiency. **J** DNA isolated from whole cell and cytosolic extracts was analysed by qPCR. Nuclear (*GAPDH*) and mitochondrial (*COXII*) DNA was quantified using specific primers. Plots show the abundance of total (top) and cytosolic (bottom) cellular mtDNA. ns, not significant (*p* = 0.06), **p ≤ 0.01, using unpaired 2-tailed Student’s t-test. Error bars, ±SEM.

Loss of DRP1 is known to result in filamentous or hyperfused mitochondria ([38], Fig. 3A); TFAM and TOMM20 levels remained unaffected upon mitochondrial elongation. However, to validate if compromise in DRP1 affects mtDNA release from mitochondria, A549 cells treated with siRNAs against DRP1 were immunostained against TOMM20 and TFAM, and imaged (Fig. 3B). Knockdown of DRP1 led to a significant increase in TFAM positive puncta outside TOMM20 boundary, compared with the control. Similar results were obtained when cells were loaded with LIVE ORANGE mito and PicoGreen to analyse the cristae and dsDNA (Fig. 3C). Further, fractions generated from siRNA-treated semipermeabilised cells indicated an increase in cytosolic TFAM levels when DRP1 was compromised (Fig. 3D). qPCR results showed a significant increase in *COXII* levels in cytosolic mtDNA when DRP1 was knocked down, compared with the control (Fig. 3E); loss of DRP1 did not significantly alter the total mtDNA content. Interestingly, compromise in DRP1 also led to an increase in the levels of ISG15 monomers (Fig. 3F). These results suggested that while reduced DRP1 activity affected mitochondrial morphology, it was accompanied with cytosolic extrusion of mtDNA and upregulation in ISG15 levels.

HERC5 knockdown condition mimicking DRP1 deISGylated situation also led to increase in mitochondrial length and volume, along with a decrease in their total numbers (Figures S4A-C). In presence of compromised HERC5, imaging studies showed significantly more number of TFAM-positive puncta outside TOMM20 boundary, suggesting cytosolic release of mtDNA (Figs. 3G, S4D). Downregulation of HERC5 also led to decrease in DRP1 and increase in ISG15 levels (Figs. 3H, S4D). Further, semi-permeabilised lysates had elevated TFAM levels in cytosolic fractions (Fig. 3I) and significantly enhanced *COXII* levels in cytosolic mtDNA (Fig. 3J). Like DRP1, loss of HERC5 did not significantly alter the total mtDNA amount. These results recapitulated the expression patterns of DRP1, ISG15 and the extrusion of mtDNA seen in the presence of CoV2 PLpro WT.

### Balance between DRP1 ISGylation and ubiquitination

**Fig. 4:**
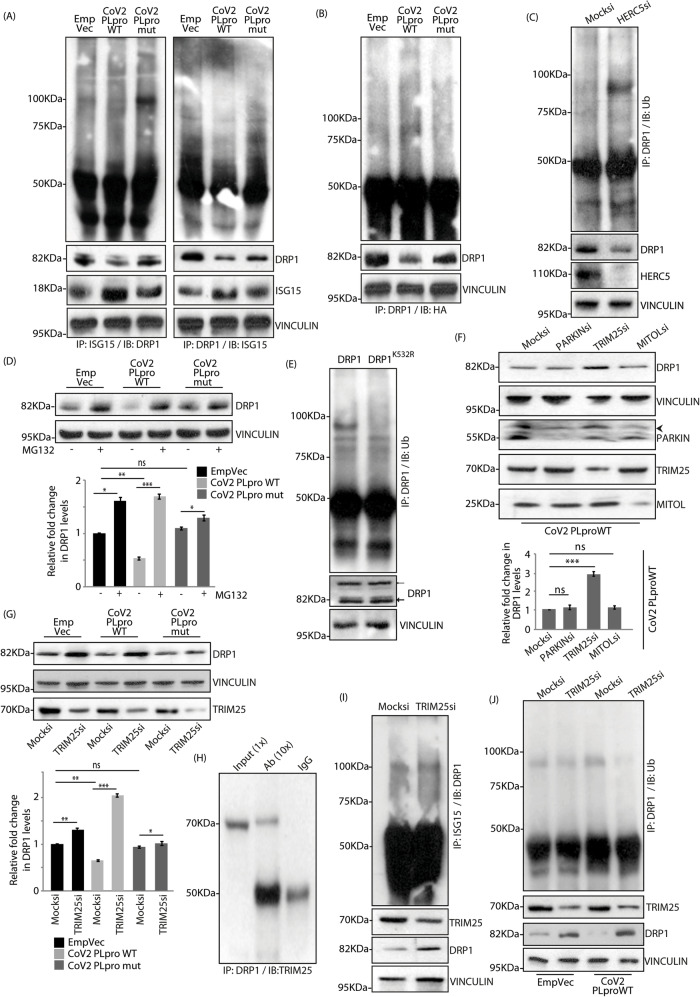
Ubiquitylation of DRP1 by TRIM25. **A** Cell transfected with the indicated GFP-tagged constructs were lysed, immunoprecipitated and immunoblotted. Note differential ISGylation profiles of DRP1 amongst the samples. Reverse co-IP confirms the same result. The input levels of DRP1, ISG15 and VINCULIN in the total lysates served as loading controls. **B** Cells transfected as in panel A were immunoprecipitated (IP) with anti-DRP1 antibody. Western blot (IB) analysis against HA-tagged Ub reveals increased DRP1 ubiquitylation in CoV2 PLpro WT samples. DRP1 and VINCULIN levels in the total lysates served as loading controls. **C** Mock or HERC5 siRNAs-treated cell lysates were immunoprecipitated (IP) with anti-DRP1 antibody. Ex vivo ubiquitylation of DRP1 was analysed by immunoblotting against Ub. Lysates were also checked for the levels of DRP1, HERC5 and VINCULIN. **D** Cells transfected with indicated GFP-tagged constructs were treated MG132 or left untreated. Note increased DRP1 levels upon drug treatment. Immunoblots were analysed against DRP1 and VINCULIN. Graph shows changes in protein levels of DRP1. Data represents 3 independent experiments. ns, not significant (p > 0.07), *p ≤ 0.05, **p ≤ 0.01, ***p ≤ 0.001 using unpaired 2-tailed Student’s t-test. **E** Cells were transfected with indicated mCherry-tagged constructs, immunoprecipitated against DRP1 and immunoblotted with Ub antibody. Note that K532R mutation in DRP1 compromises its ubiquitylation.  endogenous DRP1, ← mCherry-tagged DRP1. VINCULIN was used as loading control. **F** Cells transfected with CoV2 PLpro WT were treated with the indicated siRNAs and immunoblotted against DRP1. Knockdown efficiency was confirmed by immunoblotting against PARKIN, TRIM25 and MITOL. VINCULIN was used as loading control. Graph shows the changes in the protein levels of DRP1. Data represents 3 independent experiments. ns, not significant (*p* > 0.07), ***p ≤ 0.001 using unpaired 2-tailed Student’s t-test. **G** Mock or TRIM25 siRNAs treated cells transfected with indicated GFP-tagged constructs were immunoblotted with anti-DRP1 antibody. Note increased DRP1 levels upon TRIM25 depletion. Input levels of TRIM25 and VINCULIN served as loading controls. Graph plots protein levels of DRP1. Data represents 3 independent experiments. ns, not significant (*p* > 0.08), *p ≤ 0.05, **p ≤ 0.01, ***p ≤ 0.001 using unpaired 2-tailed Student’s t-test. **H** A549 cell lysates were immunoprecipitated against DRP1 and immunoblotted with TRIM25 antibody. The proportion of lysate loaded as input and used for immunoprecipitation is denoted in brackets by ‘X’. **I** Mock or TRIM25 siRNAs treated cells were co-immunoprecipitated against ISG15 and DRP1. Note similar ISGylated DRP1 levels across samples. TRIM25 levels in the lysates confirm knockdown efficiency, VINCULIN served as loading control. DRP1 levels were also checked in cell lysates. **J** Cells expressing the indicated GFP-tagged constructs were depleted off TRIM25 and analysed for co-IP between DRP1 and Ub. Note significantly reduced DRP1 ubiquitylation in TRIM25 depleted cells expressing CoV2 PLpro WT. Lysates were immunoblotted against DRP1, TRIM25 and VINCULIN.

To further validate the effect of CoV2 PLpro WT on DRP1 ISGylation, cell lysates were co-immunoprocipitated and probed against ISG15 and DRP1 (Fig. 4A). ISGylation of DRP1 was severely compromised in the presence of CoV2 PLpro WT. However, ISG15-modified DRP1 could be distinctly detected in the samples expressing the catalytically inactive mutant protease or the control. Interestingly, increased DRP1 ubiquitylation was seen in the presence of CoV2 PLpro WT, compared with its mutant or the control (Figs. 4B, S5A), suggesting a reverse correlation between these two post-translational modifications. When HERC5 was compromised, deISGylated DRP1 was prone to enhanced ubiquitylation (Fig. 4C). Further, when proteasomal ubiquitylation was blocked by MG132, similarly elevated DRP1 levels could be detected across all samples (Fig. 4D). Ubiquitylation of DRP1 was compromised in the presence of its mutant (DRP1^K532R^) (Fig. 4E) – suggesting that the K532 residue could be modified by ISG15 or Ub. In presence of CoV2 PLpro WT, DRP1 was substantially more ubiquitylated than DRP1^K532R^ (Figure S5B).

### Ubiquitylation of DRP1 by TRIM25 in a context-specific manner

While PARKIN and MITOL are the established Ub E3 ligases for DRP1, their loss of function did not affect DRP1 levels in the presence of CoV2 PLpro WT (Fig. 4F). Similarly, overexpression of GFP tagged PARKIN or MITOL also failed to alter DRP1 levels in samples with the wild type protease (Figures S5C, S5D). Moreover auto-ubiquitylation of PARKIN and MITOL, was compromised in the presence of the wild type protease when compared with its mutant or control (Figures S5E, S5F). Hence, in presence of CoV2 PLpro WT, which can cleave off ISG15 modifications from proteins, PARKIN is most plausibly functionally challenged. Further, co-immunoprecipitation studies detected ISGylated form of MITOL (Figure S5G) and led us to hypothesize that like PARKIN, its ligase activity could also be similarly compromised in the presence of the wild type protease. In depth analyses of this is being carried out in a separate study. These suggested involvement of another Ub E3 ligase in the degradation of DRP1. It is known that lack of ISGylation of PARKIN affects its activity [39].

Loss of TRIM25 (tripartite motif containing 25), however, altered DRP1 levels in the presence of CoV2 PLpro WT (Fig. 4F and G). TRIM25 is a lesser utilised ISG15 E3 ligase [40]; it also acts as a Ub ligase [41]. Knockdown of TRIM25 affected DRP1 levels across all samples (Fig. 4G), suggesting its plausible function in DRP1 ubiquitylation. The increase in DRP1 levels upon loss of TRIM25 was more drastic in presence of CoV2 PLpro WT, when compared with mutant or the control. The protein levels of the four ligases (PARKIN, MITOL, TRIM25 and HERC5) were similar across samples (Figure S5H). Further, when TRIM25 was overexpressed, the protein turnover of DRP1 was significantly faster that in the controls (Figure S6A). Hence, to establish the regulatory effect of TRIM25 on DRP1, we first verified interaction between the two proteins by co-immunoprecipation studies (Figs. 4H, S6B). Loss of TRIM25 did not affect the ISGylation status of DRP1 (Fig. 4I); however it led to a decrease in its ubiquitylation (Figs. 4J, S6C). In the presence of CoV2 PLpro WT, lack of TRIM25 led to almost complete loss of DRP1 ubiquitylation. When TRIM25 was compromised, decreased mitochondrial length, reduced volume and increase in their toral numbers were detected (Figures S6D-F) – these results were opposite to those observed with downregulation of HERC5 or DRP1 (Figs. 3 A, S4A).

Taken together, these suggest that while under normal circumstances PARKIN or MITOL may be the primary ligase for DRP1, when their activities are compromised (due to loss of ISG15 modifications), TRIM25 becomes the dominant ubiquitylating and regulatory enzyme. This clearly shows that while multiple Ub E3 ligases might be present for DRP1 in a cell, only one of them (TRIM25) is preferentially active in a context-specific manner (presence of CoV2 PLpro) and mediates its degradation. HERC5 and TRIM25 probably compete with each other for the same binding site on DRP1 (K532).

### Differential post-translational modifications affect DRP1’s functionality

**Fig. 5:**
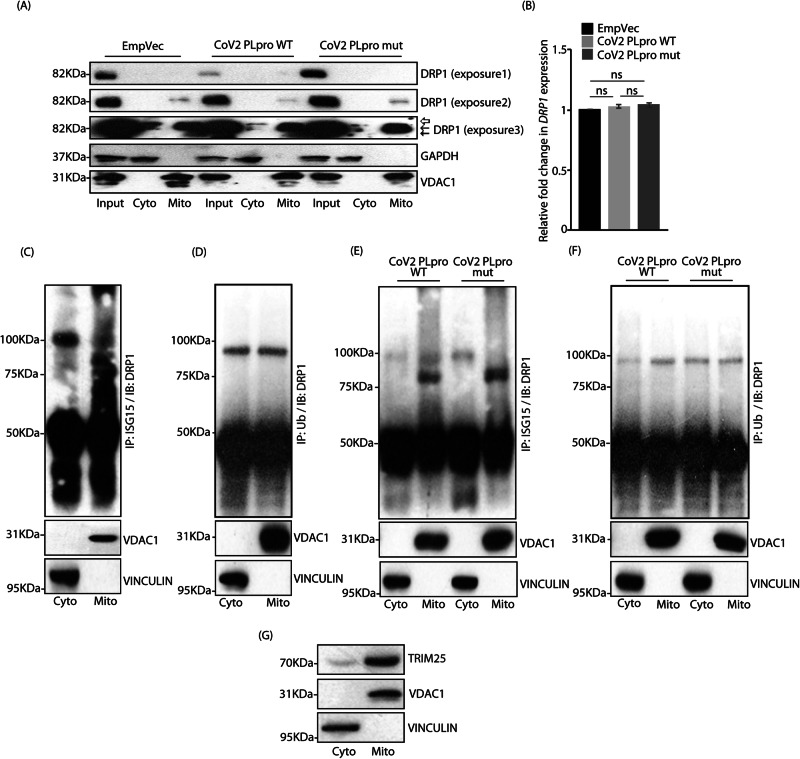
Differential post-translational modifications alter DRP1 localisation. **A** Cytosolic and mitochondrial fractions obtained from semi-permeabilised cells, transfected with indicated constructs, were immunoblotted using antibody against DRP1. GAPDH and VDAC1 served as controls for cytosolic and mitochondrial fractions, respectively. Note lower levels of DRP1 were observed in samples with CoV2 PLpro WT.  Unmodified DRP1, ← and likely to be post-translationally modified forms of DRP1. Note reduced DRP1 levels in input (exposure 1), mitochondrial (exposure 2) and cytosolic (exposure 3) fractions in CoV2 PLpro WT samples. (**B**) Total RNA isolated from A549 cells transfected with the indicated GFP-tagged constructs was subjected to qRT-PCR using primers against *GAPDH* (control) and *DRP1*. Samples were present in triplicate. 2^-ΔΔ*Ct*^ values were plotted. Graph shows results from 3 independent experiments. ns, not significant (*p* > 0.1) using unpaired 2-tailed Student’s t-test. Error bars, ±SEM. **C** Cytosolic and mitochondrial fractions obtained from semi-permeabilised cells were analysed for co-IP between DRP1 and ISG15. Note that cytosolic DRP1 pool is primarily ISGylated. VINCULIN and VDAC1 served as controls for cytosolic and mitochondrial fractions, respectively. **D** Cells fractionated as in panel C were probed for co-IP between DRP1and Ub. Note ubiquitylated DRP1 present in both the fractions. VDAC1 and VINCULIN levels in the total lysates served as loading controls. **E** Cells transfected with the indicated GFP-tagged constructs, fractionated as in panel C were divided into two. One part was immunoprecipitated with anti-ISG15 antibody and immunoblotted against DRP1. Note lower levels of ISGylated DRP1 in samples with CoV2 PLpro WT than the mutant. VINCULIN and VDAC1 served as controls. **F** Second part generated in panel E was analysed for co-IP between DRP1 and Ub. VDAC1 and VINCULIN levels confirm fractionation efficiency. **G** Cytosolic and mitochondrial fractions obtained from semi-permeabilised cells were analysed for TRIM25. VINCULIN and VDAC1 served as controls.

Since, the fission protein, DRP1 shuttles between cytosol and mitochondria, fractionated cell lysates were analysed for its levels and distribution across samples (Fig. 5A). Lowered DRP1 expression was detected in CoV2 PLpro WT samples – cytosolic as well as mitochondrial pools, though the reduction was more pronounced in the cytosolic pool. Additionally, multiple bands of DRP1, probably indicative of various post-translationally modified forms of the protein were also detected. The transcript levels of DRP1 remained unchanged across samples (Fig. 5B). These results suggested an alteration in DRP1 levels and localisation in the presence of this CoV2 protease. Co-immunoprecipitation of fractionated cell lysates indicated that the cytosolic pool of DRP1 was primarily ISGylated (Fig. 5C), while both cytosolic and mitochondrial pool could get ubiquitylated (Fig. 5D). While it is known that ubiquitylation of DRP1 leads to its degradation [42–44], it cannot be ruled out that a portion of ubiquitylated DRP1 may also be involved in regulating its activity. Further, the cytosolic pool of ISGylated DRP1 was severely compromised in the presence of CoV2 PLpro WT, compared with the mutant (Fig. 5E). The opposite was observed when probed for the Ub-mediated modification – increased ubiquitylation of mitochondrial DRP1 was detected in presence of the wild type protease when compared with the cytosolic pool (Fig. 5F). Our finding that a substantial portion of TRIM25 could partition to mitochondria (Figs. 5G, 45) further supported that mitochondrial pool of DRP1 could get ubiquitylated even when PARKIN and MITOL were rendered inactive.

**Fig. 6:**
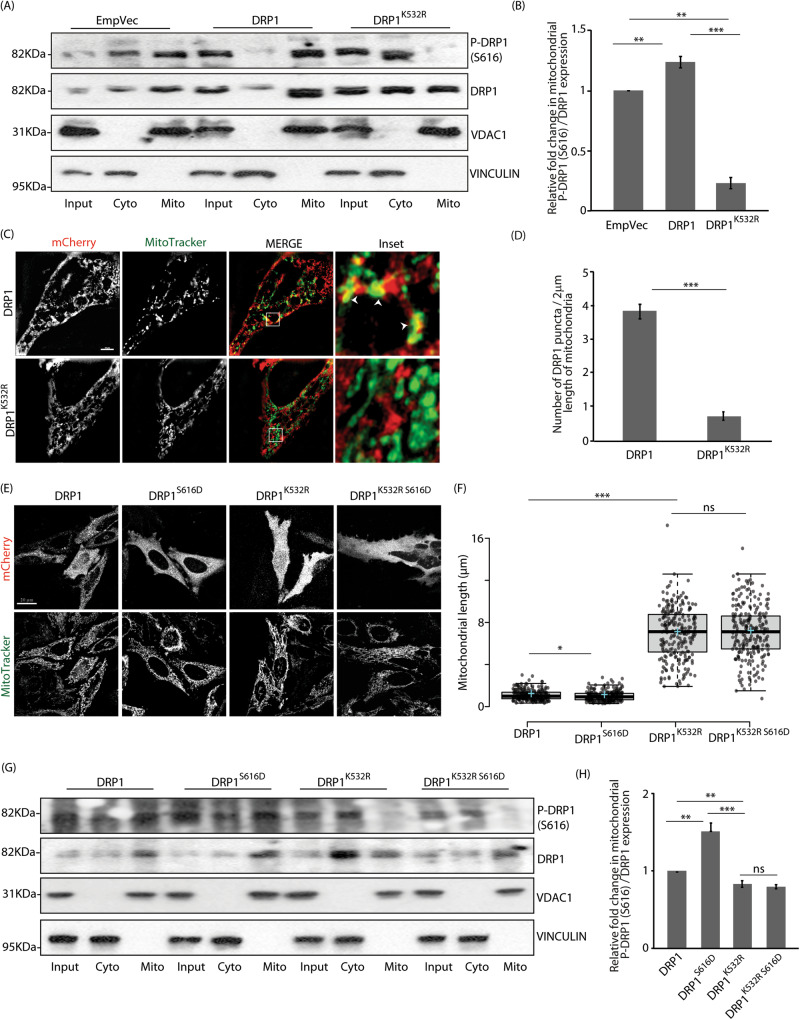
DRP1 ISGylation affects its function. **A** Cytosolic and mitochondrial fractions obtained from semi-permeabilised cells, transfected with indicated constructs, were immunoblotted against phospho-DRP1 (S616) and total DRP1; VINCULIN and VDAC1 served as controls for cytosolic and mitochondrial fractions, respectively. Note lower levels of phospho-DRP1 (S616) and DRP1 in DRP1^K532R^ mitochondrial fractions. **B** Graph represents data from 3 independent experiments. **p ≤ 0.01, ***p ≤ 0.001, using unpaired 2-tailed Student’s t-test. Error bars,±SEM. **C** Cells transfected with indicated mCherry-tagged constructs and treated with MitoTracker Green FM were imaged in the slice 3D-SIM live mode. White boxes show insets. White arrowheads mark the mCherry and mitochondria localisation. Scale bar, 10μm. **D** Graph represents number of DRP1 puncta / 2μm length of mitochondria from 3 independent experiments. ***p ≤ 0.001 using unpaired 2-tailed Student’s t-test. Error bars,±SEM. ~50 mitochondria were analysed. **E** Cells transfected with indicated mCherry-tagged constructs and treated with MitoTracker Green FM were imaged under live-cell conditions. Scale bar, 20μm. **F** Box plot showing quantification of mitochondrial length; ~100 cells from 3 independent experiments were analysed. The central line and the plus (+) symbol in each box show the median and mean value, respectively. ns, not significant (*p* = 0.6), *p ≤ 0.05, ***p ≤ 0.001 using unpaired 2-tailed Student’s t-test. **G** Cytosolic and mitochondrial fractions generated as in panel A were immunoblotted against phospho-DRP1 (S616) and total DRP1; VINCULIN and VDAC1 served as controls for cytosolic and mitochondrial fractions, respectively. **H** Graph represents data from 3 independent experiments. ns, not significant (*p* = 0.7), **p ≤ 0.01, ***p ≤ 0.001 using unpaired 2-tailed Student’s t-test. Error bars,±SEM.

Presence of DRP1^K532R^ did not affect DRP1 phosphorylations (Figs. S7A and S7B) or SUMOylation (Figure S7C). However, analyses of cell lysates showed lesser amount of phospho-DRP1 (S616) in mitochondrial fractions in the presence of DRP1^K532R^ than the wild type or the control (Fig. 6A and B). Total DRP1 at the mitochondria was also less for DRP1^K532R^ than the wild type (Fig. 6C and D). Further, co-immunoprecipitation studies with MiD49 or MFF suggested a more robust interaction of these adapters with DRP1, when compared with DRP1^K532R^ (Figures S7D, S7E) – supporting better recruitment of the wild-type fission protein onto mitochondria. Serine to aspartic acid mutants at amino acid 616 were generated in both wild-type DRP1 and DRP1^K532R^ to simulate a phospho-mimetic condition (Figures S7F and S7G, 10). DRP1^S616D^ and DRP1^K532R S616D^ showed no further change in mitochondrial length over and beyond DRP1 and DRP1^K532R^, respectively (Fig. 6E and F). The lack of an effect in the presence of the phospho-mimetic mutant of DRP1^K532R^ emphasized the necessity of ISGylation in K532 position of DRP1 to maintain its functionality. Also, mitochondrial recruitment of phospho-DRP1 (S616) was less in both DRP1^K532R^ and DRP1^K532R S616D^ mutants than DRP1 and DRP1^S616D^ (Fig. 6G and H). As expected, the amount of the fission protein recruited to the mitochondria was most in the presence of DRP1^S616D^. These results reiterate that ISGylation of DRP1 is crucial for its fission function at mitochondria.

### Relevance of DRP1 ISGylation in Alzheimer’s disease

**Fig. 7:**
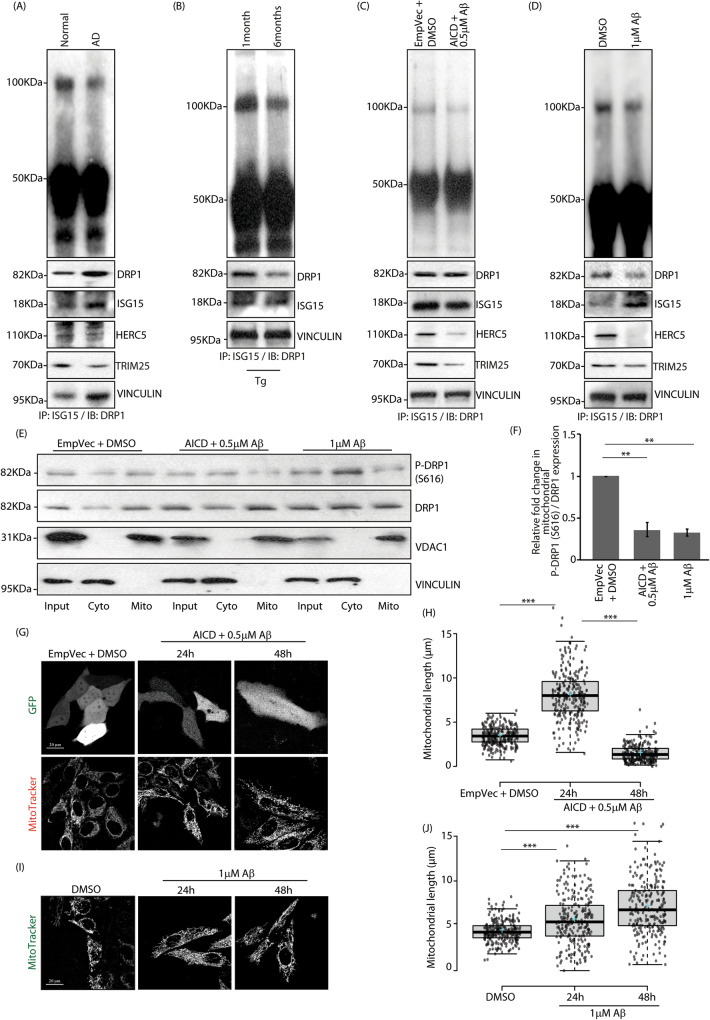
Altered DRP1 ISGylation in Alzheimer’s disease. **A** Co-immunoprecipitation against ISG15 and DRP1 was done in human brain whole tissue lysate. Note differential ISGylation profiles of DRP1 between the samples. The input levels of DRP1, ISG15, HERC5, TRIM25 and VINCULIN in the total lysates served as loading controls. **B** Brain lysates from 5XFAD mice were co-immunoprecipitated and analysed as in panel **A**. The input levels of DRP1, ISG15, and VINCULIN served as loading controls. **C** SHSY5Y cells transfected with indicated constructs were treated with DMSO and 0.5 μM Aβ for 48 h. Note reduced presence of ISGylated DRP1 in samples with AICD and Aβ, as detected by co-immunoprecipitation. The input levels of DRP1, ISG15, HERC5, TRIM25 and VINCULIN were analysed. **D** SHSY5Y cells treated with DMSO or 1 μM Aβ for 48 h were verified for DRP1 ISGylation as in panel **C**. **E** Cytosolic and mitochondrial fractions obtained from semi-permeabilised cells, transfected with indicated constructs and treated with DMSO, 0.5 μM Aβ or 1 μM Aβ for 48 h, were immunoblotted against phospho-DRP1 (S616) and total DRP1; VINCULIN and VDAC1 served as controls for cytosolic and mitochondrial fractions, respectively. **F** Graph represents data from 3 independent experiments. **p ≤ 0.01 using unpaired 2-tailed Student’s t-test. Error bars,±SEM. **G** Cells transfected with indicated GFP-tagged constructs and treated with DMSO or 0.5 μM Aβ over indicated time periods were imaged with MitoTracker Red FM under live-cell conditions. Scale bar, 20μm. **H** Box plot showing quantification of mitochondrial length; ~125 cells from 3 independent experiments were analysed. The central line and the plus (+) symbol in each box show the median and mean value, respectively. ***p ≤ 0.001 using unpaired 2-tailed Student’s t-test. **I** Cells treated with DMSO or 1 μM Aβ for indicated time periods were imaged as in panel G. Scale bar, 20μm. **J** Mitochondrial length from ~120 cells were analysed and plotted from 3 independent experiments. The central line and the plus (+) symbol in each box show the median and mean value, respectively. ***p ≤ 0.001 using unpaired 2-tailed Student’s t-test.

To understand the pathophysiological relevance of DRP1 ISGylation, we explored its status in one of the neurodegenerative disorders, Alzheimer’s disease (AD). This was analysed in patient and transgenic mice model of AD-derived samples as well as ex vivo. First, in human brain lysates, DRP1 ISGylation was observed to be less in AD samples than the normal counterpart (Fig. 7A). Secondly, in transgenic 5XFAD mice model of AD, a decrease in the levels of ISGylated DRP1 was detected in brain lysates from aged individuals when compared with the younger ones (Fig. 7B). Here, brain lysates from 1month (young) and 6months (aged) old mice were compared. This is because the 6-month-old mice show disease manifestation, while the younger ones do not [46]. Thirdly, we analysed a cell-based model of AD, generated by treating SHSY-5Y cells with both AICD (amyloid precursor protein intracellular domain) and beta-Amyloid-[1,2,3,4,5,6,7,8,9,10,11,12,13,14,15,16,17,18,19,20,21,22,23,24,25,26,27,28,29,30,31,32,33,34,35,36,37,38,39,40,41,42] or Aβ_1–42_ protein fragment (referred as Aβ) for 48 h [47]. Here, we observed a similar decrease in DRP1 ISGylation in the ex vivo AD model lysates when compared with the controls (Fig. 7C). Finally, SHSY-5Y cells exposed to 1 μM Aβ for 48 h [48] again showed less ISGylation of DRP1 in the treated samples (Fig. 7D). HERC5 and TRIM25 levels were lower in human brain lysates and cell models of AD (Fig. 7A, C and D). Mitochondrial recruitment of phospho-DRP1 (S616) was found to be reduced in cell lysates treated with either, AICD and 0.5 µM Aβ together, or with only 1 µM Aβ (Fig. 7E and F). An increase in mitochondrial length was observed in cells with AICD and Aβ at 24 h; however, at 48 h shorter mitochondria were more abundant (Fig. 7G, H). In 1 µM Aβ treated cells, mitochondrial filamentation was seen in cells both at 24 h and 48 h (Figs. 7I and J). Total ATP produced was significantly less in cells with elongated mitochondria (AICD and 0.5 µM Aβ treatment for 24 h or exposure to1µM Aβ for similar time); we also detected compromised mitochondrial contribution in the metabolic profile of these cells (Figures S8). The results taken together show that decreased ISGylation could be one of the contributing factors in DRP1 functionality and mitochondrial dynamics in a disease background. Our results in the cell systems further, reiterate that phospho-DRP1 (S616) levels at the organelle are more significant than the total protein in initially driving miotochondrial fission. As reported previously [2, 3] when the load of stress increases, hyperfused mitochondria are pushed towards fission in a DRP1-independent manner.

## Discussion

**Fig. 8:**
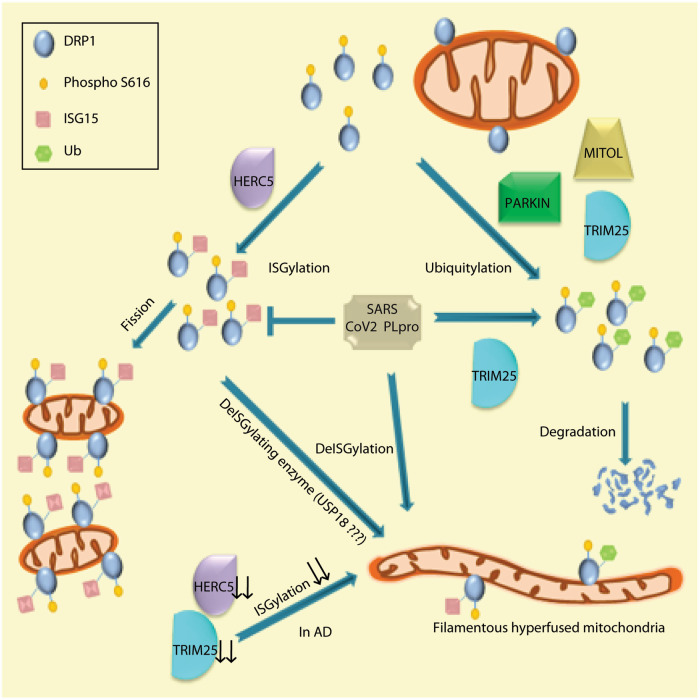
Schematic diagram summarising the results. ISGylation of DRP1 [more specifically phopho-DRP1 (S616)] renders it fission competent. Mitochondrial filamentation occurs due to lack of this post-transaltional modification (as in Alzheimer’s disease, AD scenario) or in the presence of SARS-CoV2 PLpro (a deISGylating enzyme); USP18 could also impart deISGylation in cells. DRP1 is primarily ISGylated by HERC5 leading to fission; Ubiquitin E3 ligases, like PARKIN, MITOL and TRIM25 ubiquitylate and degrade DRP1 to promote fusion. In AD reduced expression of HERC5 and TRIM25 results in compromised DRP1 ISGylation and activity.

This study, while unravelling ISGylation to be a new post-translational modification of DRP1, actually addresses some of the fundamental questions on protein quality control and localisation. First, we report that presence or absence of DRP1 ISGylation alters mitochondrial homeostasis. With increased DRP1 ISGylation, broken mitochondria are found more in number. Just the opposite phenomenon is observed when DRP1 ISGylation is inhibited; mitochondria become filamentous. Secondly, CoV2 PLpro, which is an established deISGylating viral protein also affects mitochondrial dynamics and associated with a decrease in the total DRP1 levels in cells. There is release of mtDNA into the cytosol and associated with this is induction of the IFN-I response genes via the cGAS-STING pathway. Activation of these response genes due to presence of CoV2 PLpro could be one of the contributing factors during SARS CoV2 infection. Thirdly, we show that while a substrate might have multiple E3 Ub ligases that can help post-translationally modify it, their activity is determined in a context-specific manner – not all ligases may be equally active at the same time for a substrate. The presence of CoV2 PLpro acts as the determining factor, whose ability to deISGylate proteins renders PARKIN and MITOL inactive. Hence, TRIM25 emerges as an opportunistic E3 Ub ligase for DRP1. Fourth, ISGylation, ubiquitylation and SUMOylation can compete against one another for the same lysine at site 532 of DRP1 (K532), though they may or may not modify at other sites as well. Here we show for the first time that ISGylation of S616 phosphorylated DRP1 is essential for its ability to reach the mitochondria and promote fission. Compromised DRP1 ISGylation could be a contributing factor in pathogenesis of AD. Identification of DRP1 ISGylation in various cellular, animal and human contexts and resulting hyperfusion when it is compromised emphatically suggests the universality of this post-translational modification and its significance in maintaining mitochondrial homeostasis (Fig. 8).

Destabilisation of mitochondrial fission-fusion balance and the eventual effect on the morphology of the organelle is well established [1]. This is primarily brought about by the deregulation of the key molecular players maintaining the fission-fusion dynamics. Post-translational modifications of these mitochondrial proteins are the chief contributing factors that preserve this balance [49]; some of the important modifications include phosphorylation, O-GlcNAcylation, acetylation, ubiquitylation and SUMOylation [50]. It is known that the function and turnover of the mitochondrial fission protein, DRP1 is regulated by post-translational modifications, like phosphorylation, SUMOylation, ubiquitylation and S-nitrosylation [2, 10]. While phosphorylation of DRP1 alters its activity, ubiquitylation promotes degradation. Increased ubiquitylation and degradation of DRP1 is known to promote mitochondrial hyperfusion [2, 3, 42, 44]. HERC5 is the major ISG15 E3 ligase in cells, with a partial contribution from TRIM25 [40]. Our study identifies a new target DRP1, which gets post-translationally modified by ISG15 with HERC5 being the E3 ligase. Manipulating the ISGylation status of DRP1, which is a mitochondrial fission protein, regulates the mitochondrial dynamics.

The SARS CoV2 nsp3 protease PLpro has recently been demonstrated to harbour deISGylase activity [21]. Elevated levels of monomeric ISG15 supported this. In the presence of CoV2 PLpro decrease in total DRP1 levels and presence of filamentous mitochondria reinforces the importance of ISGylation. Extra-mitochondrial localisation of mtDNA is progressively emerging as a key regulator of the immune response, especially during viral infections and in the maintenance of cellular homeostasis [30]. Cytosolic mtDNA interacts with the DNA sensor, cGAS and activates its downstream signalling via the cGAS-STING pathway; this ultimately elicits IFN-I immune response [29]. While multiple viral infections are associated with the release of mtDNA into the cystol [28], the exact mode of this efflux remains almost unknown. Evidences so far suggest that destabilisation of the outer mitochondrial membrane plays a crucial role in the release of mtDNA into the cytosol [24–26]; much less is known about the status of the inner membrane during this process. Our present study suggests that compromised fission (due to loss of DRP1) affects mitochondrial homeostasis and eventually leads to mtDNA release into the cytosol. The cytosolic mtDNA induces IFN-I immune response. The exact mechanism of membrane destabilisation due to compromise in DRP1 is being carried out in a separate study. IFN-I-driven gene expression and immune response is suggested to affect neuroinflammation, synaptic loss and neurodegeneration in AD and other tauopathies [51, 52].

Decreased levels of ISGylated DRP1 in presence of CoV2 PLpro, along with increase in the ubiquitylated form of this mitochondrial fission protein suggested the presence of a critical balance between these two post-translational modifications. Evidence in the literature supports an inverse correlation between ISG15-mediated and Ub-mediated proteins modifications, whereby the two modifications can compete for the same binding sites on specific proteins to regulate their fate [19, 53, 54]. Mixed chains of ISG15 and Ub have also been shown to affect protein homeostasis [55]. It is plausible that lower levels of the ISGylated fission protein, promotes its ubiquitylation and in turn would lead to decrease in the total protein levels of DRP1. Similarly, SUMOylation of DRP1 has been reported to inhibit its ubiquitylation; this affects mitochondrial dynamics, ER tubulation, and plays a significant role in neuronal differentiation and corticogenesis [56].

PARKIN and MITOL are well-established Ub E3 ligases for DRP1 [42, 44]. TRIM25 can also act as an Ub E3 ligase [57]. Our study failed to detect a role for either PARKIN or MITOL in modifying DRP1. The enzymatic activity of PARKIN is dependent on its ISGylation status [39]. Further, our results show that MITOL can also get ISGylated; indirectly suggesting that the active enzyme consists of the ISG15-modified form. It is plausible that in the presence of CoV2 PLpro, PARKIN and MITOL are rendered inactive due to cleavage of ISG15. Detailed analyses to establish that ISGylation of MITOL is essential for its activity are beyond the scope of this study. However, compromised auto-ubiquitylation of PARKIN and MITOL does suggest a negative effect of this deISGylating protease on the enzymatic activities of these ligases. We went ahead to speculate that in such a context, TRIM25 could have Ub E3 ligase activity for DRP1. In presence of CoV2 PLpro, when proteins are essentially present in their deISGylated forms, TRIM25 emerges as the E3 ligase responsible for DRP1 ubiquitylation and degradation. Moreover, TRIM25 can also localise to mitochondria. This also brings forth a very significant aspect of protein quality control – while multiple E3 ligases exist for each substrate, their activities are regulated in a context-specific manner. When PARKIN and MITOL are functionally impaired, DRP1 protein turnover is maintained by TRIM25.

Further, ISG15 modification of Filamin B (FLNB) alters its localisation – post-translationally modified FLNB localises on membrane ruffles and promotes cell survival. The unmodified protein, on the other hand, has cytosolic localisation and participates in cell death [58]. DRP1 present in the cytosol is primarily ISGylated. Ubiquitylated DRP1, on the other hand may have cytosolic or mitochondrial localisation – this is probably destined for degradation. ISGylated pool of DRP1 being cytosolic in localisation also supports the hypothesis that this modification for some proteins enhances their stability; knocking down ISG15 or its conjugates promotes protein polyubiquitylation and degradation [54].

It is already established that phophorylation of DRP1 at S616 is crucial for its shuttling to mitochondria and causing fission [59]. SUMOylation of DRP1 is also suggested for its mitochondrial localisation [60]. However, the DRP1-4KR SUMOylation mutant that does not translocate to the mitochondria also lacks the K532 residue, responsible for its ISGylation. Lack of ISGylation of DRP1 (due to the presence of K532R) compromises mitochondrial localisation of phospho-DRP1 (S616) as well as drastically reduces the fission capability of the protein. The phopho-mimetic mutant (DRP1^K532RS616D^) cannot reverse the effects. Moreover, compromised interaction between DRP1^K532R^ and the fission adaptors (MiD49 and MFF) further supports the importance of ISGlyation in making DRP1 fission competent.

Along with these observations, DRP1 ISGylation is found to be physiologically relevant. In AD, one of the prominent neurodegenerative disorders, DRP1 ISGylation is found to be less. Studies in brain lysates (human and mice) and experiments on cell based models yielded similar results. While till recently, research had indicated that inhibiting the fission capability of DRP1 (by mdivi-1, mitochondrial division inhibitor) was responsible for inhibiting apoptosis [61]; however, it is now suggested that the phenotypic outcome is due to alterations in ROS production or Ca^2+^ homeostasis [62–64]. Preventing mitochondrial fragmentation by inhibiting DRP1 has also been demonstrated to elicit a delayed disease progression in multiple neuropathologies [65–67] and cardiovascular ailments [68–70]. While the age of onset or the final pathological outcome of the neurodegenerative disease remained unchanged, it is justified to suggest that DRP1 (along with its associated partners) contributes only partially to the process of fission. In situations, when the fission process fails and filamentous mitochondria prevail, DRP1-independent fission sets in [71, 72]. Enhanced mitochondrial hyperfusion in the presence of AICD and Aβ, followed by increased fragmentation, even when fission-competent DRP1 levels [phospho DRP1 (S616)] are low at the organelle clearly supports this argument. Moreover, the simultaneous occurrence of two phenomena – (i) hyperfused mitochondria and (ii) altered ATP production in ex vivo AD models – further suggests that contrary to expectation, mitochondrial filamentation leads to metabolic stress. Mitochondria, so primed are susceptible to fission. Hence, it is plausible to extrapolate that ISGylation of DRP1 acts as a switch that shuttles phospho DRP1 (S616) to mitochondria to promote fission and maintain regular cellular homeostasis. A compromise in this modification triggers a catastrophic mitochondrial fragmentation response, ultimately culminating in cellular death and disease phenotype.

Though sparse, present literature suggests that HERC5 and TRIM25 levels are affected in multiple neurodegenerative conditions. CpG methylation on HERC5 gene, suggesting its downregulation is seen in late-onset AD cases, when compared with the normal controls [73]. A mutation in TRIM25 is associated with early-onset autosomal dominant dementia and amyloidogenesis [74]. Further, RNA-seq data suggests low abundance of HERC5 and TRIM25 in brain samples analysed from individuals with aging, dementia or with traumatic brain injury, when compared with healthy subjects [75]. Decrease in protein levels of HERC5 and TRIM25 in human AD brain lysates, as well as in cell models clearly fits with the existing literature. This supports our observation of reduced DRP1 ISGylation in AD.

We can hence, appreciate that a close interplay between multiple post-translational modifications of DRP1 governs its differential localisation, modulates its function and affects its turnover – this in turn maintains homeostasis at the organellar, cellular and organismal levels.

### Limitations of the study

Our study shows that loss of DRP1 leads to not only mitochondrial hyperfusion, it is also associated with the cytosolic release of mtDNA. While this phenomenon is seen in presence of SARS-CoV2 PLpro as well as knocking down DRP1, it remains to be clearly delineated what prompts mtDNA release under these circumstances. It is likely that filamentation of mitochondria could affect membrane integrity of the organelle as implied by the presence of altered cristae architecture. This, however, needs to be experimentally ascertained. Also, further analyses need to be done to characterize the ubiquitylated DRP1 pool– whether this is destined for degradation in its entirety or a part of it has a regulatory role at the organelle.

## Experimental procedures

### Constructs, antibodies and reagents

GFP-tagged CoV2-PLpro constructs (WT and C111S mut) were gifts of Ivan Dikic (Frankfurt, Germany); HA-tagged wild type Ubiquitin was a gift of Rafael Mattera (Bethesda, MD, USA); PARKIN GFP was a gift from Noriyuki Matsuda (Tokyo, Japan); TRIM25 GFP was gift of Santosh Chauhan (Hyderabad, India). MITOL GFP (62039) and mCherry-tagged DRP1 (49152) constructs were purchased from Addgene. DRP1^K532R^, DRP1^S616D^ and DRP1^K532R S616D^ were generated by standard site-directed mutagenesis method in the mCherry-DRP1 construct (which encodes for isoform 3 of the protein). Amyloid precursor protein intracellular domain (AICD-GFP) construct and Aβ_1–42_ protein fragment (Sigma, A980) were kind gifts from Debashis Mukhopadhyay (Kolkata, India). ISG15-HA was subcloned from pCMV6-Neo-ISG15 (Addgene, 80404) using standard cloning techniques.

All antibodies used, gifts or commercially available, are included in the Supplemental experimental procedures.

All reagents used are included in the Supplemental experimental procedures.

### Cell culture, Imaging, Western blotting and Immunoprecipitation

Culture of A549, HepG2 and SHSY-5Y transient transfections, immunofluorescence staining and confocal microscopy were followed as before [2, 3, 76–78]. Western blotting and immunoprecipitation were done as previously described (2, 3, 77, 78, See Supplemental experimental procedures).

### Knockdowns with siRNA

siRNAs were transfected using Lipofectamine 2000 following the manufacturer’s instructions. In brief, cells were harvested 72 h post transfection of siRNAs, unless indicated otherwise. List of all the siRNAs used are included in Supplemental experimental procedures.

### Quantitative reverse transcription and real-time PCR

RNA extracted from cells was analysed as described before [78]. Details of all the primers used are in the Supplemental experimental procedures.

### Preparation of cytosolic and mitochondrial fractions

Cells were lysed with mitochondrial isolation buffer as previously described 77, see Supplemental experimental procedures).

### Semi-permeabilisation assay

Semi-permeabilisation and biochemical fractionation by selective detergent extraction has been described (79, see Supplemental experimental procedures).

### Quantification of mtDNA release by qPCR

In brief, lysates generated by selective detergent extraction were separated into cytosolic and mitochondrial fractions. mtDNA was isolated and analysed as described ([80, 81], see Supplemental experimental procedures).

### ATP measurements

The protocol for ATP measurement adapted from SCENITH (Single Cell ENergetIc metabolism by profilIng Translation inhibition) [82]. For detailed protocol, see Supplemental experimental procedures.

### Supplementary information

Supplementary materials

Reproducibility Checklist

Original Data File

## Data Availability

All data generated or analysed during this study are included in this published article [and its supplementary information files]. The materials included in this study are available from the corresponding authors upon reasonable request.
