# Publisher Correction: ISGylation of DRP1 closely balances other post-translational modifications to mediate mitochondrial fission

**DOI:** 10.1038/s41419-024-06857-6

**Published:** 2024-07-09

**Authors:** Palamou Das, Oishee Chakrabarti

**Affiliations:** 1 Biophysics & Structural Genomics Division, Saha Institute of Nuclear Physics, 1/AF Bidhannagar, Kolkata, 700064 India; 2 Homi Bhabha National Institute, Mumbai, India

**Keywords:** Ubiquitylation, Protein quality control

Correction to: *Cell Death and Disease* 10.1038/s41419-024-06543-7, published online 02 March 2024

Since the publication of this paper the author has noticed an error in the “Results” section, subheading ‘Relevance of DRP1 ISGylation in Alzheimer’s disease’, this has now been corrected.

The publisher would like to apologise for any inconvenience caused.

The original article has been corrected.

