# Supplementary material for: ISGylation of DRP1 closely balances other post-translational modifications to mediate mitochondrial fission
Source: Cell Death Dis. 2024 Mar 2;15(3):184. doi: 10.1038/s41419-024-06543-7 (PMC10908869; doi:10.1038/s41419-024-06543-7)
Supplement: Supplementary file 1 — Supplementary materials [file 41419_2024_6543_MOESM1_ESM.pdf]

Supplemental experimental procedures

Constructs, antibodies and reagents

GFP-tagged CoV2-PLpro constructs (WT and C111S mut) were gifts of Ivan Dikic (Frankfurt, Germany); HA-tagged wild type Ubiquitin was a gift of Rafael Mattera (Bethesda, MD, USA); PARKIN-GFP was a gift from Noriyuki Matsuda (Tokyo, Japan); TRIM25 GFP was gift of Santosh Chauhan (Hyderabad, India). MITOL-GFP (62039) and mCherry-DRP1 (49152) constructs were purchased from Addgene. $DRP1^{K532R}$, $DRP1^{S616D}$ and $DRP1^{K532R\ S616D}$ were generated by standard site directed mutagenesis method in the mCherry-DRP1 construct (which encodes for isoform 3 of the protein). Amyloid precursor protein intra-cellular domain (AICD-GFP) construct and $A\beta_{1-42}$ protein fragment (Sigma, A980) were gifts from Debashis Mukhopadhyay. ISG15-HA was subcloned from pCMV6-Neo-ISG15 (Addgene, 80404) using standard cloning techniques.

Antibodies were from the following sources: TFAM (Abcam, ab176558 and Cell Signaling Technology, 8076), TOMM20 (Abcam, ab56783), β-ACTIN (Abcam, ab8226), HSP90 (Abcam, ab13492), RTN4 (Abcam, ab47085), VDAC1 (Abcam, ab15895), VINCULIN (Abcam, ab129002), MFN1 (Abcam, ab57602), MFN2 (Cell Signaling Technology, 11925), OPA1 (Thermo Fisher Scientific, MA5-16149), STOML2 (Abcam, ab191884), FIS1 (Thermo Fisher Scientific, PA22142), MFF (Abcam, ab81127), MiD49 (Abcam, ab101350; SMCR7), DRP1 (Cell Signaling Technology, 8570 and Abcam, ab56788), ISG15 (Abcam, ab227541), GAPDH (Abcam, ab8245), β-TUBULIN (Abcam, ab7792), HERC5 (Invitrogen 703675), Ubiquitin (Sigma 050M1509 and Abcam, ab134953), TRIM25 (Abcam, ab167154), MARCH5/MITOL (Cell Signaling technology, 19168), HA (Novus Biologicals, MAB0601), PARKIN (Sigma, P6248), SUMO1 (Sigma-Aldrich S8070). GFP and RFP antibodies were gifts of Ramanujan S Hegde (Cambridge, UK). MitoTracker Red FM (Invitrogen, M22425), MitoTracker Green FM (Invitrogen, M7514) and Quant-iT™ PicoGreen™ dsDNA Assay Kits and dsDNA Reagents (P7589) was from Invitrogen. LIVE ORANGE mito (LVORANGE-0146) was from abberior.

Human IFNα1 (Cell Signaling Technology, 8927) was used at 10ng/ml concentration for 48h. Universal FastStart Syber Green Master (Rox) was from Roche, TRIzol reagent was from Invitrogen. Cycloheximide (Chx) was from Sigma Aldrich; treatment with Chx (100 μg/ml) was for the indicated time points. Luminescent ATP detection assay kit was from Abcam (ab113849), 2-Deoxy-D-glucose (2-DG) was from TCI (154-17-6), Oligomycin A was from Sigma-Aldrich (75351).

Cell culture and transfection

A549 (lung adenocarcinoma-derived hypotriploid alveolar basal epithelial cells) and HepG2 (human hepatoma cell line) cell lines were used. Maintenance of cells in culture was as before (76). Briefly, cells were grown in 10% fetal bovine serum (FBS; Gibco, Grand Island, NY, USA)/Dulbecco's modified Eagle's medium (DMEM; Himedia, Mumbai, India) at 37°C and 5% CO2. A549, gift of Amit Pal (Kolkata, India); HepG2 cells, gift of Soumen Kanti Manna (Kolkata, India) were grown under standard cell culture conditions. For transfections of cells, Lipofectamine 2000 (Invitrogen, Carlsbad, CA, USA) was used as per the manufacturer's instructions. 24h post-transfection, cells were lysed in suitable buffers. All tissue culture plasticware used for microscopy were from Nunc, Rosklide, Denmark, and bottom coverglass dishes used were from SPL Lifesciences, Gyeonggi-do, Korea.

Brain lysates of human and mice

Human brain whole tissue lysates of normal (adult whole normal; NB820-59177) and AD (adult whole Alzheimer's; NB820-59363) were from Novus Biologicals. 5XFAD transgenic mice brain lysates were gifted by S. C Biswas (Kolkata, India).

siRNA-mediated knockdown experiments

Non-targeting siRNA (D-001810-01-20) was ON-TARGETplus SMARTpool siRNA from Thermo Fisher Scientific (Dharmacon Products, Lafayette, CO, USA), siRNAs were purchased from the following resources: MITOL (4392420) from Ambion Silencer Select^{TM}, USA; DRP1 (hs.Ri.DNM1L.13.1), HERC5 (hs.Ri.HERC5.13.3), TRIM25 (hs.Ri.TRIM25.13.1), PARKIN (hs.Ri.PARK2.13.3), ISG15 (hs.Ri.ISG15.13.1), were from IDT (Integrated DNA Technologies). ON-TARGETplus SMARTpool siRNAs were from Dharmacon (USA) against HERC5 (L-005174-00-0005), TRIM25 (L-006585-00-0005). siRNAs were transfected using Lipofectamine 2000 following the manufacturer's instructions. For knockdown studies, cells were harvested 72 hours post transfection. Cells were transfected 48h post-treatment with siRNAs, if required.

Immunocytochemistry

For immunocytochemistry, cells were fixed with either 4% formaldehyde or methanol as per the requirement of the antibody, like before (76). Cells were permeabilized using 10% FBS/PBS/0.1% saponin (Sigma- Aldrich) for 60 min, followed by overnight staining in

primary antibody at 4°C and 60 min incubation in secondary antibody at room temperature. The samples were then imaged using confocal microscopes.

Western blotting and immunoprecipitation

The protocol for western blotting was as described before (77, 78). 10% or 12% Tris-tricine gels were used for SDS PAGE followed by Western blotting. Quantification of Western blots was done using Gel Quant and Image J softwares. At least 3 independent experiments were performed and band intensities were normalized to loading control. p-values were determined using Student's t-test. For immunoprecipitation, cells were lysed in immunoprecipitation buffer (50mM Tris-Hcl, pH 7.5, 150mM NaCl, 0.1% Triton X-100, 1% IGEPAL, 1mM PMSF, protease inhibitor cocktail (Sigma Aldrich), and immunoprecipitation was performed under denaturing conditions as described before.

Confocal imaging and image analyses

Confocal imaging was done using the Zeiss LSM980, Nikon A1R+ Ti-E microscope system. Ar-ion laser (for GFP excitation or Alexa-Fluor 488 with the 488 nm line), a He-Ne laser (for RFP, Alexa-Fluor 594 excitation with the 561 line were used with 100×0.55 NA oil immersion objective and 60×1.4 NA water immersion objective. He-Ne laser for Alexa-Fluor 633 with the 633 line was also used with the same objective. Cells were imaged in CO_2 independent media maintaining conditions of live-cell imaging as described before (77). Cells were imaged taking z-stacks with z interval of 0.15 μm. Image analyses and 3D projections were done in FIJI. Quantitative analysis was done for 50-200 cells (as indicated) from more than 3 independent experiments.

Mitochondrial length measurement was done as discussed before (2). For TFAM puncta calculation, z-stack images were projected in 3D. TFAM puncta outside TOMM20 boundary was calculated manually.

Mitochondrial volume and number measurement were done using ImageJ (Mitochondria Analyzer).

3D structured illumination microscopy (3D-SIM)

3D-SIM images were obtained using Nikon N-SIM on Eclipse Ti Inverted Microscope equipped with a Piezo stage, a Plan Apochromat 100x (NA 1.49) oil-immersion objective and 405/488/561/647 nm diode lasers. Image stacks were recorded with a z-distance of 125 – 150 nm. 15 images (5 different phases X 3 different angles) were captured for each XY plane;

final images were reconstructed from these 15 raw images. 3D reconstruction and alignment were performed using NISElements AR software with N-SIM module.

Quantitative reverse transcription and real-time PCR

Cells were harvested and RNA was extracted as previously described (78). Quantitative reverse transcription-PCR (qRT-PCR) was used to compare the expression of

DRP1 (forward primer, 5′- GTGAGGCAGGAGAATTGCTT-3′; reverse primer, 5′-TTGAGACGGAGTTTCGCTCT-3′),

IFNB1 (Interferon beta 1; forward primer, 5′-CAGCATCTGCTGGTTGAAGA -3′; reverse primer, 5′- CATTACCTGAAGGCCAAGGA -3′),

ISG15 (nterferon-stimulated gene 15, forward primer, 5′-CTCTGAGCATCCTGGTGAGGAA -3′; reverse primer, 5′-AAGGTCAGCCAGAACAGGTCGT-3′),

IRF7 (Interferon regulatory factor 7, forward primer, 5′- GGGTGTGTCTTCCCTGGATA -3′; reverse primer, 5′- GCTCCATAAGGAAGCACTCG -3′),

IFIT1 (Interferon-induced protein with tetratricopeptide repeats 1, forward primer, 5′-TCTCAGAGGAGCCTGGCTAA -3′; reverse primer, 5′- TCAGGCATTTCATCGTCATC-3′), and

housekeeping gene *GAPDH* (glyceraldehyde-3-phosphate dehydrogenase, forward primer, 5′-GACAGTCAGCCCGCATCTTCT-3′; reverse primer, 5′- GCGCCCAATACGACCAAATC-3′).

Preparation of cytosolic and mitochondrial fractions

Cells were lysed in mitochondrial isolation buffer (10 mM HEPES (Sigma Aldrich, H3375)-KOH buffer [pH 7.4] containing 0.22 M mannitol (Sigma Aldrich, M4125), 0.07 M sucrose (Sigma Aldrich, S8501), by passing through a 25G needle attached to a 1ml syringe at least 10 times. A small fraction of this was saved as the whole-cell lysate. This was centrifuged at 600g to pellet unlysed cell debris and nuclear fractions. The remaining solution was centrifuged at 4000g to get the mitochondria enriched fraction, which was then washed twice with isolation buffer. The supernatant was collected as the cytosolic fraction.

Semi-permeabilization assay

Biochemical fractionation by selective detergent extraction has been described before (79). In brief, cells were washed with PBS twice and treated with PBS containing digitonin (at 100

ug/ml; 78) to generate the soluble cytosolic fraction. After collecting the cytosolic fraction, cells were scraped off the dishes with lysis buffer (50mM Tris pH 7.5, 150mM NaCl, 2mM EDTA, 0.5% TritonX-100, 0.5% Sodium deoxycholate) and centrifuged at 14200 RPM for 15 minutes. The supernatant was used as the membrane fraction. The cytosolic fractions were TCA precipitated, analyzed by SDS-PAGE, followed by immunoblotting against the indicated antibodies.

Quantification of mtDNA release by qPCR

The protocol was as described before (80). In brief, 24h post-transfection, cells were washed with PBS and treated with 1% NP-40 and scraped. Lysates were kept in ice for 15min followed by centrifugation at 16000g at 4^0C for 15min. The supernatant containing the cytosolic fraction was used to purify mtDNA as per the manufacturer's instructions [DNA Blood and Tissue kit (Qiagen)]. The pellet was used to isolate genomic DNA. After DNA isolation the samples were used for qPCR with mtDNA specific primers; mitochondrially encoded genes

COXII (Cytochrome c oxidase subunit II, forward primer, 5′-ATCAAATCAATTGGCCACCAATGGTA-3′; reverse primer, 5′-TTGACCGTAGTATACCCCCGGTC-3′), and

mtND1 (forward primer, 5′- CCCTAAAACCCGCCACATCT-3′); reverse primer, 5′-GAGCGATGGTGAGAGCTAAGGT-3′),

nuclear encoded gene *GAPDH* (forward primer, 5′- GACAGTCAGCCCGCATCTTCT-3′, reverse primer, 5′- GCGCCCAATACGACCAAATC-3′).

Calculations were done as before (81).

Statistical analyses

For all experiments, at least 3 biological replicates were used unless otherwise indicated. Graphs have been plotted by exporting data of ImageJ to MS Excel 2007. Error bars represent mean±SEM, 2-tailed type 3 Students *t*-test has been used for calculating statistical significance of experiments. Boxplots have been generated using BoxplotR.

ATP measurements

The protocol for ATP measurement adapted from SCENITH (Single Cell ENergetIc metabolism by profiling Translation inhibition) (82). For this, cells were plated at 1x 10^5 cells/ml, 0.2ml/well in 96-well plates for studying metabolism. Experimental duplicates/triplicates were performed in all conditions. After transfection or treatment, of cells, wells were treated during 45-60 minutes with DMSO, 2-Deoxy-D-Glucose (DG, final concentration 100mM), Oligomycin A (Oligo, final concentration 1μM), or a sequential combination of the drugs at the final concentrations. Cells without any cells were used as negative control. After treatment, ATP produced in cells was detected using Luminescent ATP detection assay kit as per manufacturer's protocol. Briefly, cells were lysed, substrate buffer added and luminescence was measured using BioTek SYNERGY HTX Reader at 540nm wavelength.

Supplementary figure legends

Figure S1. **ISG15 mediated post-translational modification of DRP1 affecting mitochondria.** (A) Reverse IP of samples corresponding to Figure1 panel A. ← indicates band of interest (ISG15). (B) Reverse IP of samples corresponding to Figure1 panel B. ← indicates band of interest (HERC5). (C) Graphs plot quantification of total mitochondrial volume (Z-stacks were taken with 0.15μm slices) and total mitochondria/cell. Data correspond to Figure1 panel E. ~150 cells from 3 independent experiments were analyzed. ** $p \leq 0.01$, *** $p \leq 0.001$ using unpaired 2-tailed Student's t-test. (D) Cells transfected with either control vector or HA-tagged ISG15 were treated with MitoTracker Green FM and imaged under live-cell conditions. Scale bar, 20μm. Overexpression was confirmed by immunoblotting against ISG15, β-TUBULIN was used as loading control. (E) Box plot showing quantification of mitochondrial length for the experiment described in panel D. ~75 cells from 3 independent experiments were analyzed. The central line and the plus (+) symbol in each box show the median and mean value, respectively. *** $p \leq 0.001$ using unpaired 2-tailed Student's t-test. (F) Cells treated with hIFNα1 similar to as described in Figure1 panel F were lysed and analysed for ISGylation of DRP1 by co-immunoprecipitation between ISG15 and DRP1. Note that presence of hIFNα1 induces enhanced ISGylation of DRP1. Further, partial rescue of endogenous DRP1 ISGylation was observed in mutant samples upon hIFNα1 treatment. ISG15 and VINCULIN served as loading controls. ◂ ISGylated endogenous DRP1, ◀ ISG15-modified mCherry-tagged DRP1, ← endogenous DRP1, ← mCherry-tagged DRP1. (G) Graphs plot quantification of total mitochondrial volume (Z-stacks were taken with 0.15μm slices) and total mitochondria/cell. Data correspond to Figure1 panel G. ~200 cells from 3 independent experiments were analyzed. ** $p \leq 0.01$, *** $p \leq 0.001$ using unpaired 2-tailed Student's t-test. (H) Cells treated with mock or ISG15 siRNAs were imaged under live-cell conditions with MitoTracker Green FM. Scale bar, 20μm. Knockdown efficiency was confirmed by immunoblotting against ISG15, β-TUBULIN was used as loading control. Box plot showing quantification of mitochondrial length for the experiment. ~75 cells from 3 independent experiments were analysed. The central line and the plus (+) symbol in each box show the median and mean value, respectively. *** $p \leq 0.001$ using unpaired 2-tailed Student's t-test. (I) Graphs plot quantification of total mitochondrial volume (Z-stacks were taken with 0.15μm slices) and total mitochondria/cell. Data correspond to panel H. ~70 cells from 3 independent experiments were analyzed. *** $p \leq 0.001$ using unpaired 2-tailed Student's t-test.

Figure S2. **CoV-2 PLpro alters mitochondrial morphology and function.** (A) Graphs plot quantification of total mitochondrial volume (Z-stacks were taken with 0.15µm slices) and total mitochondria/cell. Data correspond to Figure2 panel A. ~90 cells from 3 independent experiments were analyzed. ns, not significant ($p > 0.07$), *** $p \leq 0.001$ using unpaired 2-tailed Student's t-test. (B) Graph plots total ATP produced in A549 cells transfected with indicated constructs; a.u., arbitrary units. Data from 3 independent experiments were analyzed. ** $p \leq 0.01$ using unpaired 2-tailed Student's t-test. (C) Histogram plots percentage of ATP produced corresponding to glucose dependence, FAO (fatty acid oxidation) and AAO (amino acid oxidation) capacity, mitochondrial dependence and glycolytic capacity of cells transfected with indicated constructs. Data from 3 independent experiments were analyzed. ** $p \leq 0.01$ using unpaired 2-tailed Student's t-test. (D) HepG2 cells transfected with the indicated GFP-tagged constructs were imaged under live-cell conditions with MitoTracker Red FM. Immunoblot of lysates post imaging were probed against GFP and β-ACTIN to confirm transfection efficiency and similar loading. Scale bar, 5µm. (E) Box plot showing quantification of mitochondrial length for the experiment described in panel D. ~50 cells from 3 independent experiments were analyzed. The central line and the plus (+) symbol in each box show the median and mean values, respectively. *** $p \leq 0.001$ using unpaired 2-tailed Student's t-test. (F) Transfected cell lysates of the indicated GFP-tagged constructs were immunoprecipitated and immunoblotted. Note reduced ISGylation of MFN2 in CoV2 PLpro WT samples. The input levels of MFN2 and β-ACTIN in the total lysates served as loading controls. (G) Cells transfected and processed similarly as in Figure2 panel E were imaged in the slice 3D-SIM mode. White boxes show insets. White arrowheads mark TFAM puncta outside TOMM20 boundary. Immunoblot of lysates post imaging probed against GFP and β-ACTIN confirm transfection and similar loading. Scale bar, 10µm. (H) Cells transfected with indicated constructs were loaded with LIVE ORANGE mito and PicoGreen to visualise cristae and dsDNA, respectively, and imaged in the slice 3D-SIM live mode. Enlarged views of the areas within the white boxes shown (insets). White arrowheads mark PicoGreen puncta outside LIVE ORANGE mito boundary. Scale bar, 10µm.

Figure S3. **Cell line independent mtDNA release in presence CoV2 PLpro and activation of IFN-1 response pathway.** (A) HepG2 cells were transfected with indicated GFP-tagged constructs and immunostained with antibodies against TOMM20 and TFAM. Representative images were taken in Z-stacks (0.15µm slices), images show 3-D projections. Enlarged views

of the areas within the white boxes shown (insets). White arrowheads mark TFAM puncta outside TOMM20 boundary. Scale bar, 5µm. (B) Graph represents data from ~60 cells from 3 independent experiments. ns, not significant (p=0.2), ***p≤0.001 using unpaired 2-tailed Student's t-test. Error bars,±SEM. (C) Cytosolic and membrane fractions obtained from digitonin permeabilized A549 cells generated as in Figure2 panel H were immunoblotted against TFAM. HSP90 and RTN4 served as controls for cytosolic and membrane fractions, respectively. (D) Graph with results from panel C was obtained with results from 3 independent experiments. Note significantly reduced levels of TFAM in membrane fraction from samples with CoV-2 PLpro WT, more discernable in faint exposure of blots. ns, not significant (p=0.2), **p≤0.01 using unpaired 2-tailed Student's t-test. Error bars, ±SEM. (E) Digitonin permeabilized HepG2 cells fractionated into cytosolic and membrane fractions were immunoblotted against TFAM, HSP90 and RTN4. (F) Graph shows results of 3 independent experiments. ns, not significant (p=0.1), * p≤0.05 using unpaired 2-tailed Student's t-test. Error bars, ±SEM. (G) Cytosolic and mitochondrial fractions obtained from semi-permeabilized A549 cells were immunoblotted with TFAM antibody. VINCULIN and VDAC1 served as controls for cytosolic and mitochondrial fractions, respectively. (H) Graph shows results of 3 independent experiments. ns, not significant (p=0.3), * p≤0.05 using unpaired 2-tailed Student's t-test. Error bars, ±SEM. (I) DNA isolated from whole cell and cytosolic extracts was subjected to SYBR Green-based q-PCR to quantify nuclear (*GAPDH*) and mitochondrial (*mtND1*) DNA using specific primers. Plots show abundance of total (left) and cytosolic (right) cellular mtDNA. ns, not significant (p=0.09), * p≤0.05, ** p≤0.01 using unpaired 2-tailed Student's t-test. Error bars, ±SEM. (J) Total RNA isolated from A549 cells transfected with the indicated GFP-tagged constructs was subjected to quantitative reverse transcription-PCR (qRT-PCR) performed using SYBR Green and primers against *IFNB1*, *IFIT1*, *IRF7*, *ISG15* and *GAPDH*. Samples were present in triplicate. $2^{-\Delta\Delta Ct}$ values for each gene were plotted. Graphs show results from 3 independent experiments. ns, not significant (p>0.1), * p≤0.05, ** p≤0.01, *** p≤0.001 using unpaired 2-tailed Student's t-test. Error bars, ±SEM.

Figure S4. **HERC5 knockdown affects mitochondrial dynamics and mtDNA release.** (A) Cells treated with mock or HERC5 siRNA pools were imaged under live-cell conditions with MitoTracker Green FM. Scale bar, 20µm. Knockdown efficiency was confirmed by immunoblotting against HERC5, VINCULIN was used as loading control. (B) Box plot

showing quantification of mitochondrial length for the experiment. ~50 cells from 3 independent experiments were analyzed. The central line and the plus (+) symbol in each box show the median and mean value, respectively. *** $p \leq 0.001$ using unpaired 2-tailed Student's t-test. (C) Graphs plot quantification of total mitochondrial volume (Z-stacks were taken with 0.15μm slices) and total mitochondria/cell. Data correspond to panel A. ~50 cells from 3 independent experiments were analyzed. ** $p \leq 0.01$, *** $p \leq 0.001$ using unpaired 2-tailed Student's t-test. (D) Cells treated with indicated siRNA pools and immunostained with antibodies against TOMM20 and TFAM were imaged. Z-stacks (0.15μm slices) were taken. Images show 3-D projections. Insets indicated (white boxes). White arrowheads mark TFAM puncta outside TOMM20 boundary. Scale bar, 20μm. Graph represents data from ~55 cells from 3 independent experiments. *** $p \leq 0.001$, using unpaired 2-tailed Student's t-test. Error bars, ±SEM. Imunoblot of lysates post-imaging confirm HERC5 depletion. Lysates generated were immunoblotted against DRP1and ISG15; VINCULIN was used as loading control. Graphs show the changes in the expression of DRP1 and ISG15. Data represents 3 independent experiments. *** $p \leq 0.001$ using unpaired 2-tailed Student's t-test. Error bars, ±SEM.

Figure S5. **Post-translational modifications of DRP1, PARKIN and MITOL.** (A) A549 cell lysates generated similarly as in Figure 4 panel B were verified for DRP1 ubiquitylation by endogenous Ub. Lysates were co-immunoprecipitated and probed against DRP1 and Ub. Note, increased DRP1 ubiquitylation in CoV2 PLpro WT samples. DRP1 and VINCULIN levels in the total lysates served as loading controls. ← marks the prominent ubiquitylated form of DRP1. (B) Cells co-transfected with CoV2 PLpro WT along with DRP1 or its mutant were co-immnunoprecipitated similar to Figure 4 panel E. Note decrease in ubiquitylated DRP1 detected in presence of the K532R mutant when compared with the mCherry-tagged wild type construct. VINCULIN served as loading control. ⬅ endogenous DRP1, ← mCherry-tagged DRP1. (C) A549 cells were transfected with indicated GFP-tagged constructs were immunoblotted with anti-DRP1 antibody. Note unchanged DRP1 levels upon PARKIN overexpression. Input levels of GFP and β-TUBULIN served as loading controls. ← Cytosolic GFP, ← CoV2 PLpro GFP, ⬅ PARKIN GFP. (D) Cells were similarly transfected as panel C, except MITOL GFP was used instead of PARKIN GFP. Input levels of GFP and β-TUBULIN served as loading controls. ← Cytosolic GFP, ← CoV2 PLpro GFP, ⬅ MITOL GFP. (E) A549 cells were transfected with indicated constructs along with

PARKIN GFP and analysed for auto-ubiquitylation of the E3 ligase. Lysates were co-immunoprecipitated and probed against GFP and endogenous Ub. Note, decreased PARKIN GFP ubiquitylation in CoV2 PLpro WT samples. PARKIN and VINCULIN levels in the total lysates served as loading controls. (F) Cells were transfected with indicated constructs analysed for auto-ubiquitylation of endogenous MITOL. Lysates were co-immunoprecipitated and probed against MITOL and Ub. Note, decreased MITOL ubiquitylation in CoV2 PLpro WT samples; MITOL and VINCULIN levels in the total lysates served as loading controls. (G) A549 cell lysates were co-immunoprecipitated against ISG15 and MITOL. Note a shift in band size (~15KDa) detected between input and immunoprecipitated samples, indicating ISGlyated from of MITOL. The proportion of lysate loaded as input and immunoprecipitate denoted in brackets by 'X'. (H) Cells transfected with indicated GFP-tagged constructs were immunoblotted against PARKIN, MITOL, TRIM25 and HERC5 antibodies. Note that the E3 ligase levels remain unchanged across samples. VINCULIN served as loading control. Graphs plot expression levels of the indicated proteins. Data represents 3 independent experiments. Error bars, ±SEM. ns, not significant ($p>0.09$), using unpaired 2-tailed Student's t-test.

Figure S6. **TRIM25 regulates DRP1 stability and mitochondrial dynamics.** (A) A549 cells were transfected with control vector or TRIM25 GFP, and were either left untreated or and treated with cycloheximide (Chx, 100 μg/ml) for indicated time periods. DRP1 protein levels in cell lysates was analysed. β-ACTIN was used as loading control. ⬅ endogenous TRIM25, ← GFP-tagged TRIM25. Graph shows quantification of DRP1 levels over time. Data represents 3 independent experiments. ns, not significant ($p=0.12$), $^{*}p\leq0.033$, $^{**}p\leq0.002$, $^{***}p\leq0.001$ using 2way ANOVA with Bonferroni corrections. Error bars, ±SEM. (B) Reverse IP of samples corresponding to Figure 4 panel H. ← marks the band corresponding to DRP1. (C) Cell treated with mock or TRIM25 siRNA pools were co-immunoprecipitated against Ub and DRP1. Note reduced ubiquitylated DRP1 levels in TRIM25 knockdown samples and increased DRP1 levels in cell lysates of the same. TRIM25 levels in the lysates confirm knockdown efficiency; VINCULIN served as loading control. (D) Cells treated with mock or TRIM25 siRNA pools were imaged under live-cell conditions with MitoTracker Green FM. Scale bar, 20μm. Knockdown efficiency was confirmed by immunoblotting against TRIM25; VINCULIN was used as loading control. (E) Box plot showing quantification of mitochondrial length for the experiment in panel C. ~40 cells from

3 independent experiments were analyzed. The central line and the plus (+) symbol in each box show the median and mean value, respectively. *** p≤0.001 using unpaired 2-tailed Student's t-test. (F) Graphs plot quantification of total mitochondrial volume (Z-stacks were taken with 0.15μm slices) and total mitochondria/cell. Data correspond to panel C. ~40 cells from 3 independent experiments were analyzed. *** p≤0.001 using unpaired 2-tailed Student's t-test.

Figure S7. **Effect of DRP1^{K532R} on various post-translational modifications of DRP1.** (A) Cells transfected with indicated mCherry-tagged constructs were immunoblotted with anti-phospho-DRP1 (S616) antibody. Note expression of DRP1^{K532R} did not influence DRP1 phosphorylation at S616. β-TUBULIN served as loading control. ⬅ endogenous DRP1, ← mCherry-tagged DRP1. (B) Cell lysates as generated in panel A were immunoblotted against phospho-DRP1 (S637). Note expression of DRP1^{K532R} did not affect DRP1 phosphorylation at S637. β-TUBULIN served as loading control. ⬅ endogenous DRP1, ← mCherry-tagged DRP1. (C) A549 cells were transfected with indicated mCherry-tagged constructs and co-immunoprecipitated against SUMO1 and DRP1. Note similar SUMOylation levels across all samples. Point mutation at K532 residue did not alter the extent of this modification. VINCULIN served as loading control. ⬅ endogenous DRP1, ← mCherry- tagged DRP1. (D) Cells were transfected with indicated mCherry-tagged constructs, immunoprecipitated against MiD49 and immunoblotted with DRP1. Note compromised interaction between MiD49 and DRP1 in presence of K532R mutation. MiD49 levels were verified in input samples; β-ACTIN was used as loading control. (E) Cells were similarly transfected and processed as panel H, except that co-immunoprecipitation was verified between MFF and DRP1. Note compromised interaction between MFF and DRP1 in presence of K532R mutation. MFF levels were checked lysates; β-ACTIN served as loading control. (F) Cells transfected with indicated mCherry-tagged constructs were immunoblotted with anti- phospho-DRP1 (S616) antibody. Note expression of DRP1^{S616D} increased DRP1 phosphorylation at S616. VINCULIN served as loading control. ⬅ endogenous DRP1, ← mCherry-tagged DRP1. (G) Cells transfected with indicated mCherry-tagged constructs were immunoblotted against phospho-DRP1 (S616). Note expression of DRP1^{K532RS616D} enhanced DRP1 phosphorylation at S616. VINCULIN served as loading control. ⬅ endogenous DRP1, ← mCherry-tagged DRP1.

Figure S8. **Altered mitochondrial function in Alzheimer's disease.** (A) Graph plots total ATP produced in SHSY5Y cells transfected with indicated constructs and treated with DMSO or 0.5μM Aβ for 24h; a.u., arbitrary units. Data from 3 independent experiments were analyzed. ** $p \leq 0.01$ using unpaired 2-tailed Student's t-test. (B) Histogram plots percentage of ATP produced corresponding to glucose dependence, FAO (fatty acid oxidation) and AAO (amino acid oxidation) capacity, mitochondrial dependence and glycolytic capacity of cells analysed in panel A. Data from 3 independent experiments were analyzed. * $p \leq 0.05$, *** $p \leq 0.001$ using unpaired 2-tailed Student's t-test. (C) Graph plots total ATP produced in SHSY5Y cells treated with DMSO or 1μM Aβ for 24h; a.u., arbitrary units. Data from 3 independent experiments were analyzed. *** $p \leq 0.001$ using unpaired 2-tailed Student's t-test. (D) Histogram similar to panel B plotted with data corresponding to panel C. ** $p \leq 0.01$, *** $p \leq 0.001$ using unpaired 2-tailed Student's t-test.

Figure S1

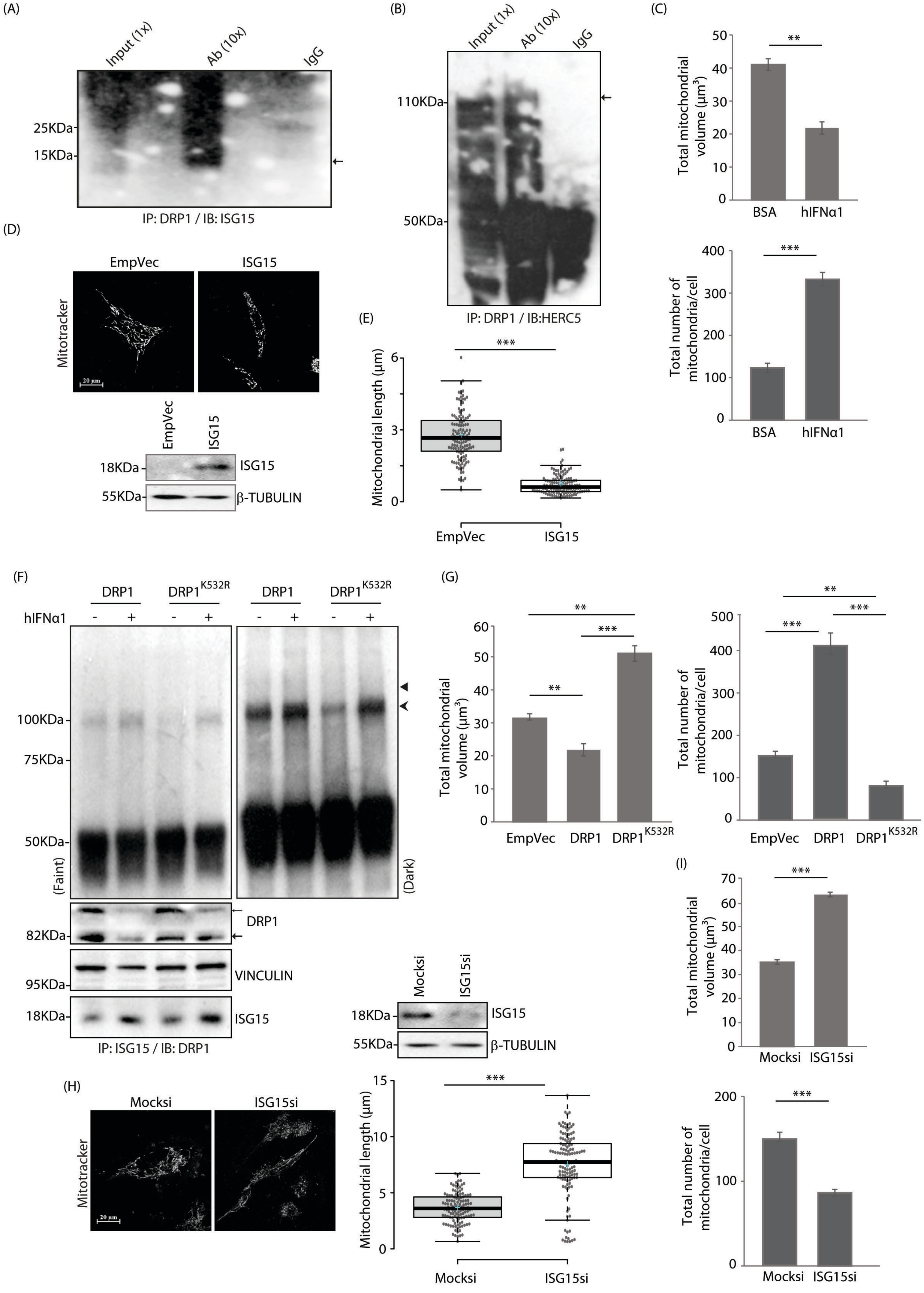

(A)
Input (1x)
Ab (10x)
IgG
25KDa
15KDa
IP: DRP1 / IB: ISG15
(B)
Input (1x)
Ab (10x)
IgG
110KDa
50KDa
IP: DRP1 / IB:HERC5
(C)
Total mitochondrial volume (μm3)
BSA
hIFNα1
Total number of mitochondria/cell
(D)
EmpVec
ISG15
Mitotracker
18KDa
55KDa
β-TUBULIN
(E)
Mitochondrial length (μm)
(F)
DRP1
DRP1K532R
hIFNα1
100KDa
75KDa
50KDa
(Faint)
(Dark)
82KDa
95KDa
18KDa
VINCULIN
IP: ISG15 / IB: DRP1
(G)
EmpVec
Mocksi
ISG15si
(H)
(I)

Figure S2

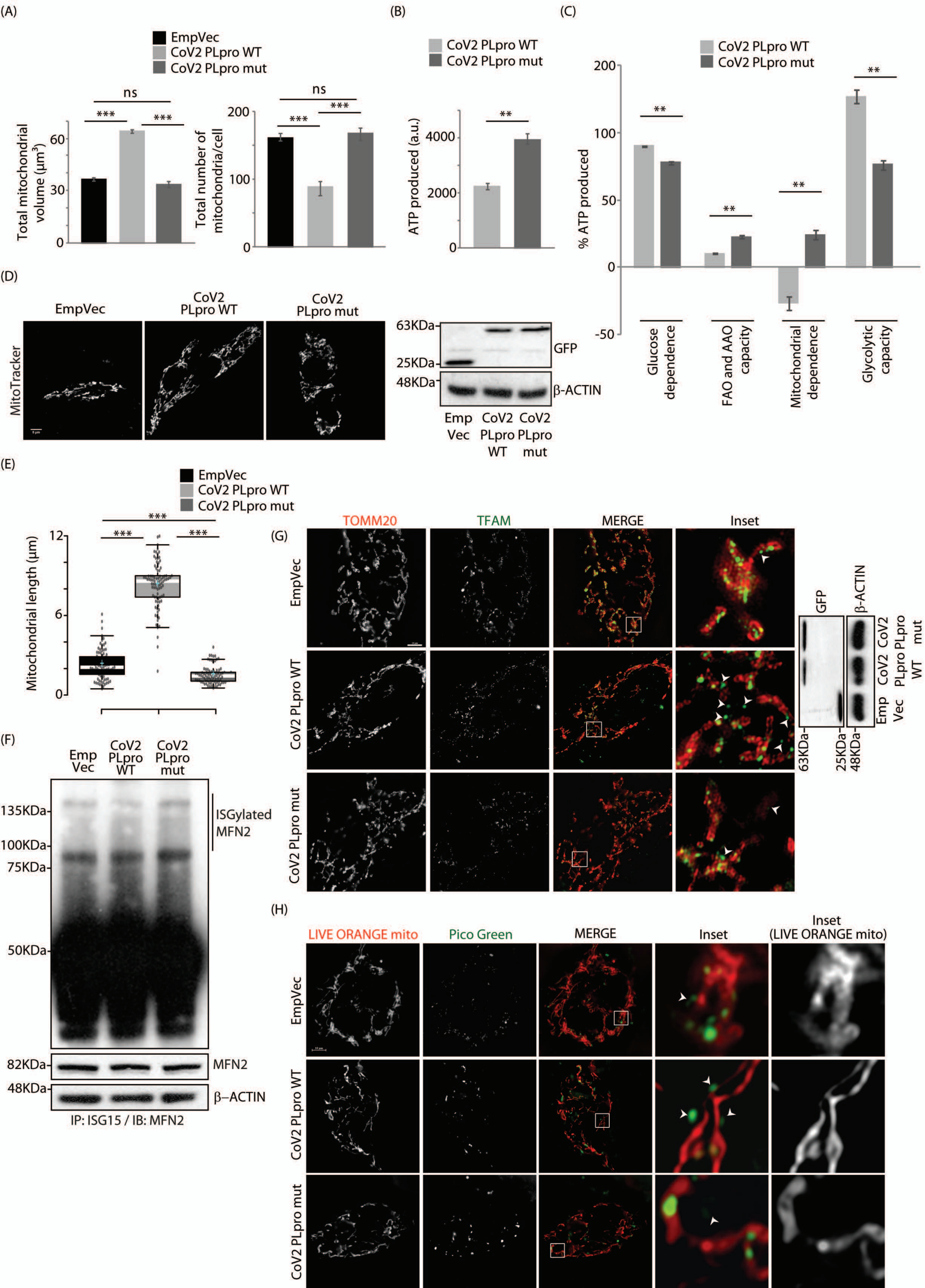

(A)
EmpVec
CoV2 PLpro WT
CoV2 PLpro mut
Total mitochondrial volume (μm³)
Total number of mitochondria/cell
ns
***
(B)
ATP produced (a.u.)
**
(C)
% ATP produced
Glucose dependence
FAO and AAO capacity
Mitochondrial dependence
Glycolytic capacity
(D)
MitoTracker
63KDa
25KDa
48KDa
GFP
β-ACTIN
Emp Vec
CoV2 PLpro WT
CoV2 PLpro mut
(E)
Mitochondrial length (μm)
(F)
135KDa
100KDa
75KDa
50KDa
82KDa
ISGylated MFN2
MFN2
IP: ISG15 / IB: MFN2
(G)
TOMM20
TFAM
MERGE
Inset
(H)
LIVE ORANGE mito
Pico Green
Inset (LIVE ORANGE mito)

Figure S3

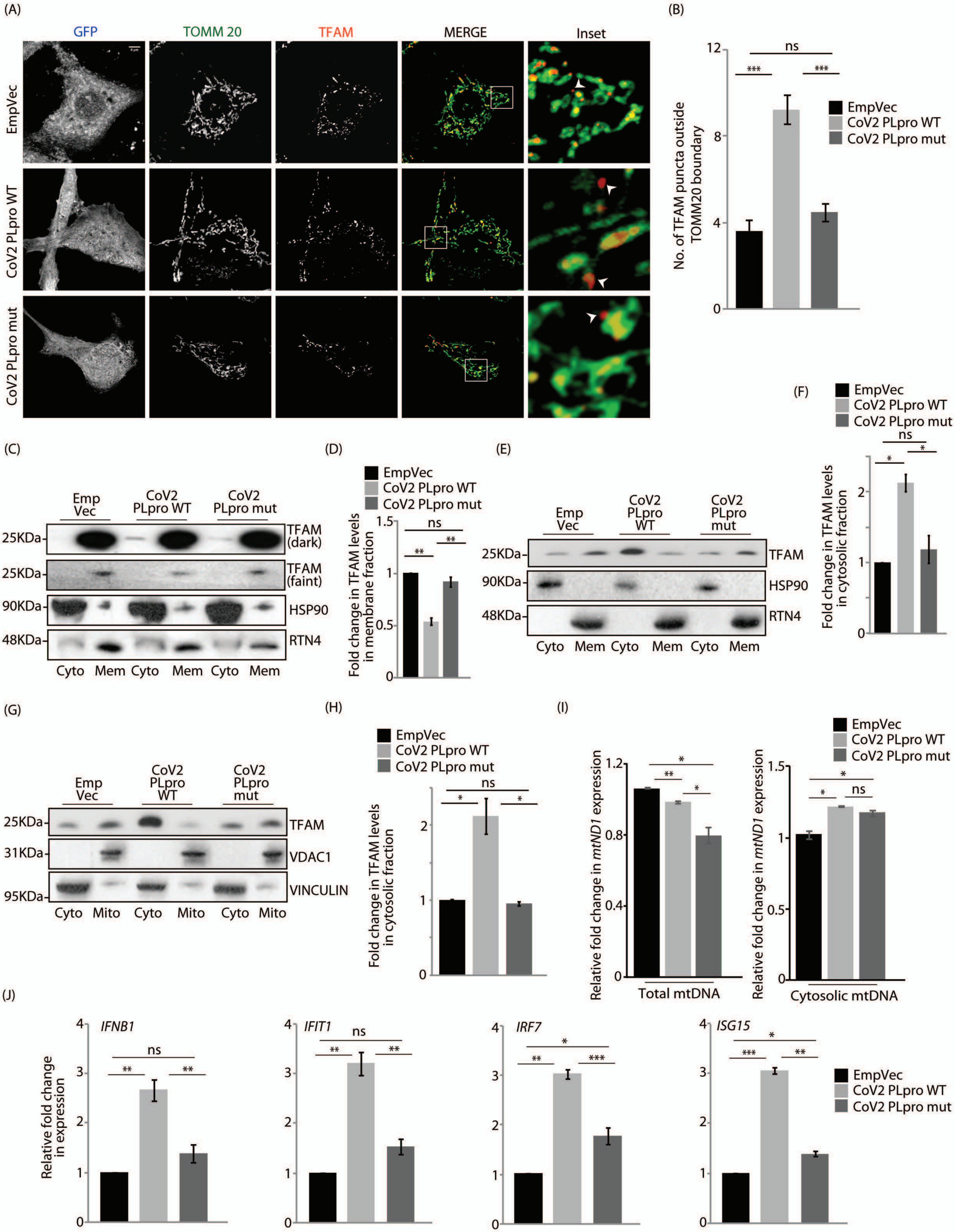
(A)
GFP
TOMM 20
TFAM
MERGE
Inset
EmpVec
CoV2 PLpro WT
CoV2 PLpro mut
(B)
No. of TFAM puncta outside TOMM20 boundary
EmpVec
CoV2 PLpro WT
CoV2 PLpro mut
(C)
Emp Vec
CoV2 PLpro WT
CoV2 PLpro mut
TFAM (dark)
TFAM (faint)
HSP90
RTN4
Cyto Mem Cyto Mem Cyto Mem
(D)
Fold change in TFAM levels in membrane fraction
(E)
TFAM
HSP90
RTN4
(F)
Fold change in TFAM levels in cytosolic fraction
(G)
TFAM
VDAC1
VINCULIN
Cyto Mito Cyto Mito Cyto Mito
(H)
(I)
Relative fold change in mtND1 expression
Total mtDNA
Cytosolic mtDNA
(J)
IFNB1
IFIT1
IRF7
ISG15
Relative fold change in expression

Figure S4

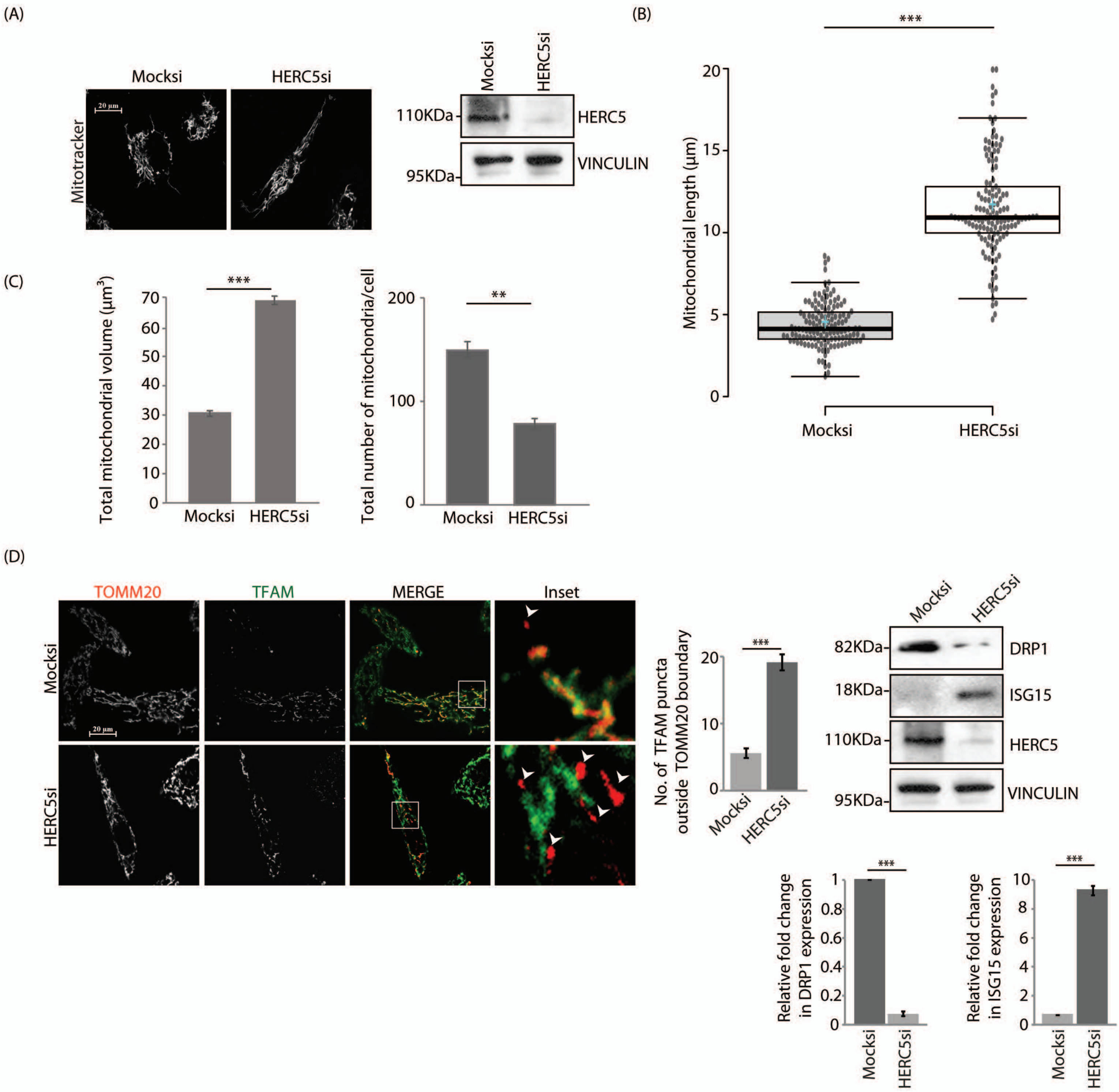

(A)
Mocksi
HERC5si
Mitotracker
20 µm
Mocksi
HERC5si
110KDa
HERC5
95KDa
VINCULIN
(B)
***
Mitochondrial length (µm)
0
5
10
15
20
Mocksi
HERC5si
(C)
***
Total mitochondrial volume (µm³)
0
10
20
30
40
50
60
70
Mocksi
HERC5si
**
Total number of mitochondria/cell
0
100
200
Mocksi
HERC5si
(D)
TOMM20
TFAM
MERGE
Inset
Mocksi
HERC5si
20 µm
No. of TFAM puncta outside TOMM20 boundary
***
0
10
20
Mocksi
HERC5si
Mocksi
HERC5si
82KDa
DRP1
18KDa
ISG15
110KDa
HERC5
95KDa
VINCULIN
Relative fold change in DRP1 expression
***
0
0.2
0.4
0.6
0.8
1
Mocksi
HERC5si
Relative fold change in ISG15 expression
***
0
2
4
6
8
10
Mocksi
HERC5si

Figure S5

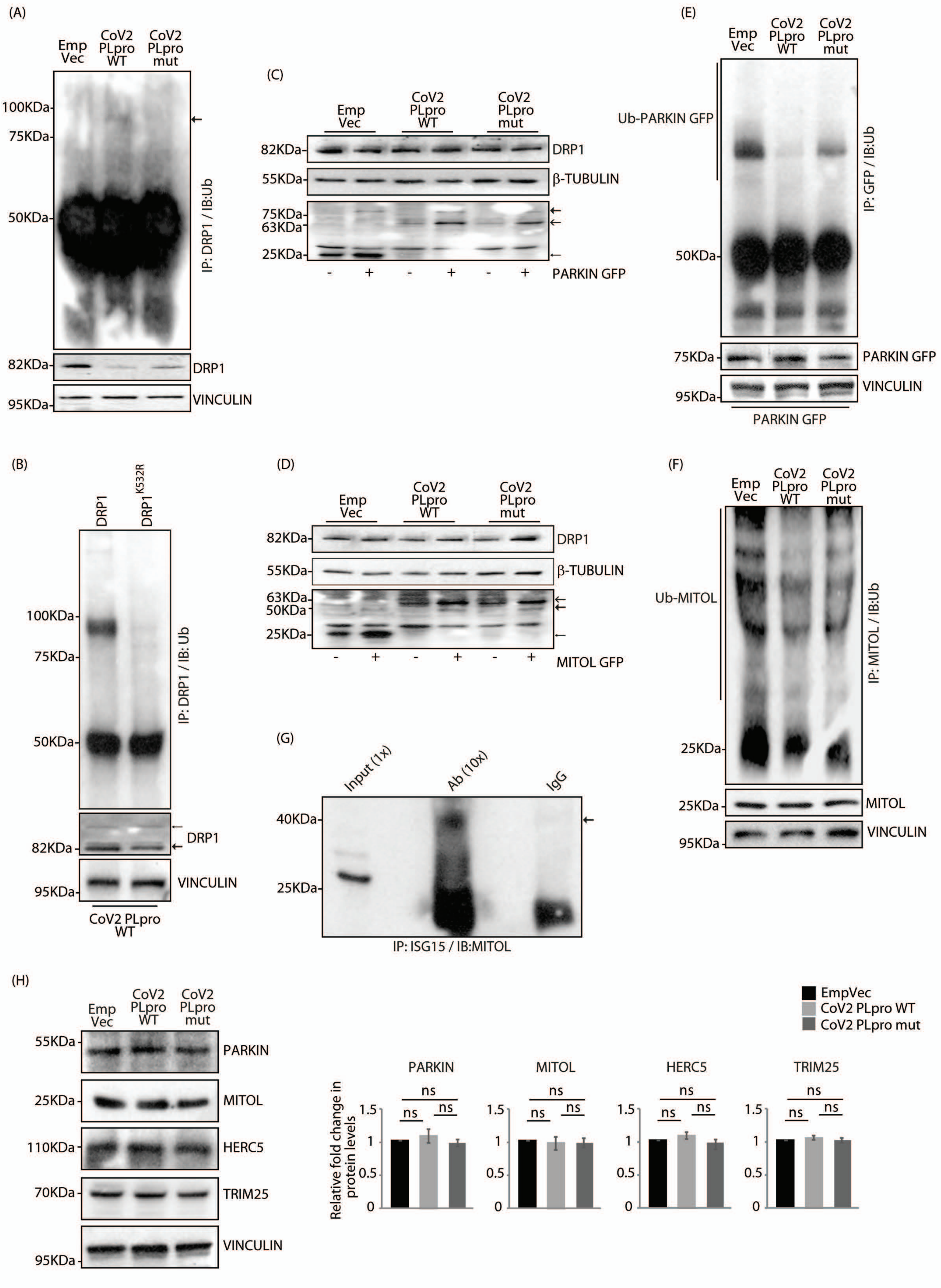

(A)
Emp Vec
CoV2 PLpro WT
CoV2 PLpro mut
100KDa
75KDa
50KDa
IP: DRP1 / IB:Ub
82KDa
DRP1
95KDa
VINCULIN
(B)
DRP1
DRP1 K532R
100KDa
75KDa
50KDa
IP: DRP1 / IB: Ub
82KDa
DRP1
95KDa
VINCULIN
CoV2 PLpro WT
(C)
Emp Vec
CoV2 PLpro WT
CoV2 PLpro mut
82KDa
DRP1
55KDa
β-TUBULIN
75KDa
63KDa
25KDa
PARKIN GFP
(D)
Emp Vec
CoV2 PLpro WT
CoV2 PLpro mut
82KDa
DRP1
55KDa
β-TUBULIN
63KDa
50KDa
25KDa
MITOL GFP
(E)
Emp Vec
CoV2 PLpro WT
CoV2 PLpro mut
Ub-PARKIN GFP
IP: GFP / IB:Ub
50KDa
75KDa
PARKIN GFP
95KDa
VINCULIN
PARKIN GFP
(F)
Emp Vec
CoV2 PLpro WT
CoV2 PLpro mut
Ub-MITOL
IP: MITOL / IB:Ub
25KDa
25KDa
MITOL
95KDa
VINCULIN
(G)
Input (1x)
Ab (10x)
IgG
40KDa
25KDa
IP: ISG15 / IB:MITOL
(H)
Emp Vec
CoV2 PLpro WT
CoV2 PLpro mut
55KDa
PARKIN
25KDa
MITOL
110KDa
HERC5
70KDa
TRIM25
95KDa
VINCULIN
EmpVec
CoV2 PLpro WT
CoV2 PLpro mut
PARKIN
MITOL
HERC5
TRIM25
ns
Relative fold change in protein levels
1.5
1
0.5
0

Figure S6

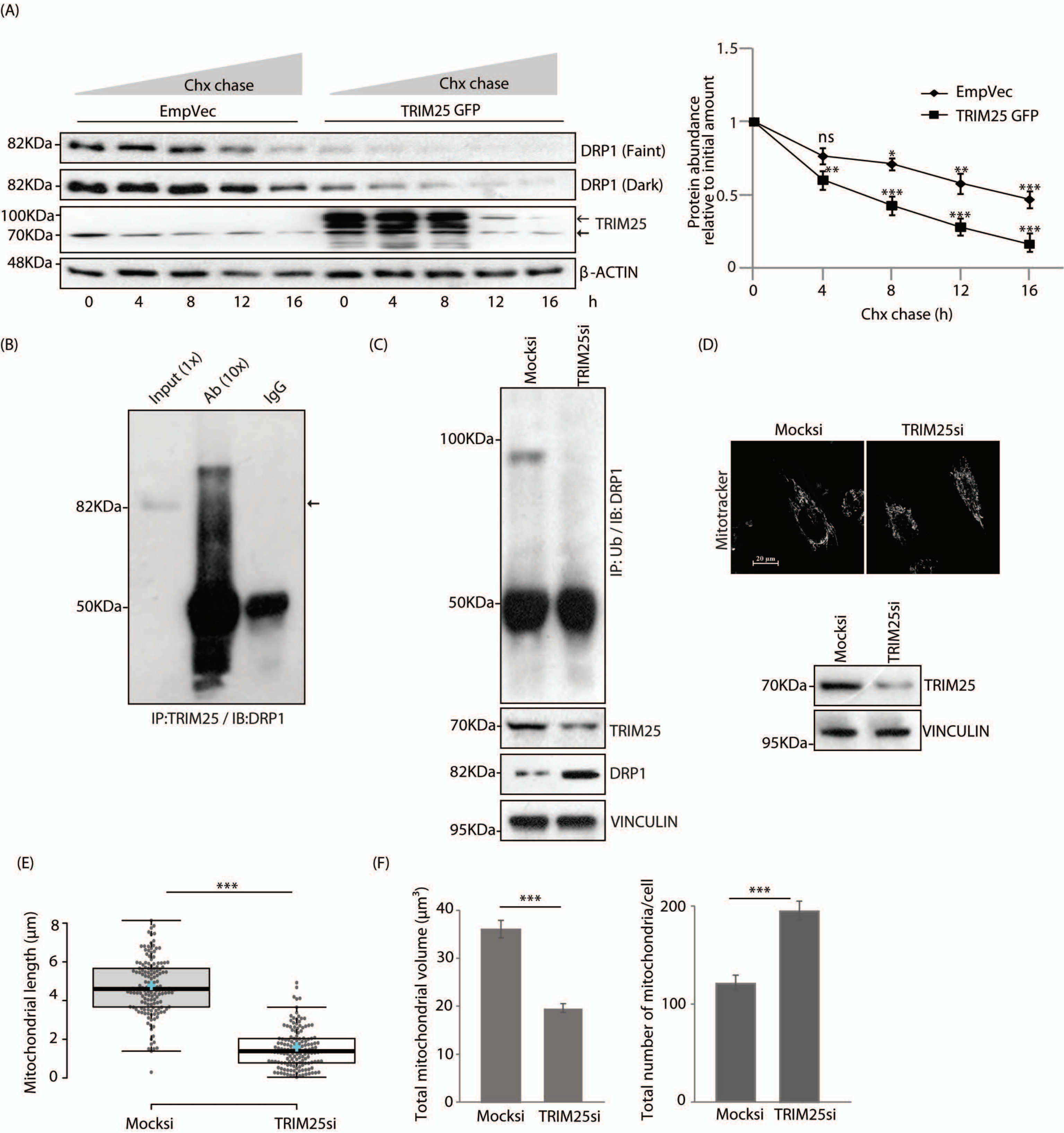
(A)
Chx chase
Chx chase
EmpVec
TRIM25 GFP
82KDa
82KDa
100KDa
70KDa
48KDa
DRP1 (Faint)
DRP1 (Dark)
TRIM25
β-ACTIN
0 4 8 12 16 0 4 8 12 16 h
Protein abundance relative to initial amount
EmpVec
TRIM25 GFP
Chx chase (h)
(B)
Input (1x)
Ab (10x)
IgG
82KDa
50KDa
IP:TRIM25 / IB:DRP1
(C)
Mocksi
TRIM25si
100KDa
50KDa
IP: Ub / IB: DRP1
70KDa
TRIM25
82KDa
DRP1
95KDa
VINCULIN
(D)
Mocksi
TRIM25si
Mitotracker
20 μm
Mocksi
TRIM25si
70KDa
TRIM25
95KDa
VINCULIN
(E)
Mitochondrial length (μm)
Mocksi
TRIM25si
(F)
Total mitochondrial volume (μm³)
Mocksi
TRIM25si
Total number of mitochondria/cell
Mocksi
TRIM25si

Figure S7

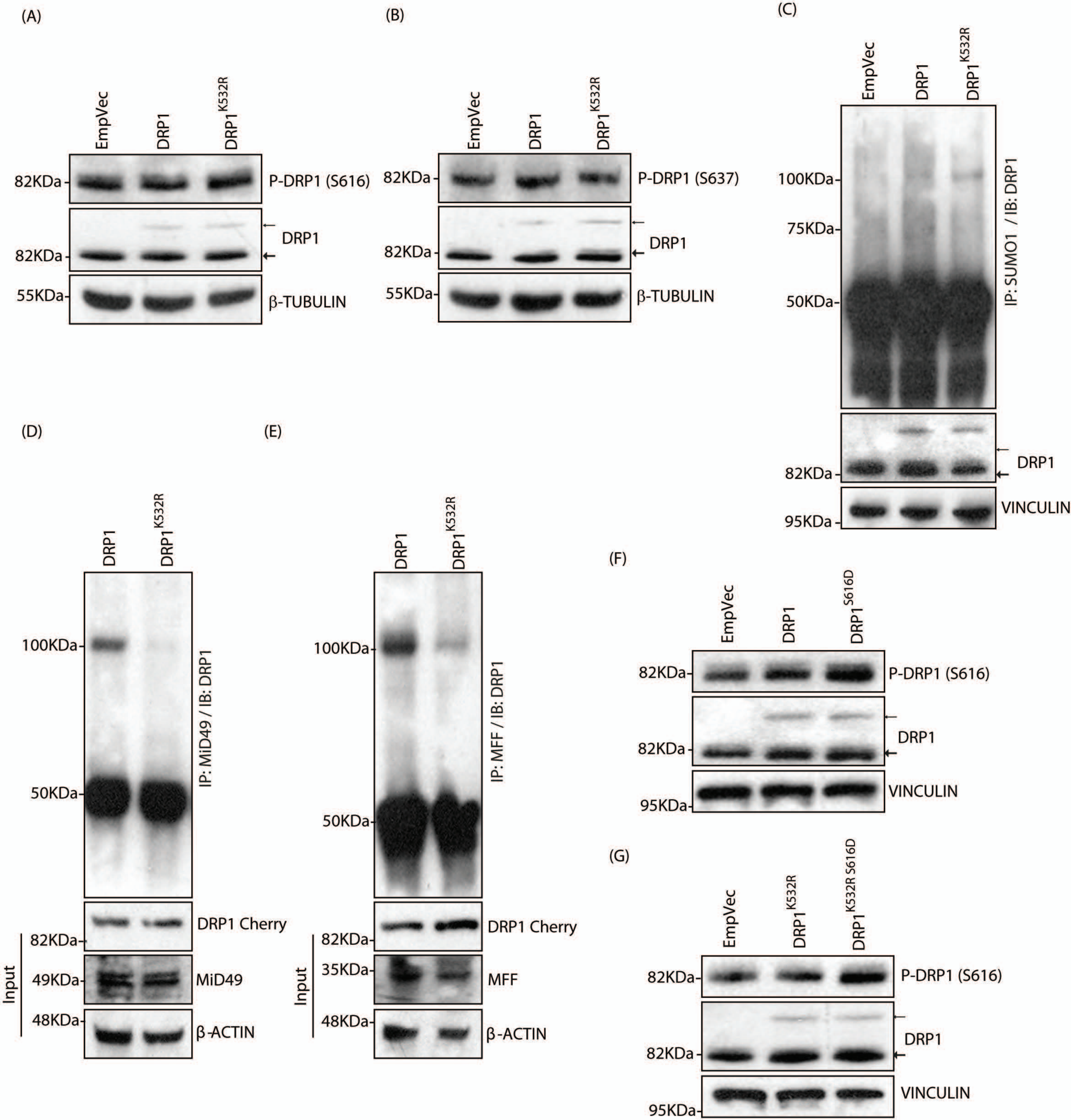
(A)
EmpVec
DRP1
DRP1K532R
82KDa
P-DRP1 (S616)
DRP1
82KDa
55KDa
β-TUBULIN
(B)
EmpVec
DRP1
DRP1K532R
82KDa
P-DRP1 (S637)
DRP1
82KDa
55KDa
β-TUBULIN
(C)
EmpVec
DRP1
DRP1K532R
100KDa
75KDa
50KDa
IP: SUMO1 / IB: DRP1
DRP1
82KDa
VINCULIN
95KDa
(D)
DRP1
DRP1K532R
100KDa
50KDa
IP: MiD49 / IB: DRP1
DRP1 Cherry
82KDa
49KDa
MiD49
48KDa
β-ACTIN
Input
(E)
DRP1
DRP1K532R
100KDa
50KDa
IP: MFF / IB: DRP1
DRP1 Cherry
82KDa
35KDa
MFF
48KDa
β-ACTIN
Input
(F)
EmpVec
DRP1
DRP1S616D
82KDa
P-DRP1 (S616)
DRP1
82KDa
VINCULIN
95KDa
(G)
EmpVec
DRP1K532R
DRP1K532R S616D
82KDa
P-DRP1 (S616)
DRP1
82KDa
VINCULIN
95KDa

Figure S8

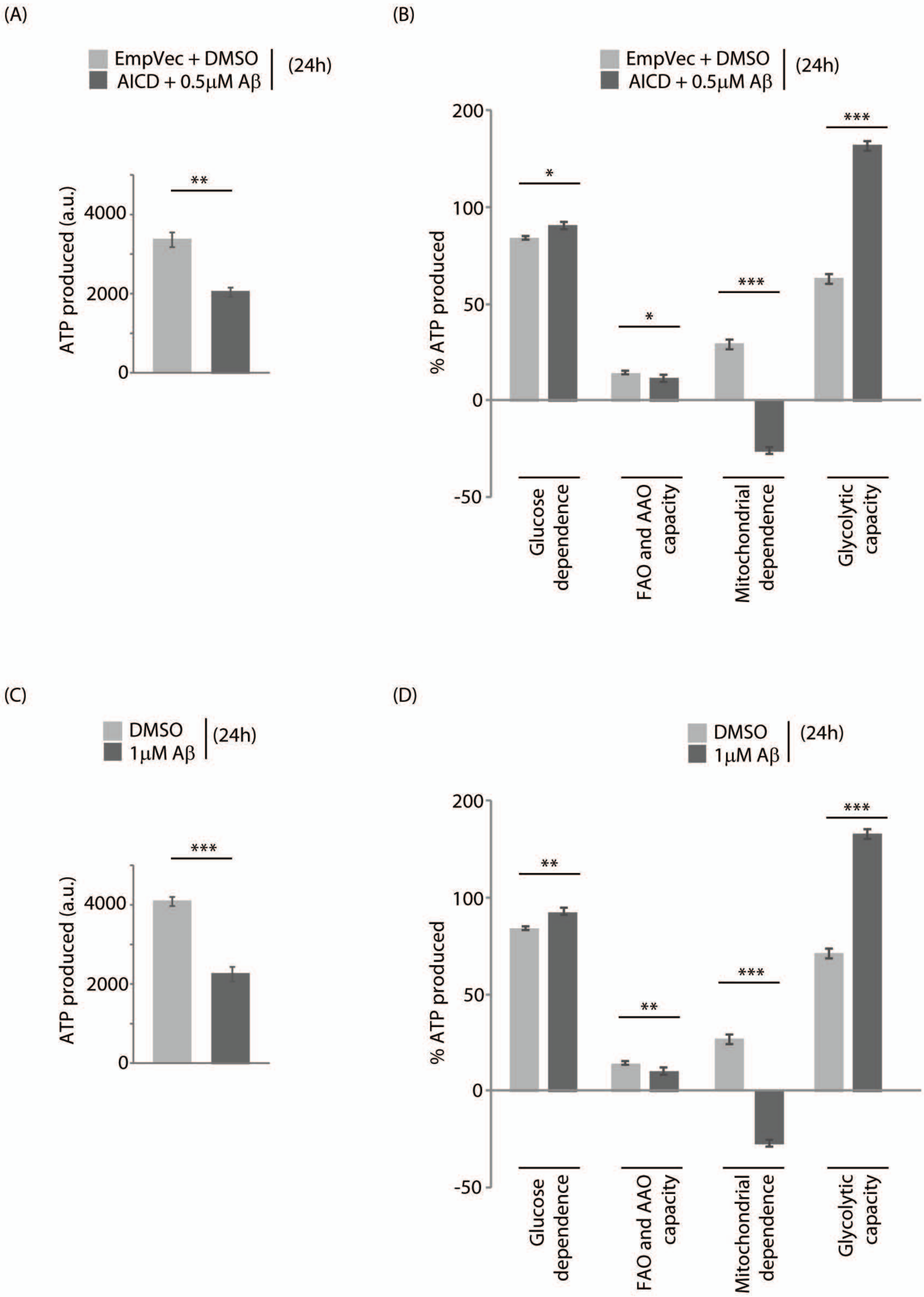
(A)
EmpVec + DMSO
AICD + 0.5μM Aβ
(24h)
ATP produced (a.u.)
4000
2000
0
**
(B)
EmpVec + DMSO
AICD + 0.5μM Aβ
(24h)
% ATP produced
200
100
50
0
-50
*
*
***
***
Glucose dependence
FAO and AAO capacity
Mitochondrial dependence
Glycolytic capacity
(C)
DMSO
1μM Aβ
(24h)
ATP produced (a.u.)
4000
2000
0
***
(D)
DMSO
1μM Aβ
(24h)
% ATP produced
200
100
50
0
-50
**
**
***
***
Glucose dependence
FAO and AAO capacity
Mitochondrial dependence
Glycolytic capacity
