# Supplementary material for: ISGylation of DRP1 closely balances other post-translational modifications to mediate mitochondrial fission
Source: Cell Death Dis. 2024 Mar 2;15(3):184. doi: 10.1038/s41419-024-06543-7 (PMC10908869; doi:10.1038/s41419-024-06543-7)
Supplement: Supplementary file 3 — Original Data File [file 41419_2024_6543_MOESM3_ESM.pdf]

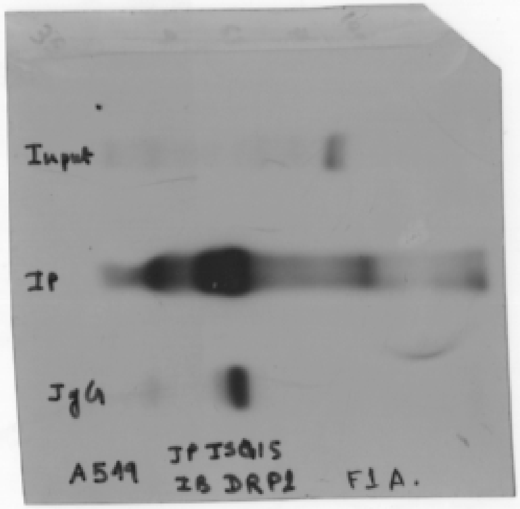
Input
IP
IgG
A549
IP ISG15
IB DRP1
F1A.

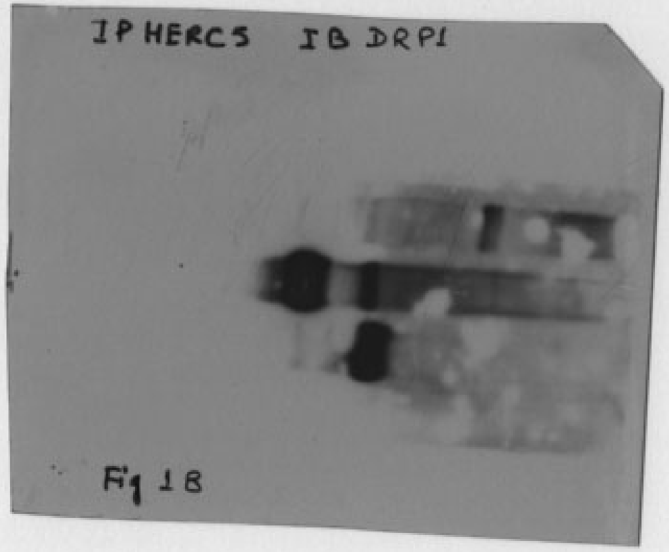
IP HERC5 IB DRP1
Fig 1B

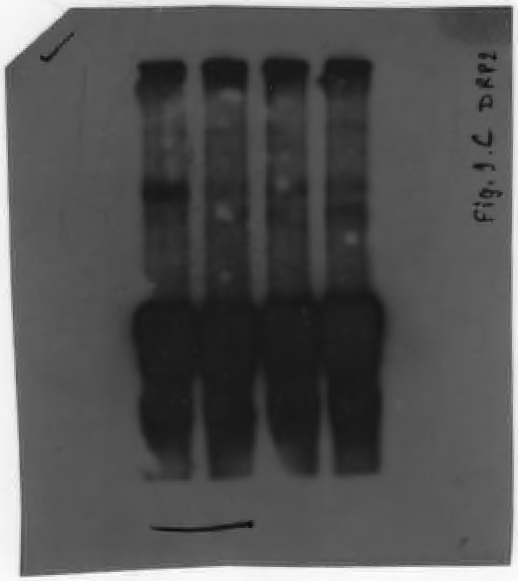
Fig. 1.C DRP2

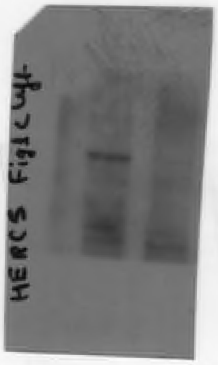
HERC5 Fig1C cyt

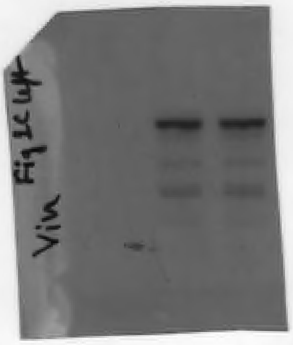
Vin Fig 1C cyt

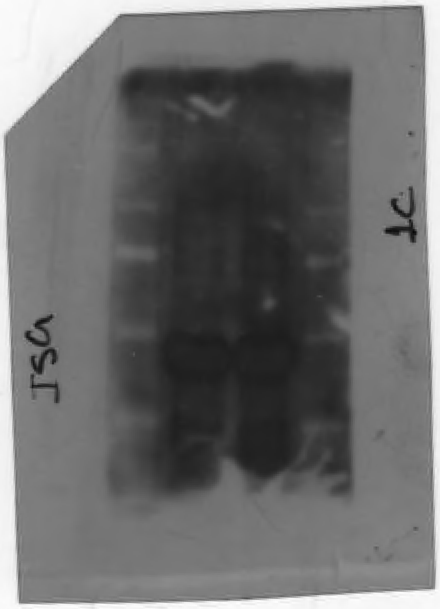

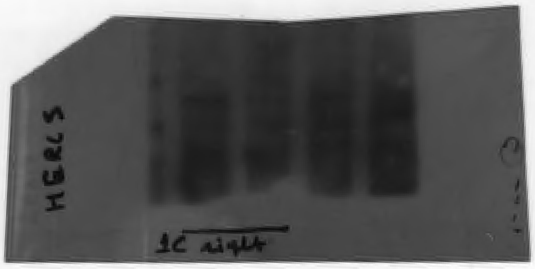
1C right

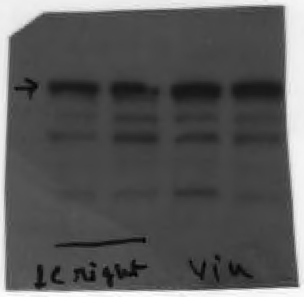
1C right
vin

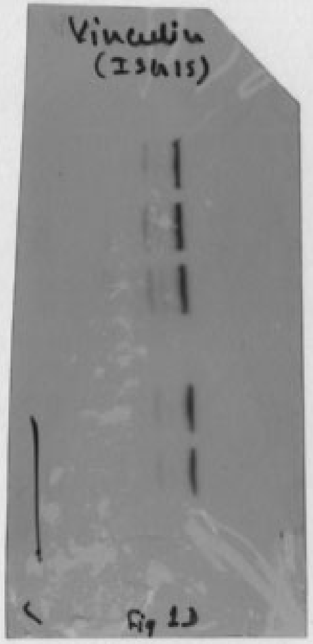
Vinculin
(ISG15)
Fig 1D

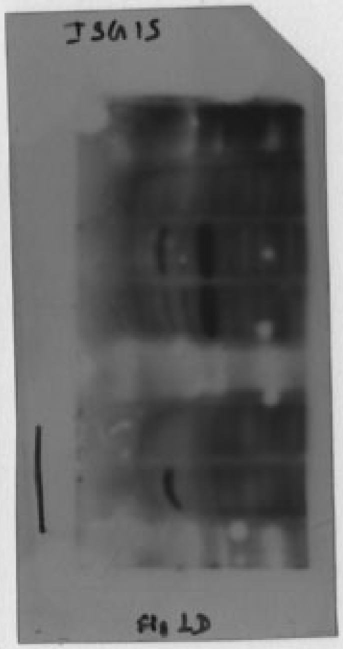
ISG15
Fig 1D

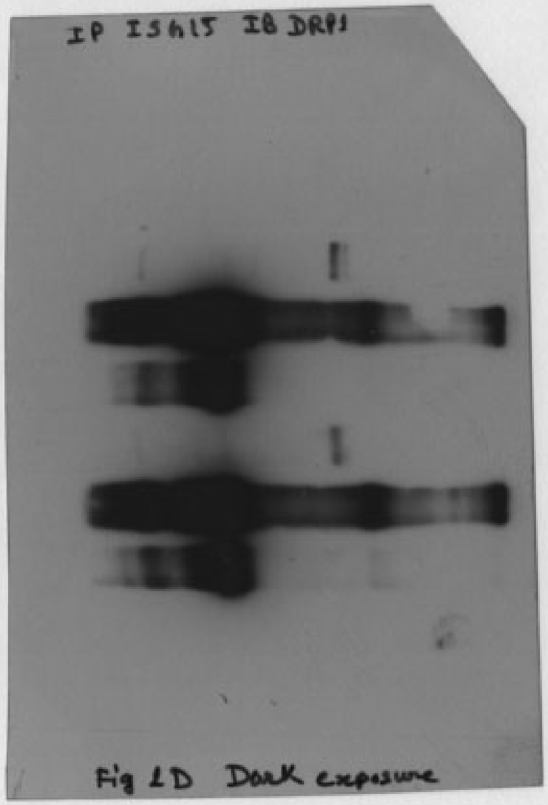
IP ISG15 IB DRP1
Fig 1D Dark exposure

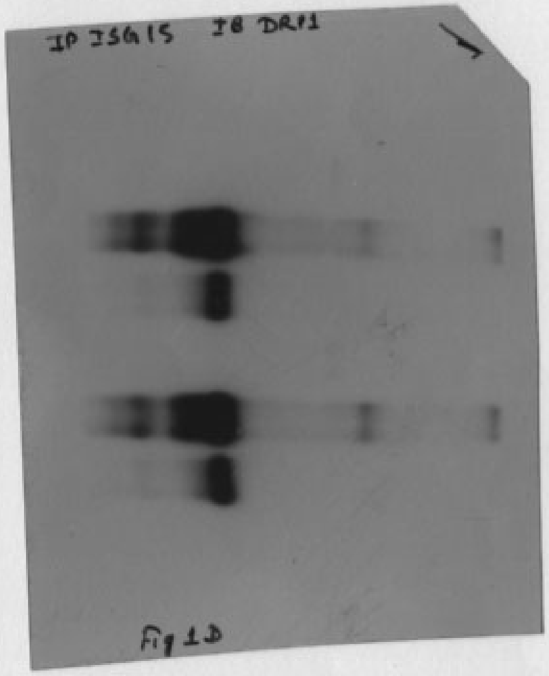
IP ISG15 IB DRP1
Fig 1D

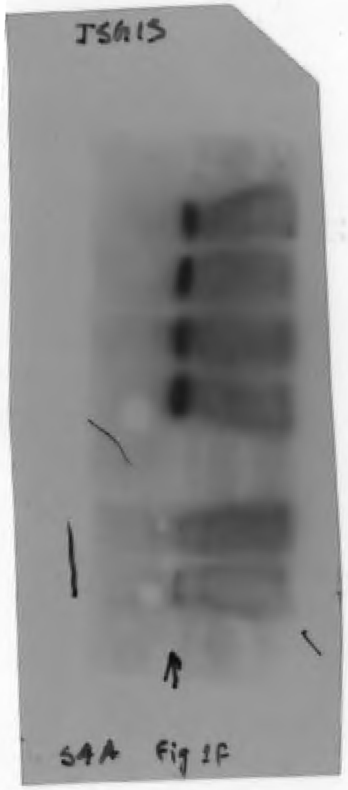
ISG15
S4A Fig 1F

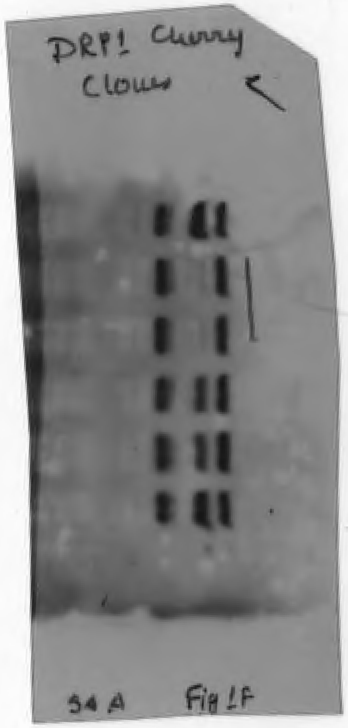
DRP1 Cherry
Clones
S4 A Fig 1F

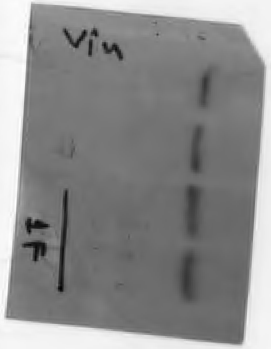
Vin
1F

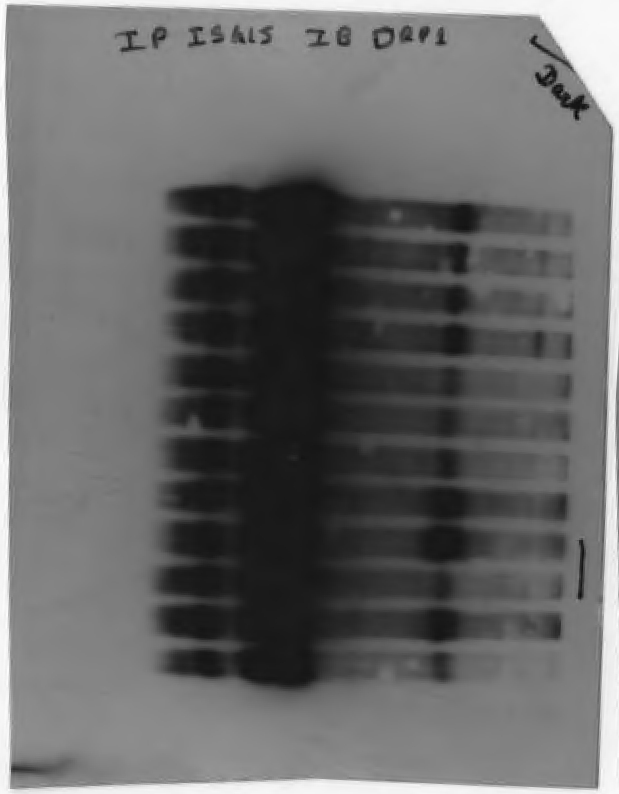
IP ISG15 IB DRP1
Dark

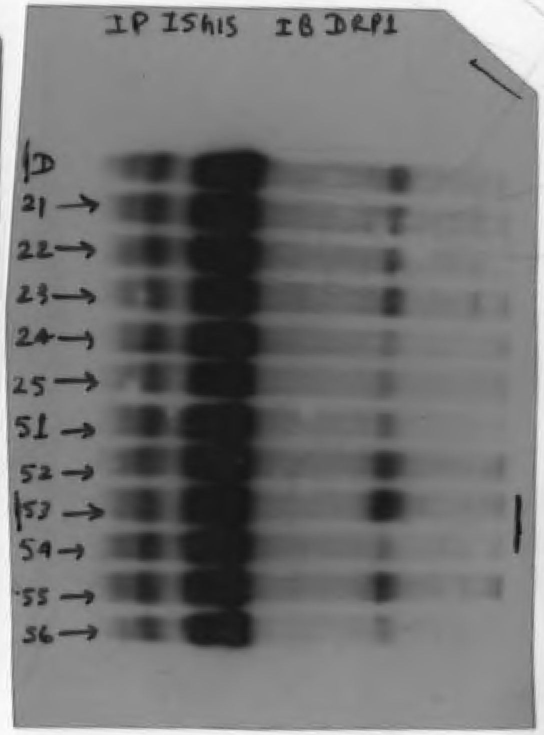
IP ISG15 IB DRP1
D
21
22
23
24
25
51
52
53
54
55
56

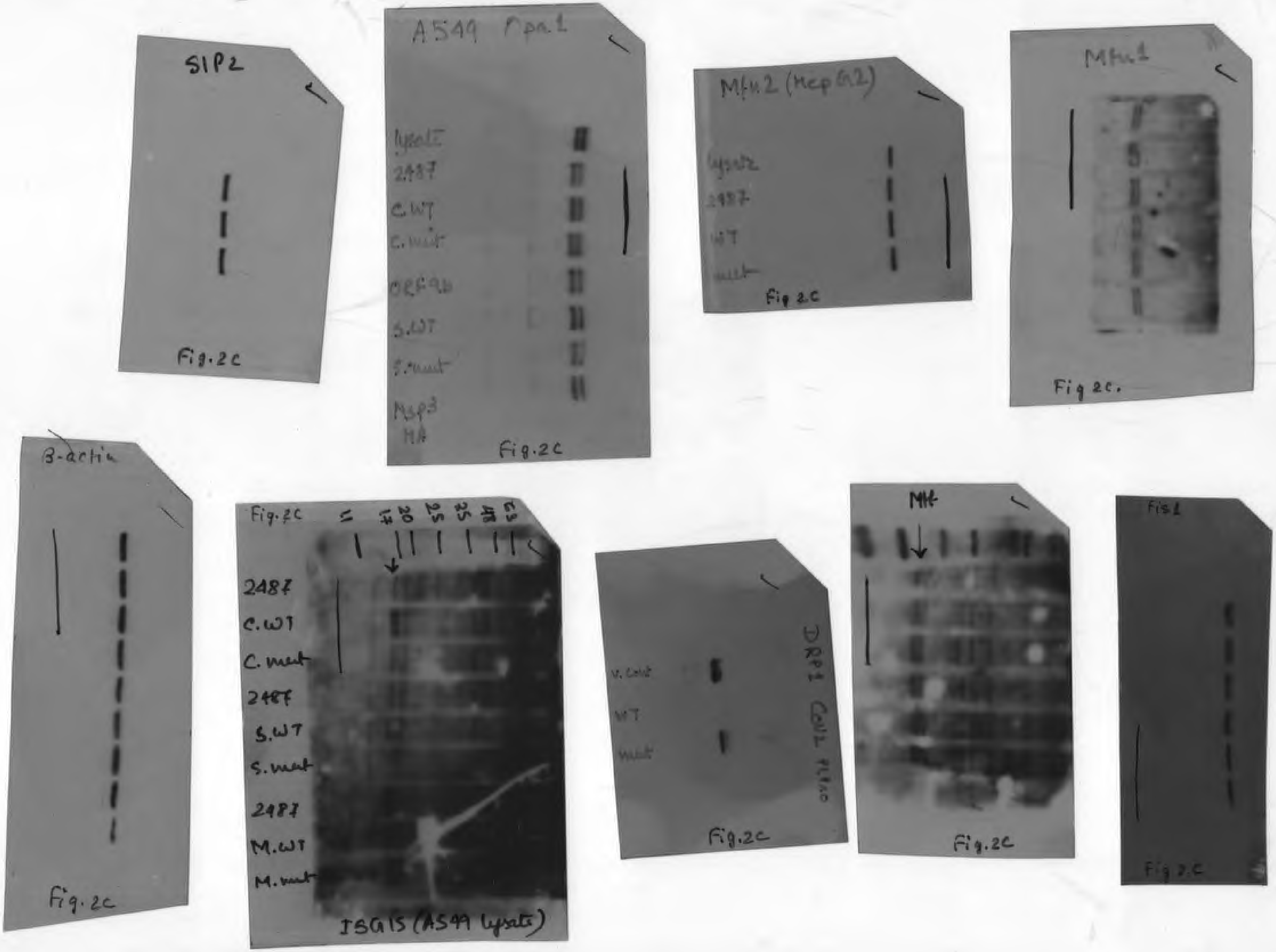
SIP2
Fig.2c
A549 Opa1
lysate
2487
C.WT
C.mut
ORF9b
S.WT
S.mut
Nsp3 HA
Fig.2c
Mfn2 (HepG2)
lysate
2487
WT
mut
Fig 2c
Mfn1
Fig 2c.
B-actin
Fig.2c
Fig.2c
11
17
20
25
35
48
63
2487
C.WT
C.mut
2487
S.WT
S.mut
2487
M.WT
M.mut
ISG15 (A549 lysate)
DRP1
v.cont
WT
mut
Fig.2c
Mff
Fig.2c
Fis1
Fig 2.c

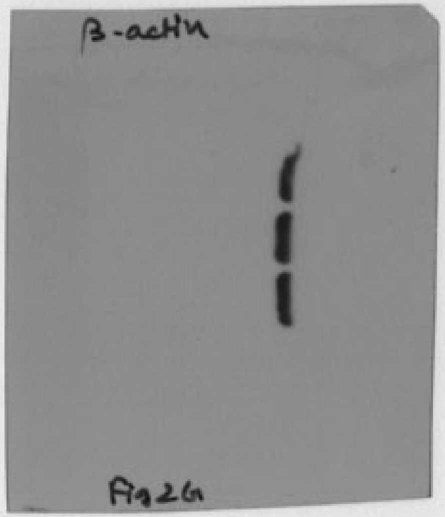
β-actin
Fig 2G

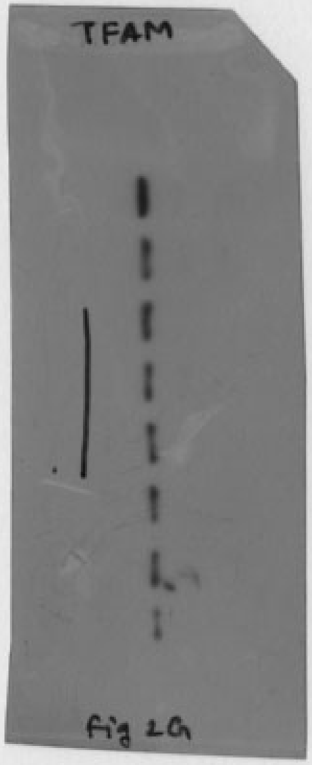
TFAM
Fig 2G

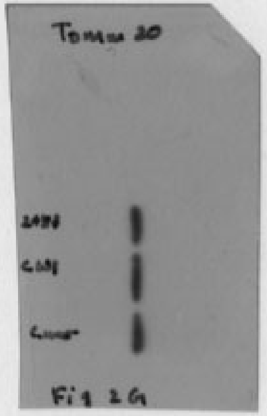
Tomm 20
Fig 2G

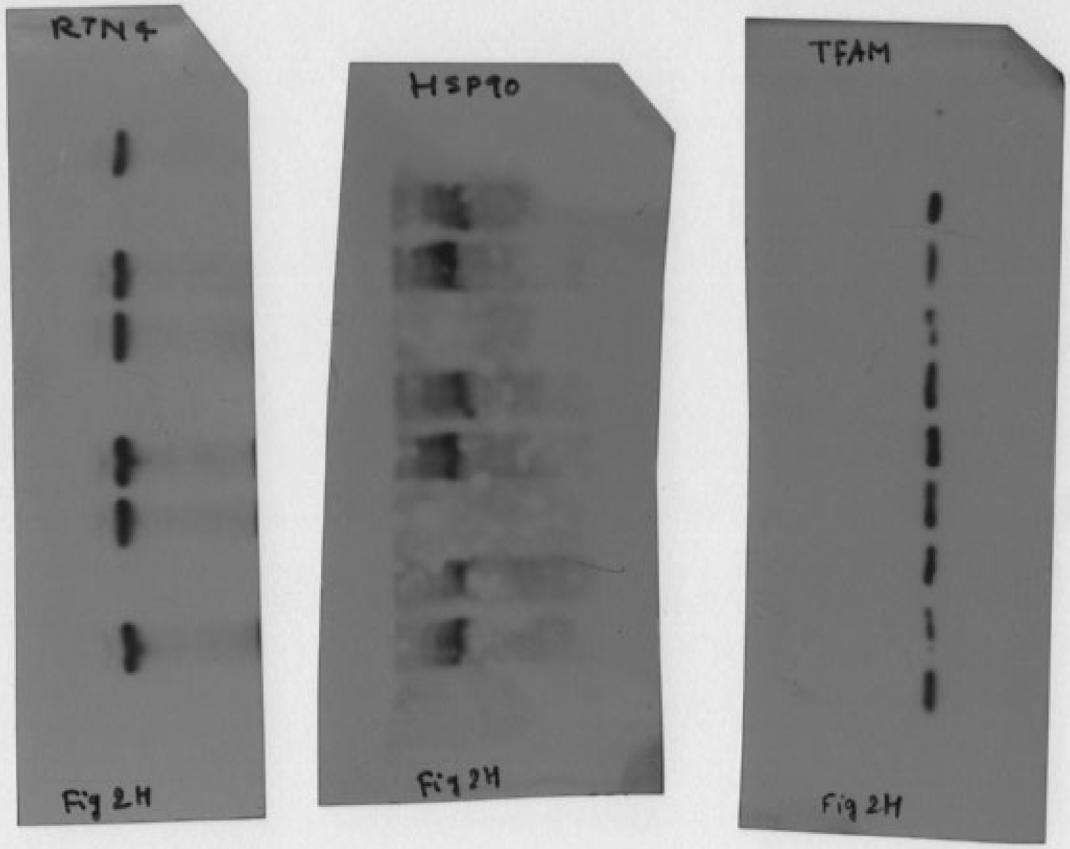
RTN4
Fig 2H
HSP90
Fig 2H
TFAM
Fig 2H

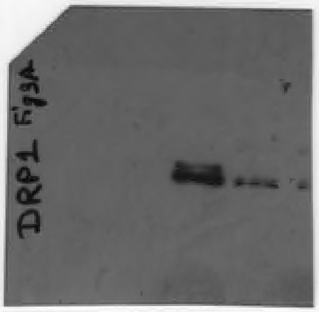
DRP1 Fig3A

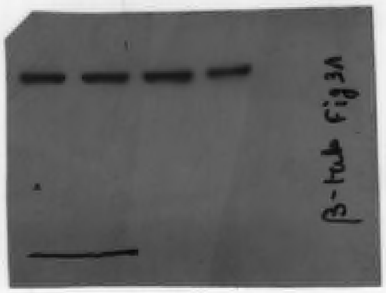
β-tub Fig3A

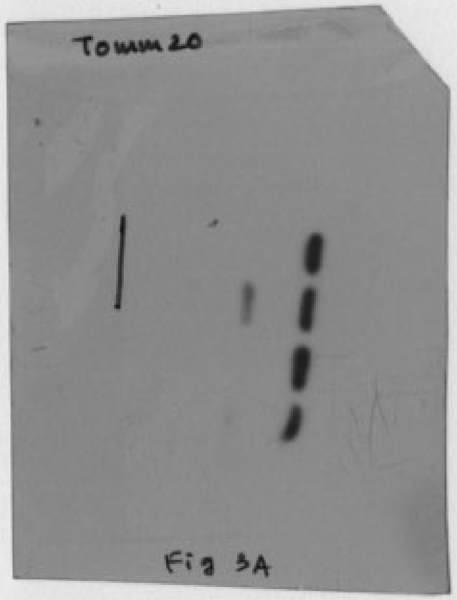
Tomm20
Fig 3A

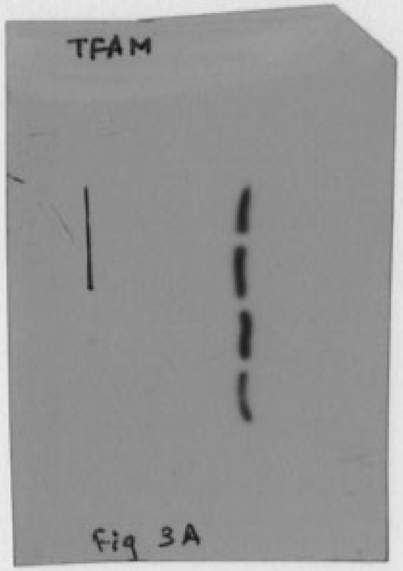
TFAM
Fig 3A

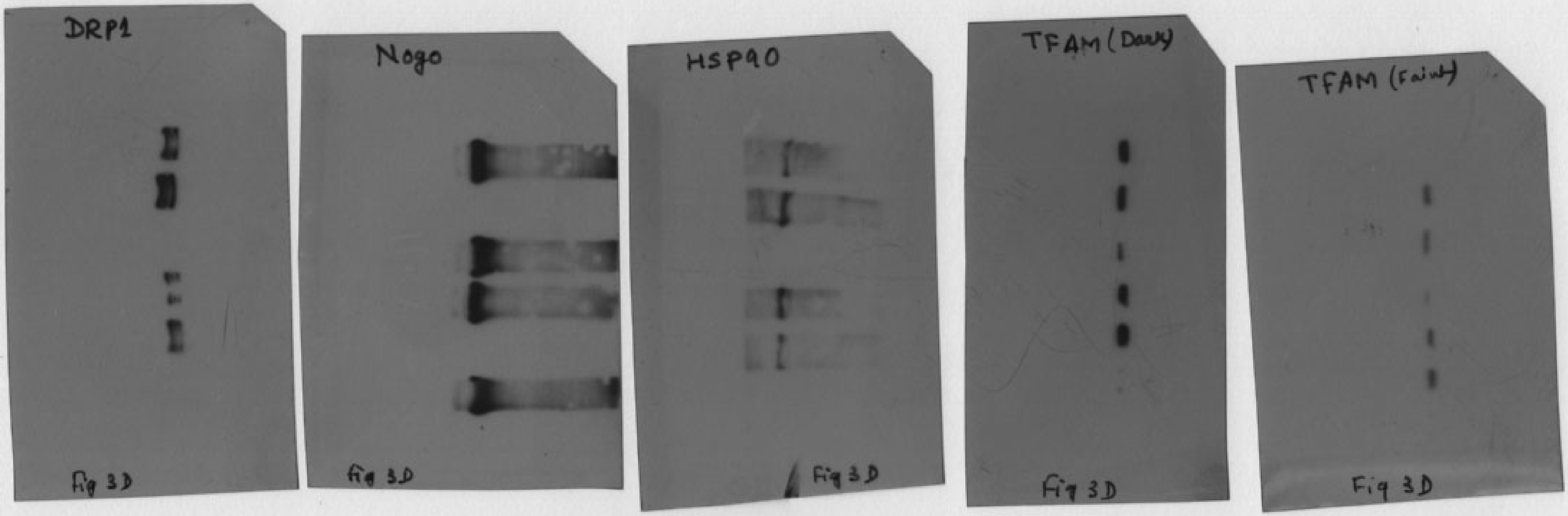
DRP1
Fig 3D
Nogo
Fig 3D
HSP90
Fig 3D
TFAM (Dark)
Fig 3D
TFAM (Faint)
Fig 3D

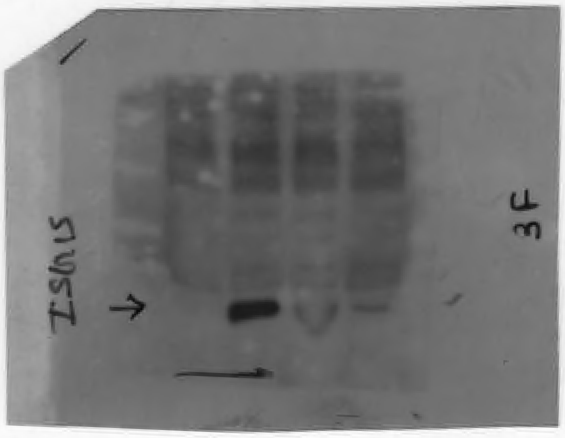
ISG15
3F

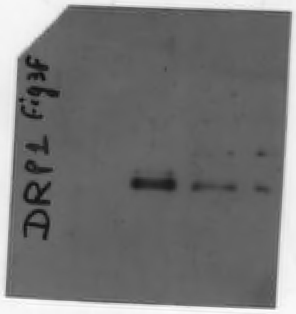
DRP1 Fig3F

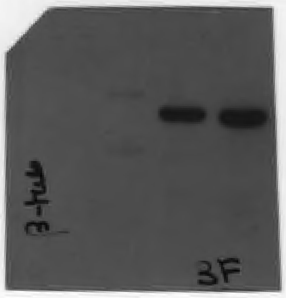
β-tub
3F

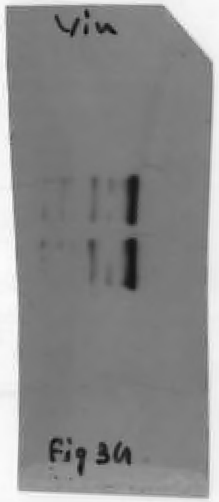
Vin
Fig 3A

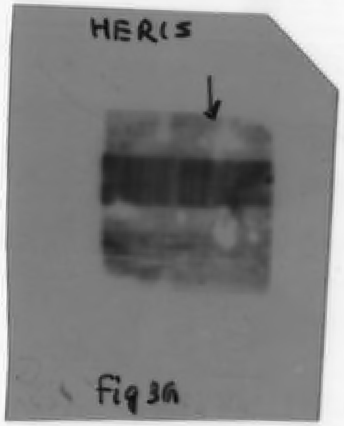
HER2S
Fig 3A

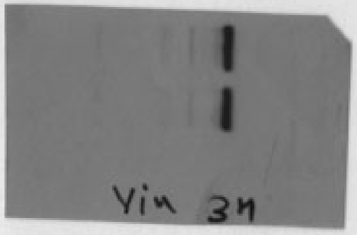
Yin
3H
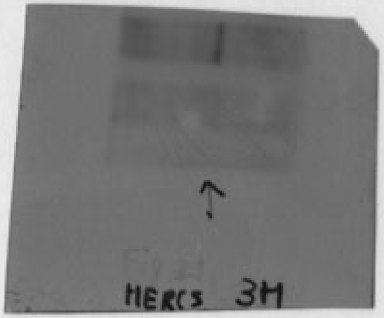
HERCS
3H
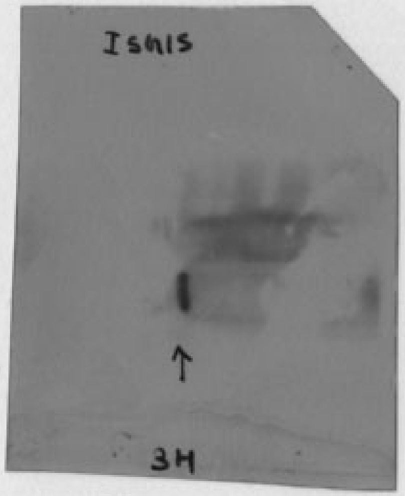
Ishis
3H
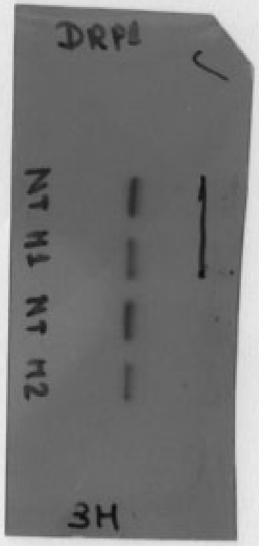
DRP1
NT M1 NT M2
3H

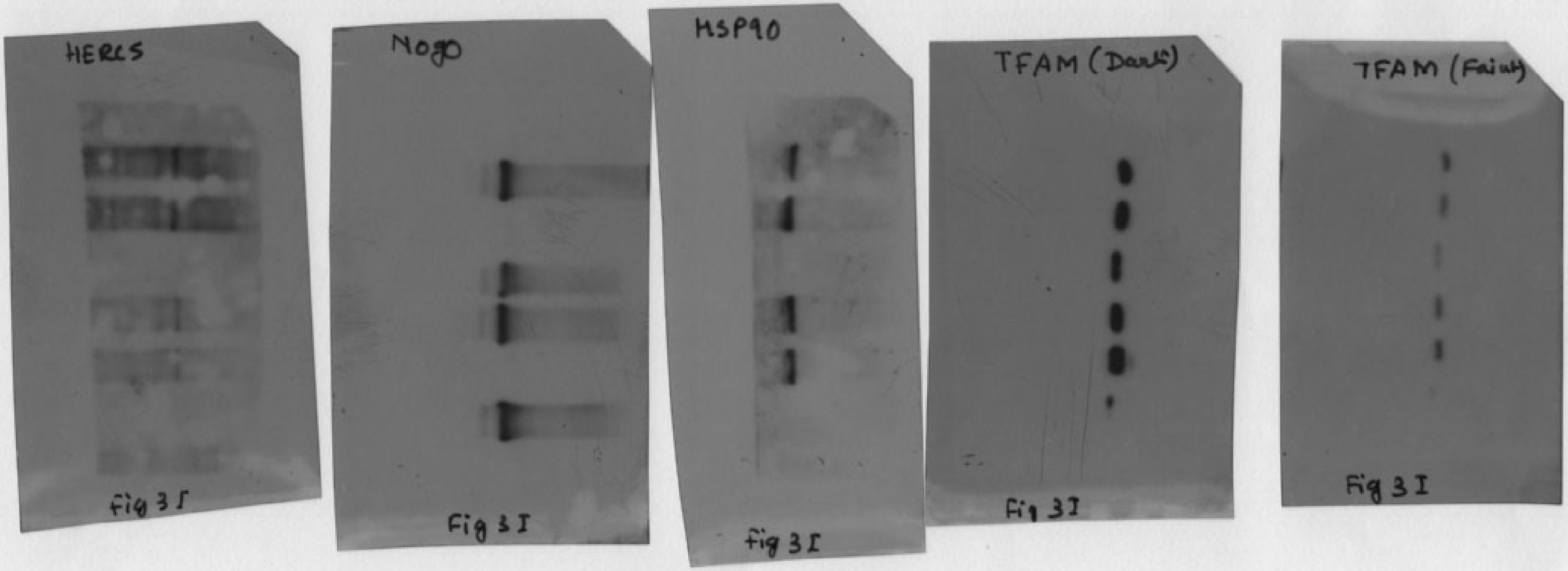
HERC5
Fig 3 I
Nogo
Fig 3 I
HSP90
Fig 3 I
TFAM (Dark)
Fig 3 I
TFAM (Faint
Fig 3 I

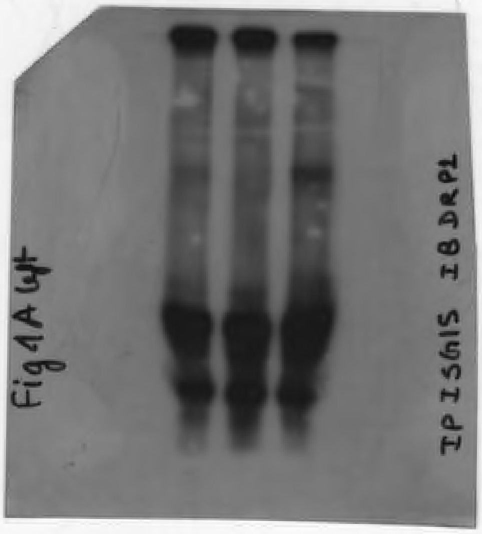
Fig4A left
IP ISG15 IB DRP1

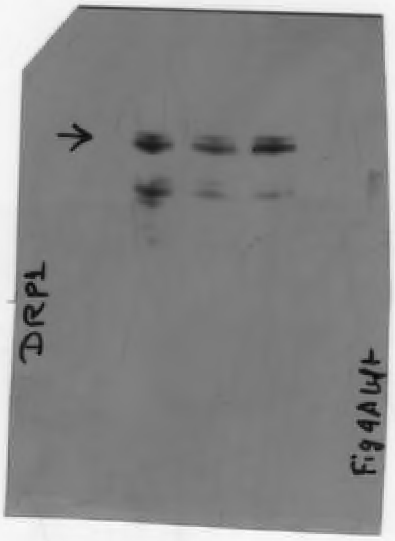
DRP1
Fig4A left

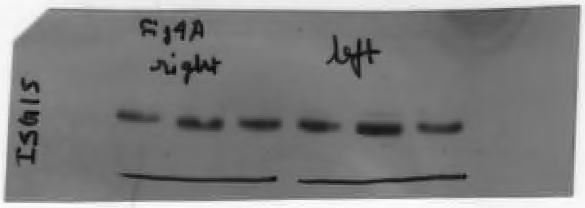
ISG15
Fig4A right
left

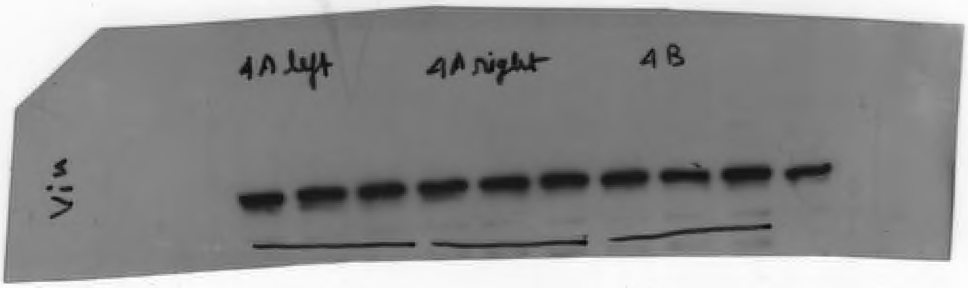
Vin
4A left
4A right
4B

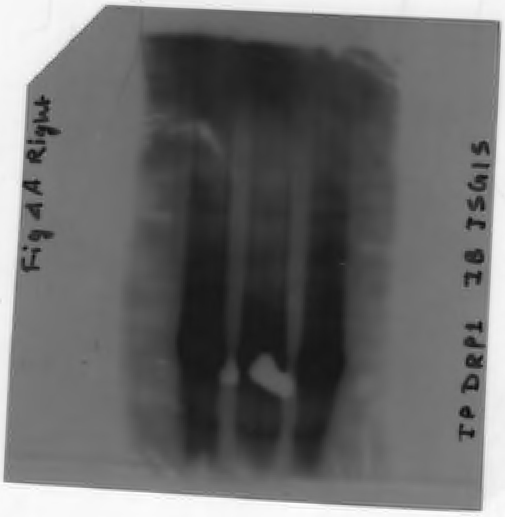
Fig 4A Right
IP DRP1 IB ISG15

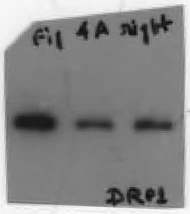
Fig 4A right
DRP1

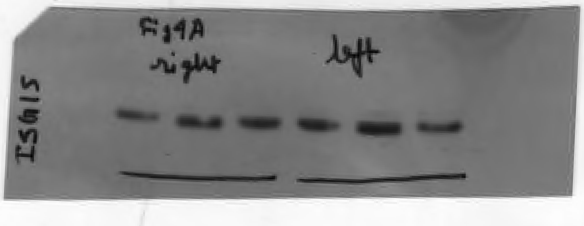
ISG15
Fig 4A right
left

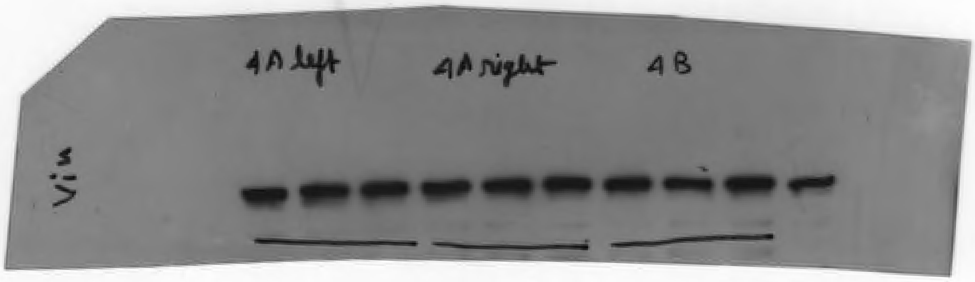
Vin
4A left
4A right
4B

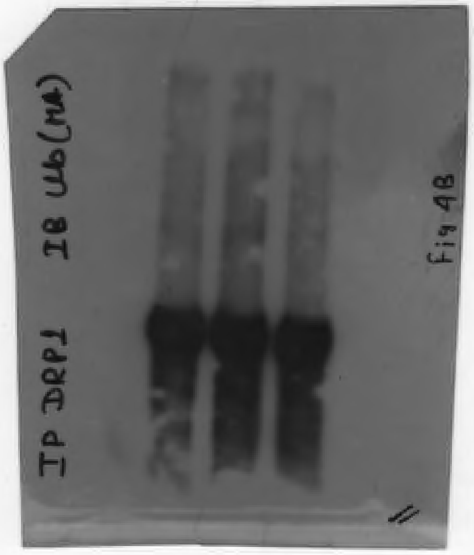
IP DRP1 IB Ub(HA)
Fig 4B

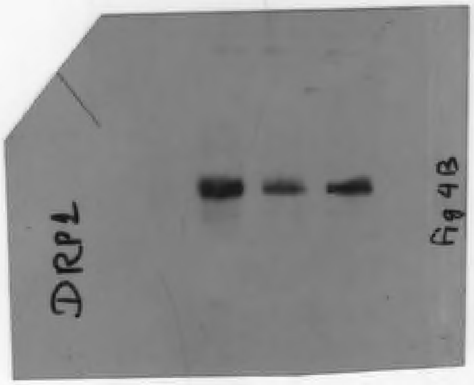
DRP1
Fig 4B

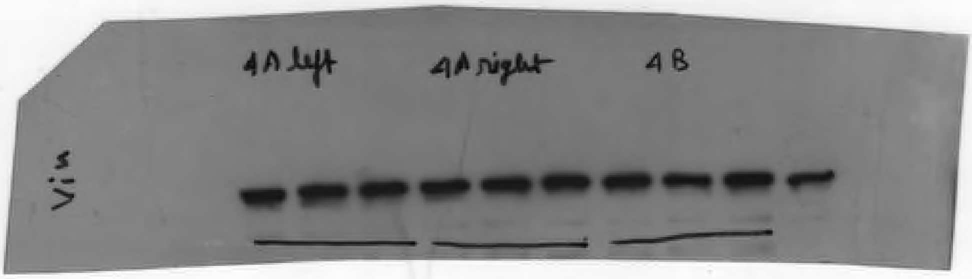
4A left
4A right
4B
Vin

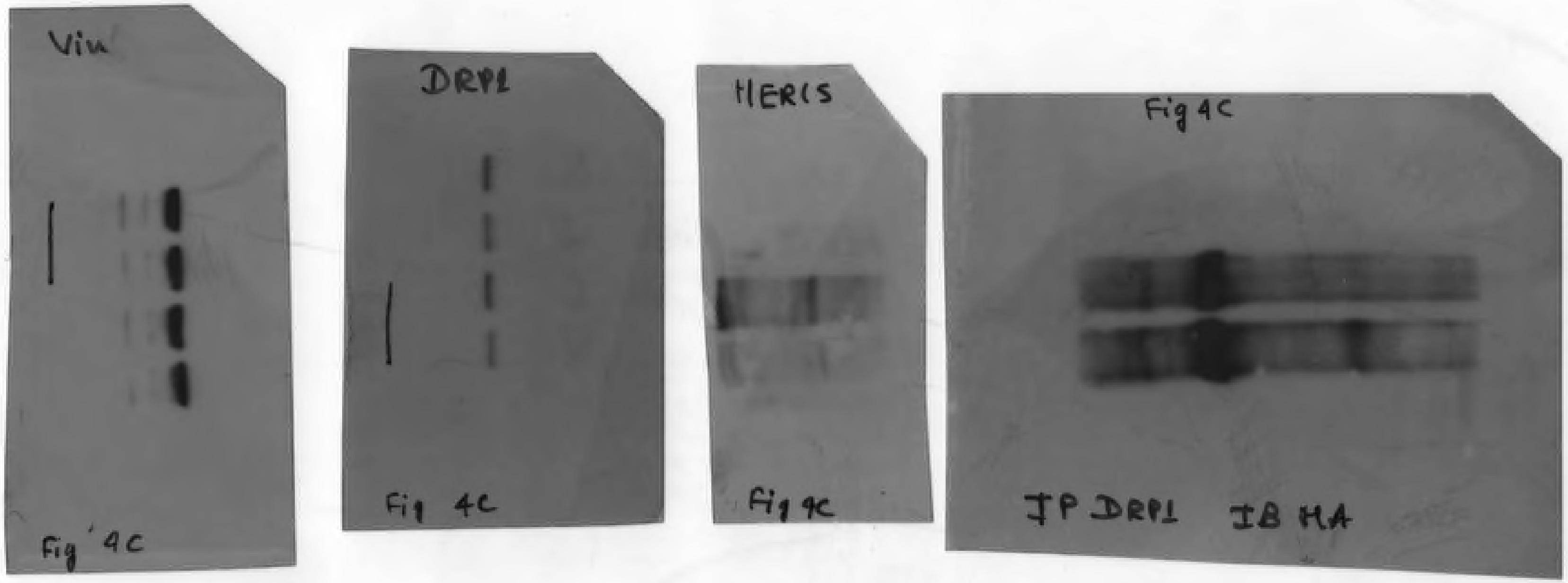
Vin
Fig 4C
DRP1
Fig 4C
HERC5
Fig 4C
Fig 4C
IP DRP1 IB HA

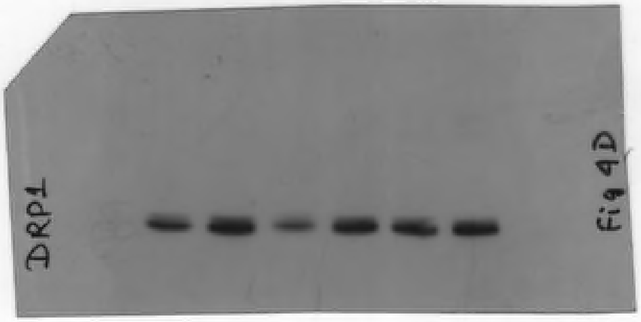
DRP1
fig 4D

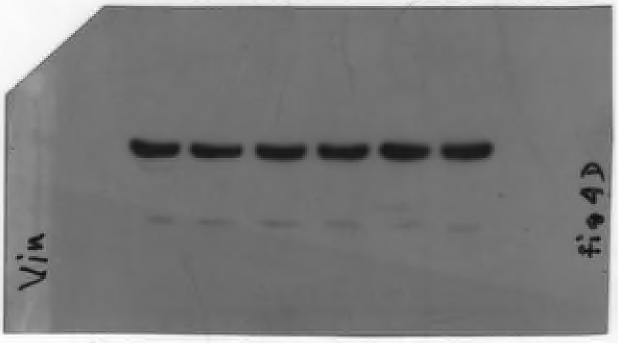
Vin
fig 4D

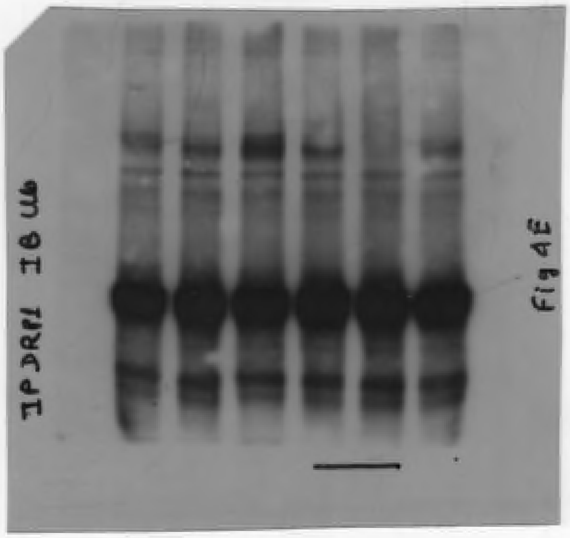
IP DRP1 IB Ub
Fig 4E

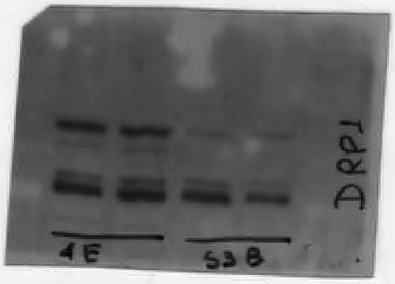
DRP1
4E
S3B

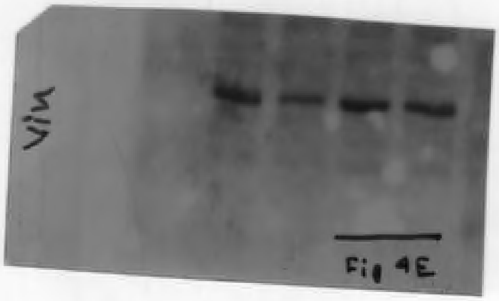
Vin
Fig 4E

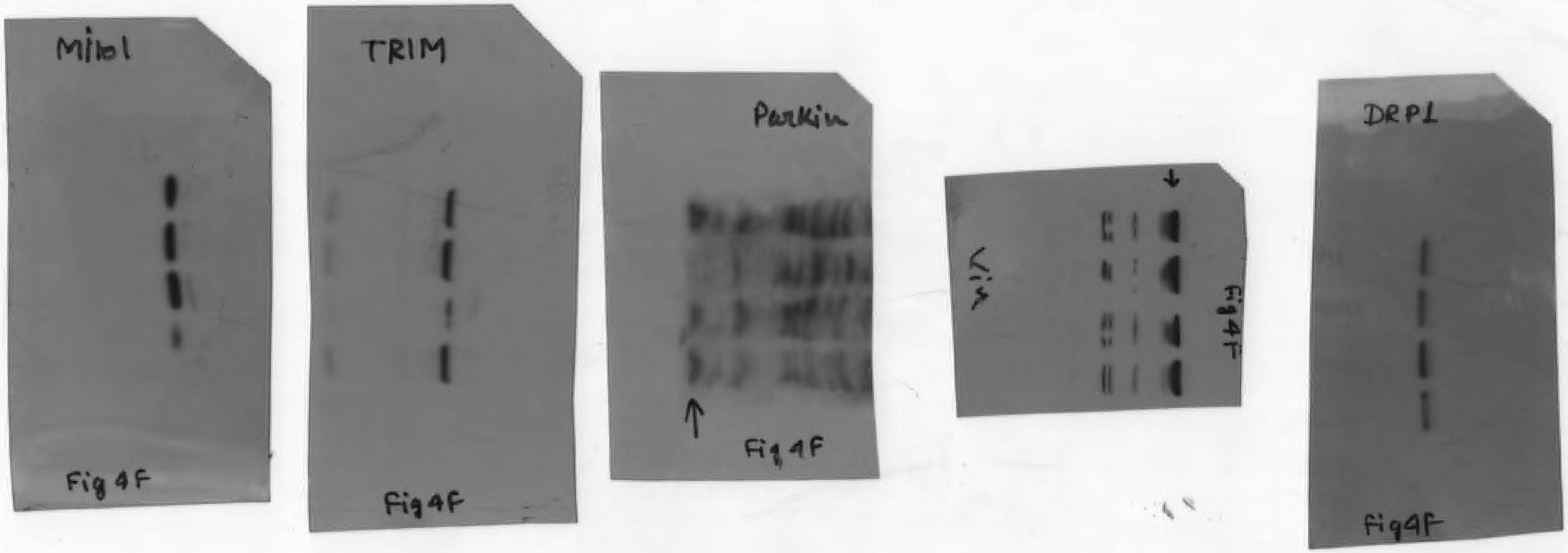
Mib1
Fig 4F
TRIM
Fig4F
Parkin
Fig 4F
Vin
Fig4F
DRP1
Fig4F

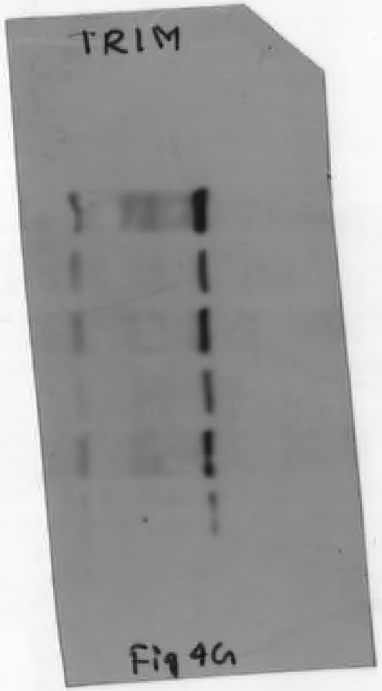
TRIM
Fig 4G
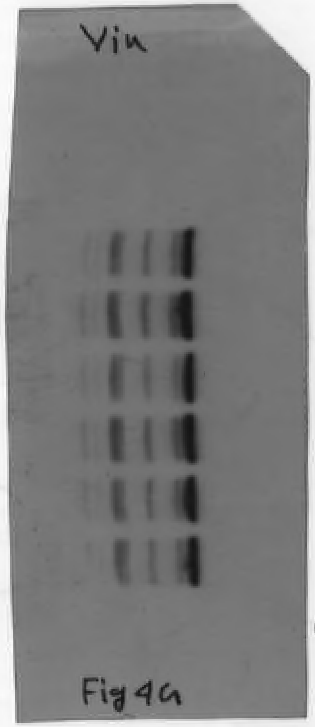
Vin
Fig 4G
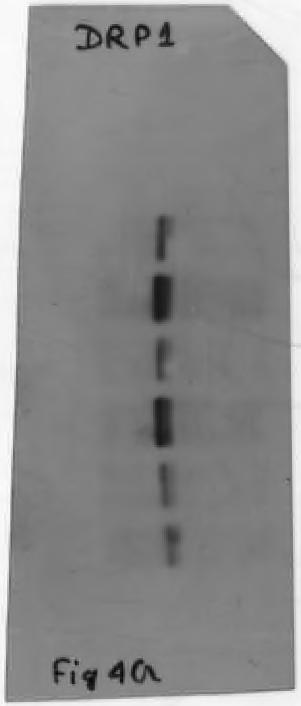
DRP1
Fig 4G

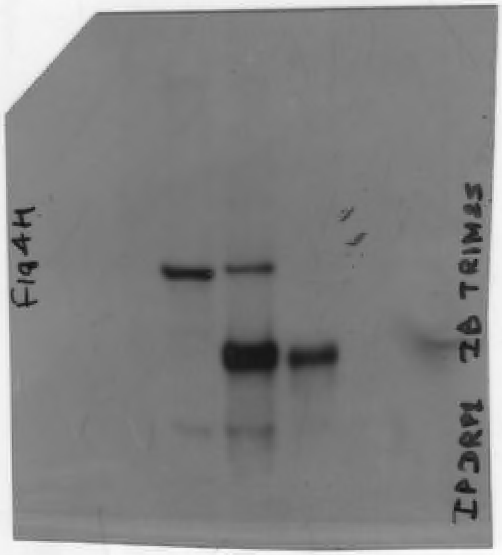
Fig4H
IB TRIM25

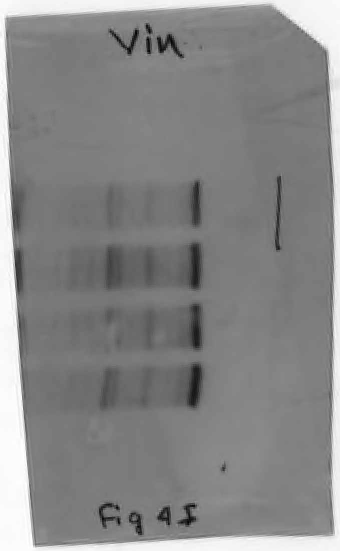
Vin
Fig 4I

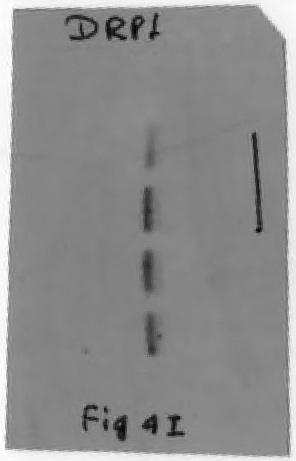
DRP1
Fig 4I

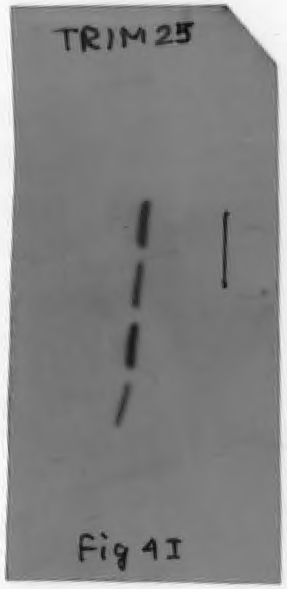
TRIM25
Fig 4I

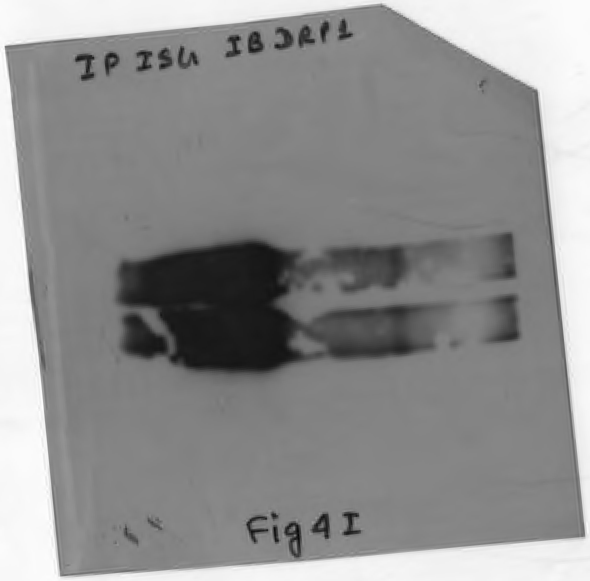
IP ISG IB DRP1
Fig 4I

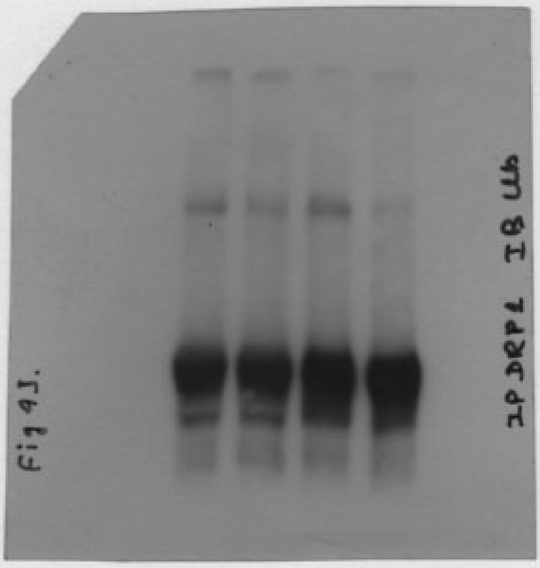
Fig 4J.
IP DRP1 IB Ub

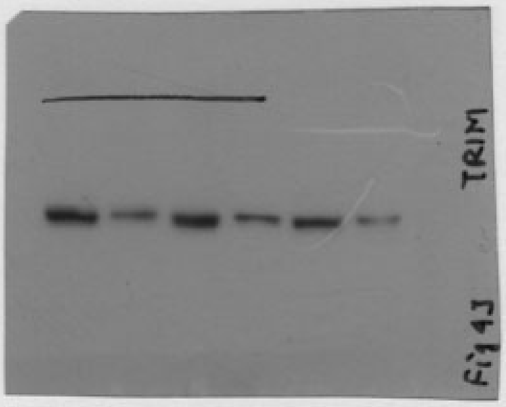
TRIM
Fig 4J

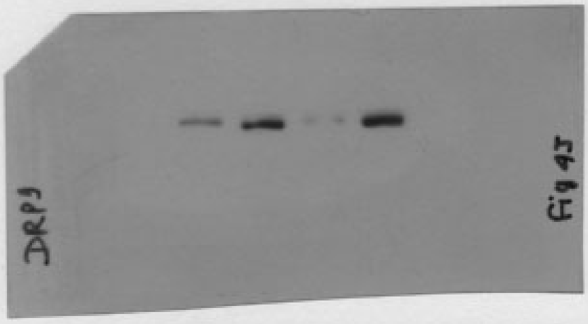
DRP1
Fig 4J

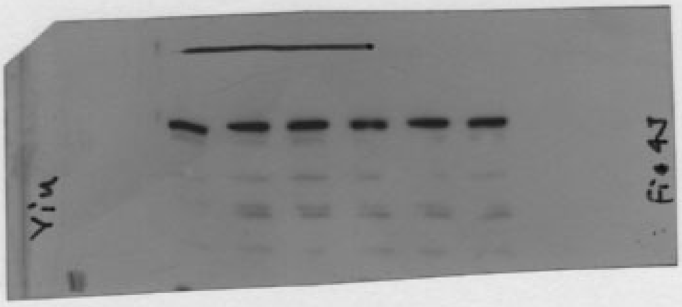
Vin
Fig 4J

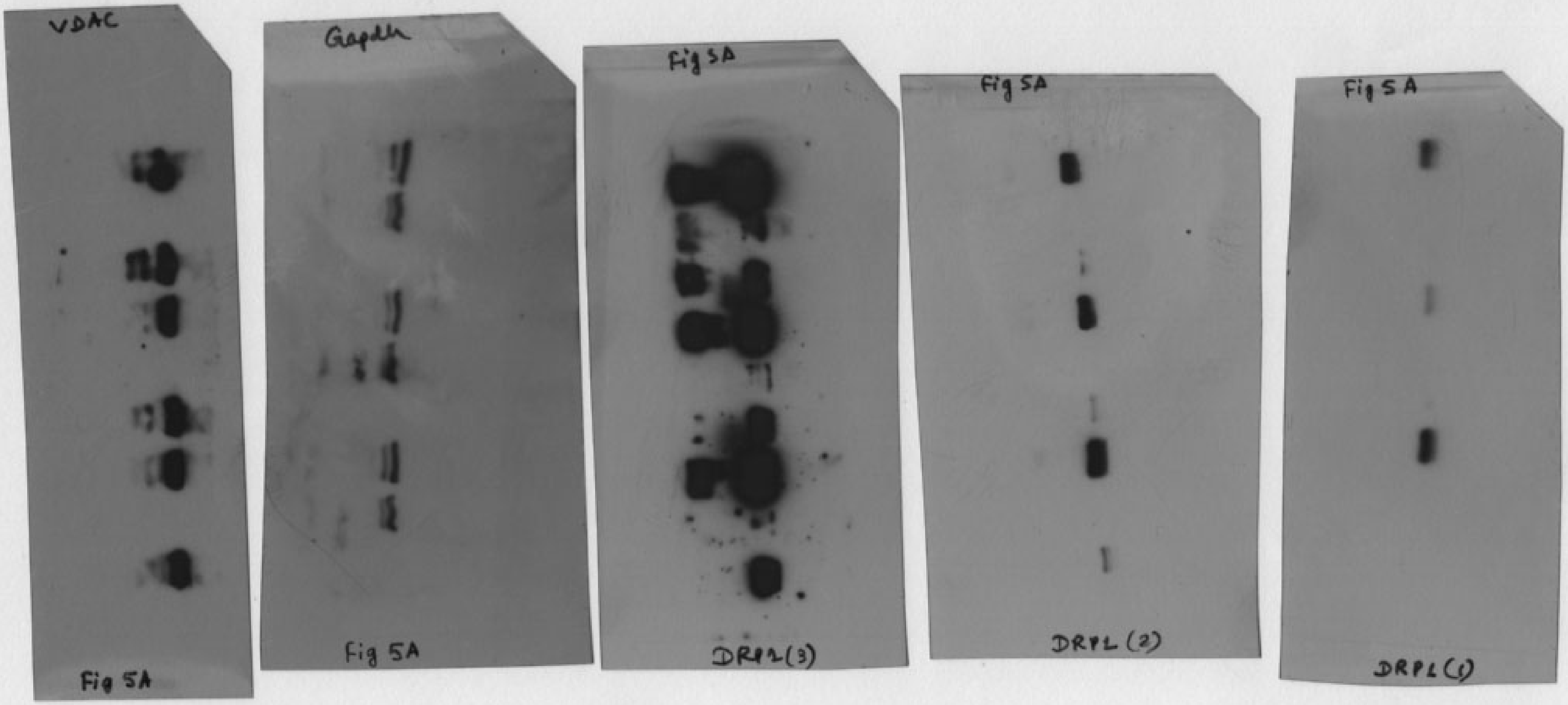
VDAC
Fig 5A
Gapdh
Fig 5A
Fig 5A
DRP1(3)
Fig 5A
DRP1(2)
Fig 5A
DRP1(1)

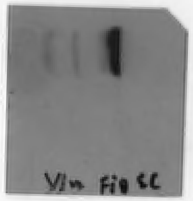

Vin Fig 5C

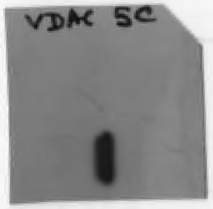

VDAC 5C

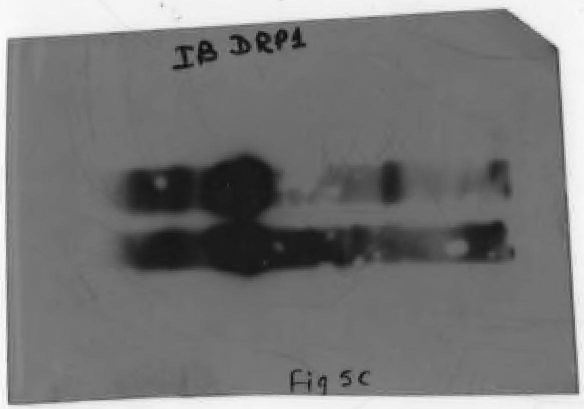

IB DRP1
Fig 5C

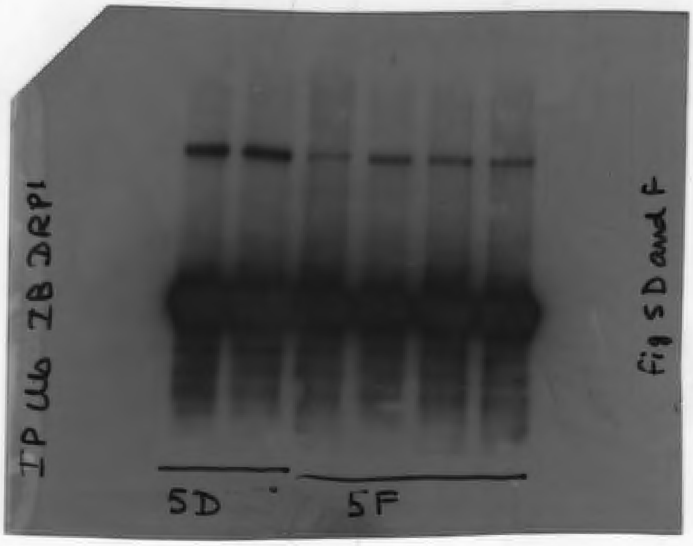
IP Ub IB DRP1
5D
5F
fig 5D and F

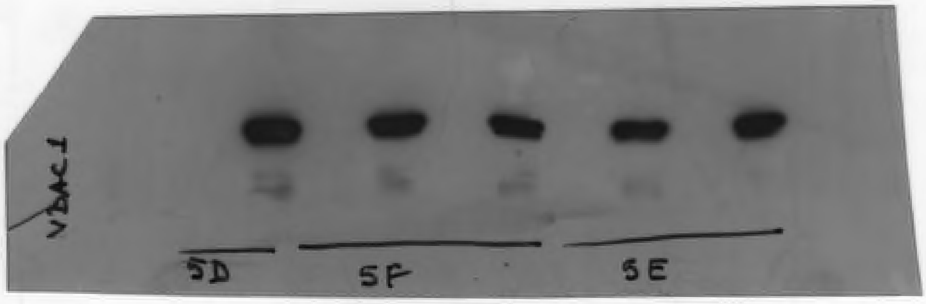
VDAC1
5D
5F
5E

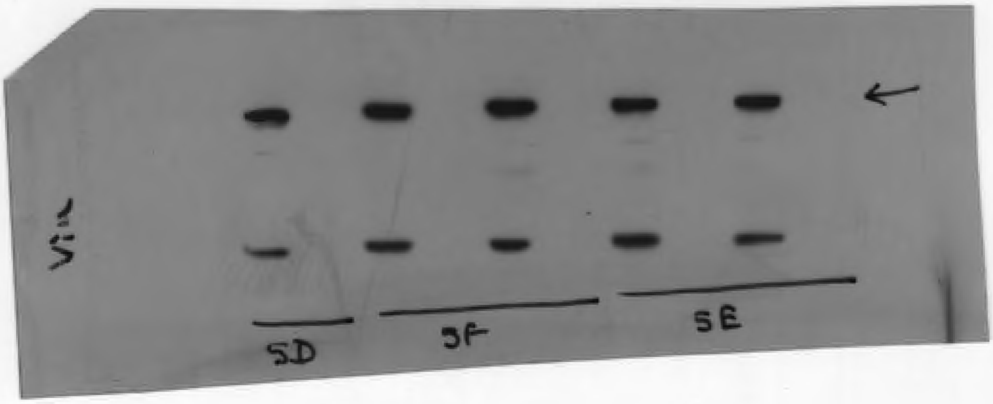
Vin
5D
5F
5E

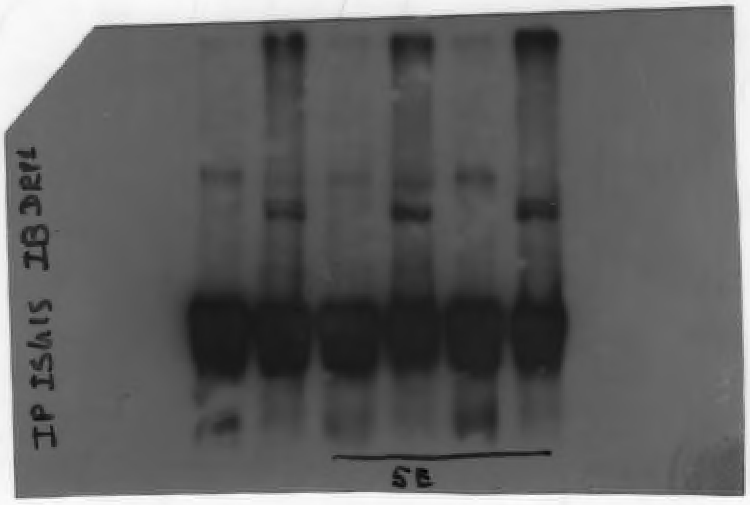
IP ISG15 IB DRP1
5E

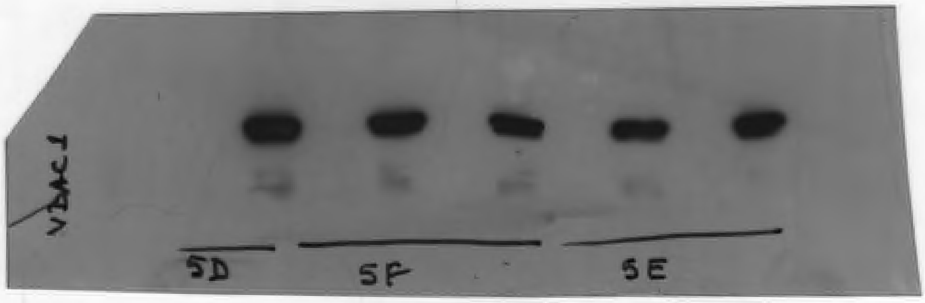
VDAC1
5D
5F
5E

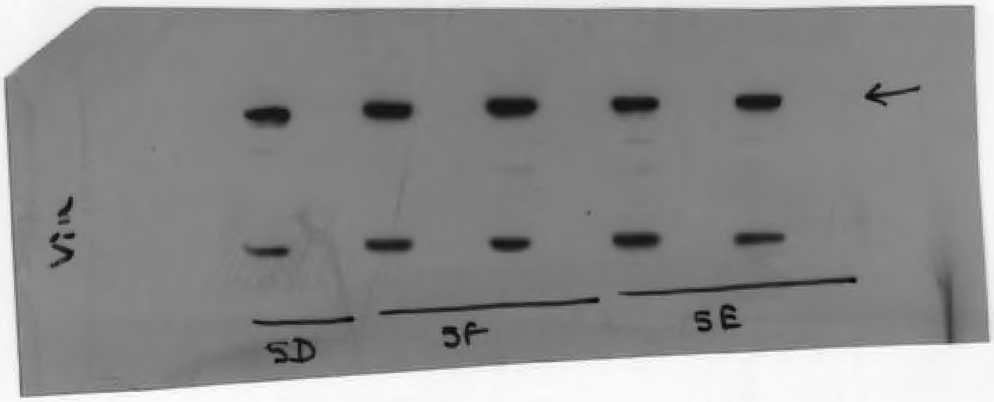
Vin
5D
5F
5E

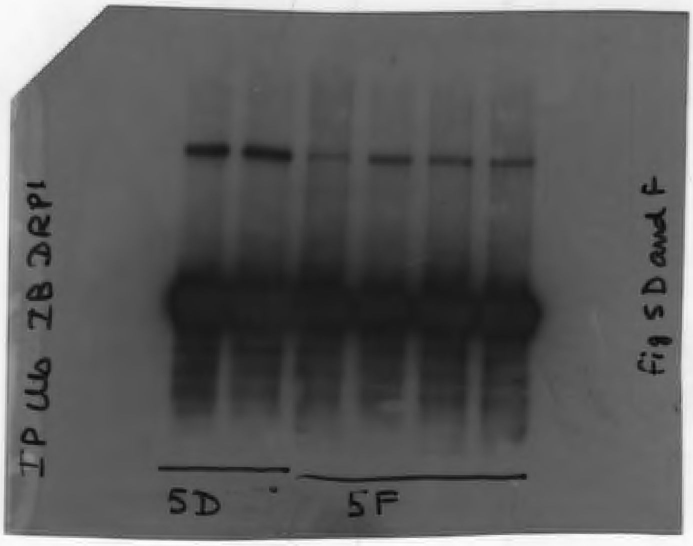
IP Ub IB DRP1
fig 5D and F
5D
5F

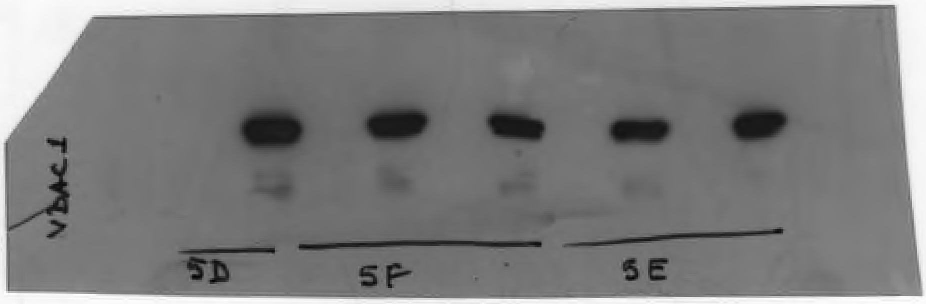
VDAC1
5D
5F
5E

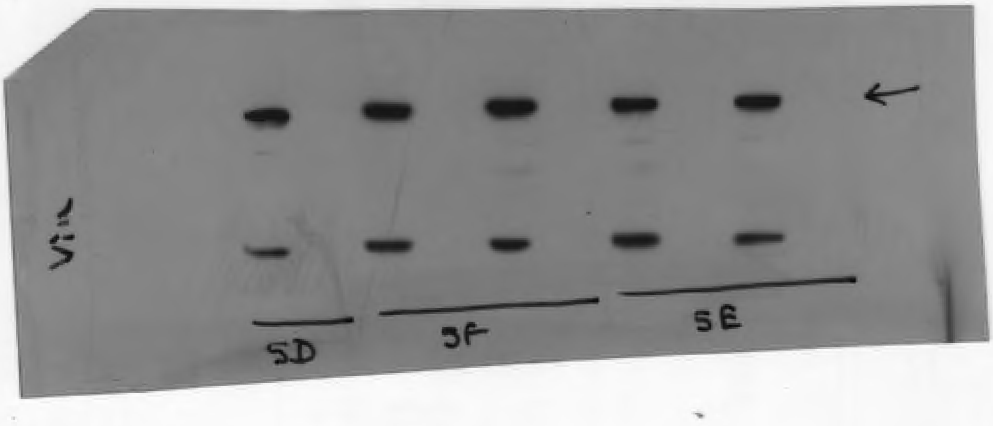
Vin
5D
5F
5E

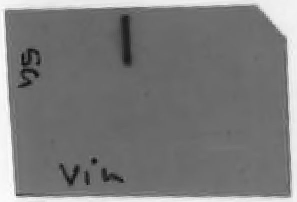
5G
Vin

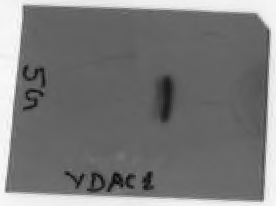
5G
VDAC1

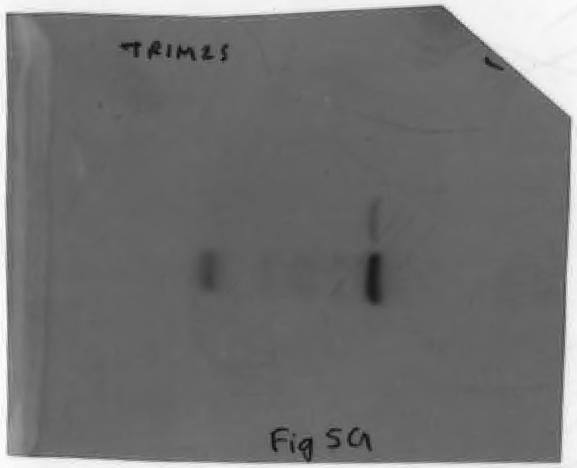
TRIM25
Fig 5G

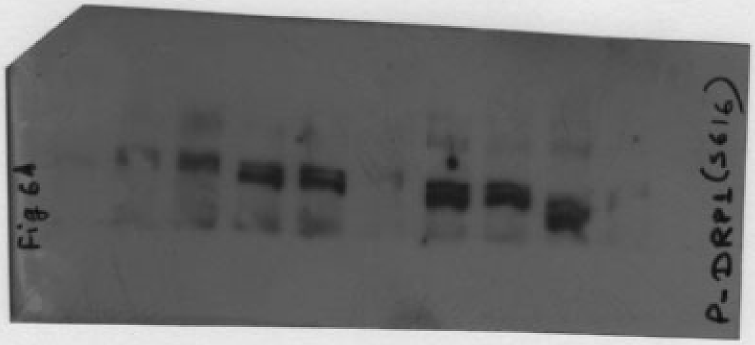
Fig 6A
P-DRP1(S616)

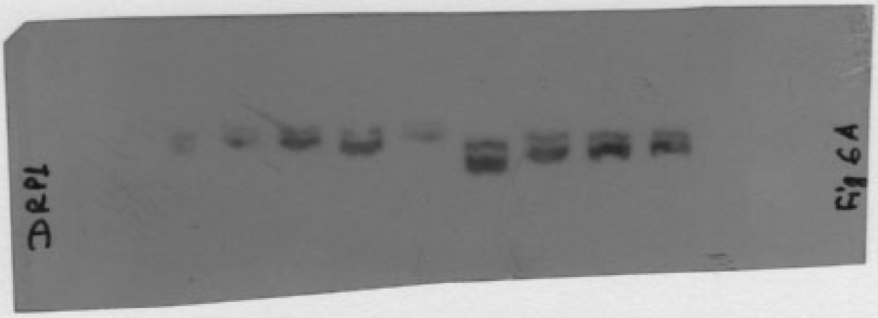
DRP1
Fig 6A

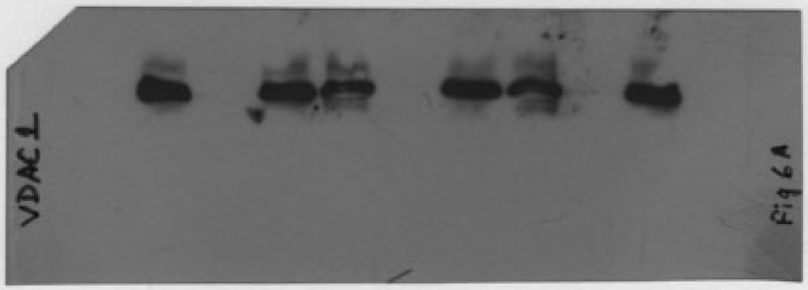
VDAC1
Fig 6A

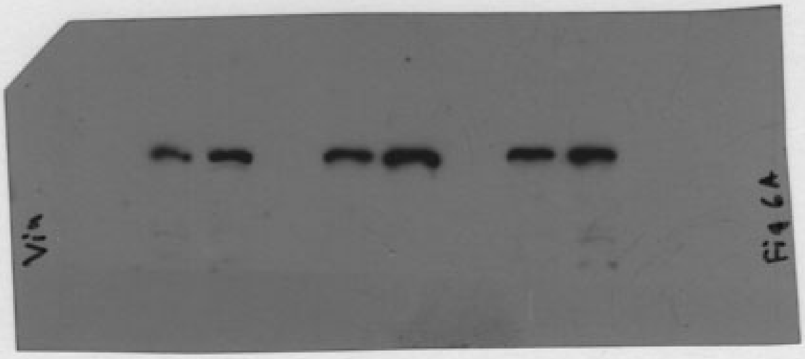
Vin
Fig 6A

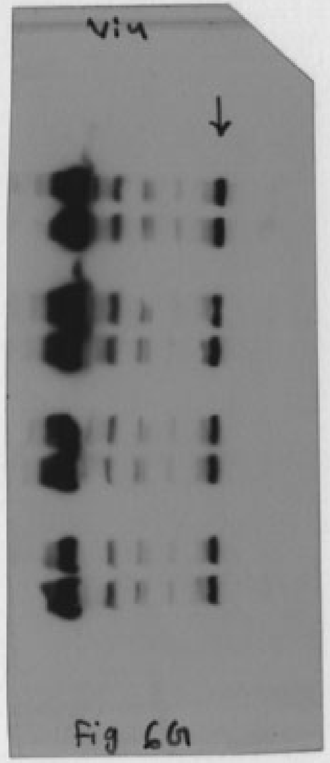
Vin
Fig 6G

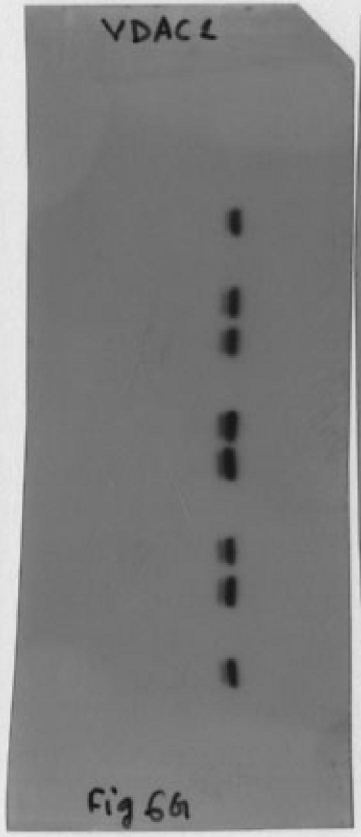
VDAC1
Fig 6G

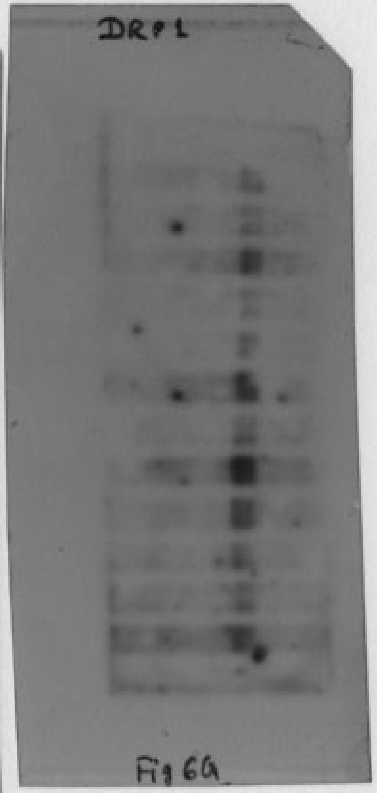
DRP1
Fig 6G

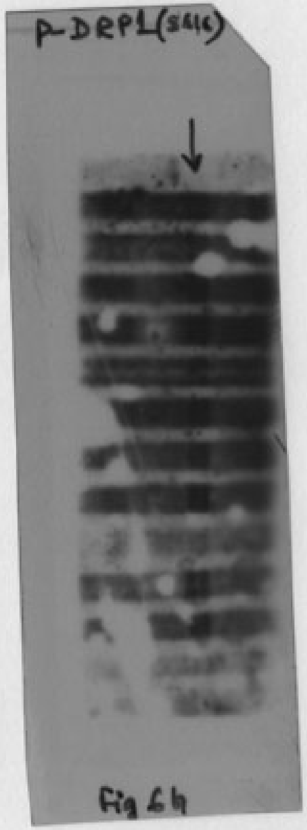
p-DRP1(S616)
Fig 6h

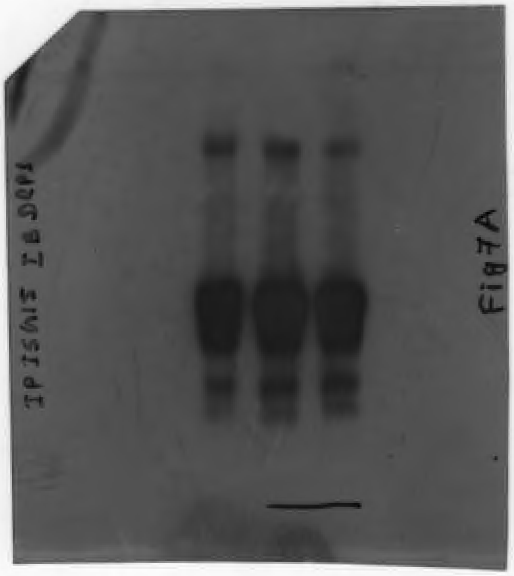
IP ISG15 IB DRP1
Fig7A

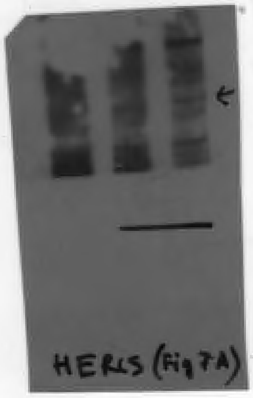
HERC5 (Fig7A)

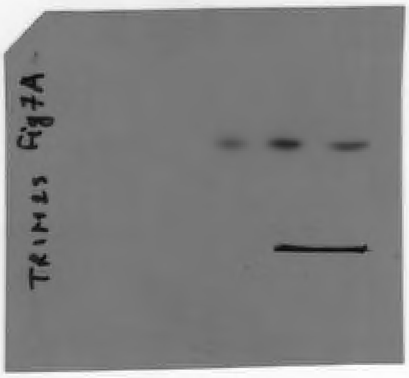
TRIM25 Fig7A

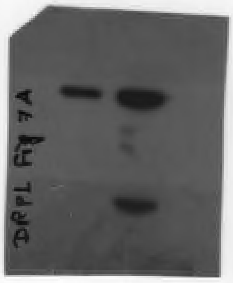
DRP1 Fig 7A

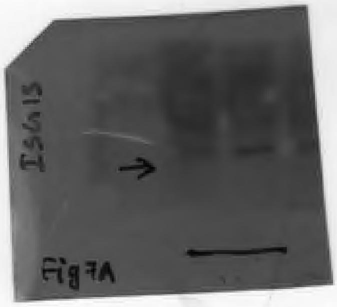
ISG15
Fig7A

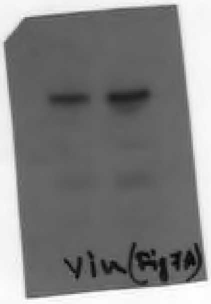
Vin (Fig7A)

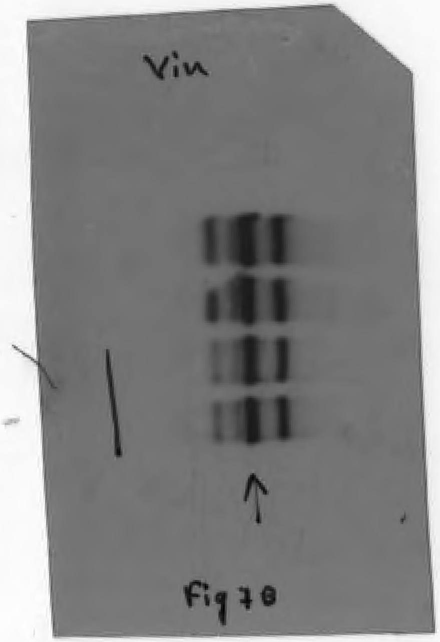
Vin
Fig 7B

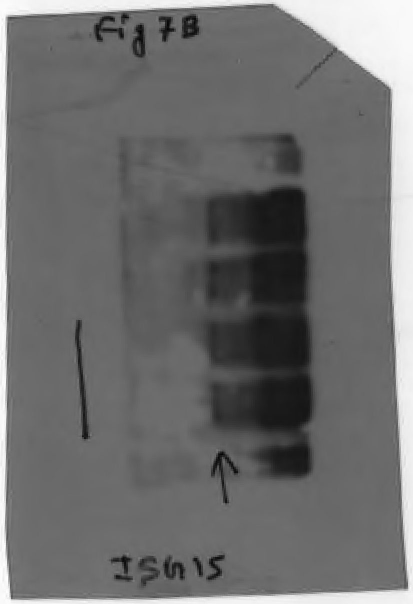
Fig 7B
ISG15

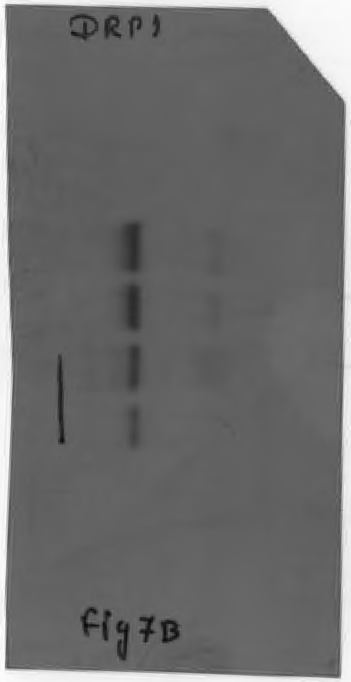
DRP1
Fig 7B

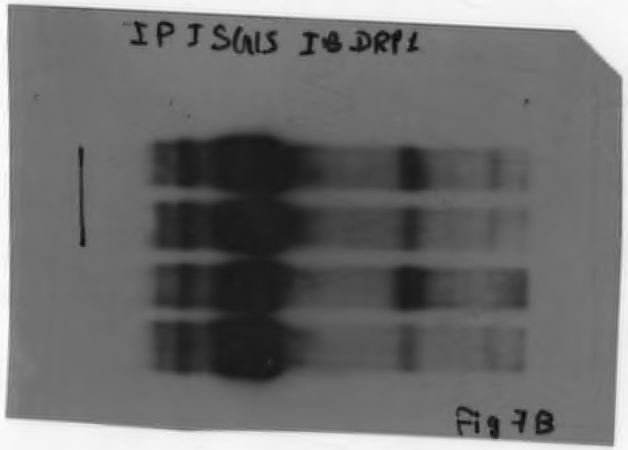
IP ISG15 IB DRP1
Fig 7B

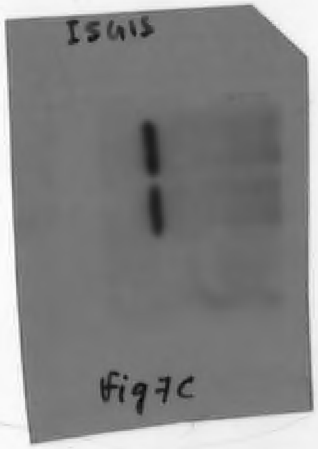
ISG15
Fig7C

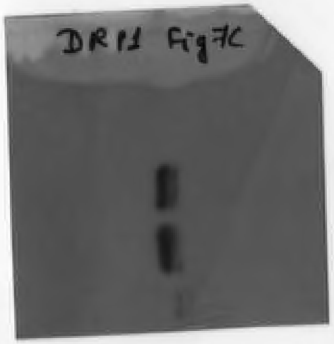
DRP1 Fig7C

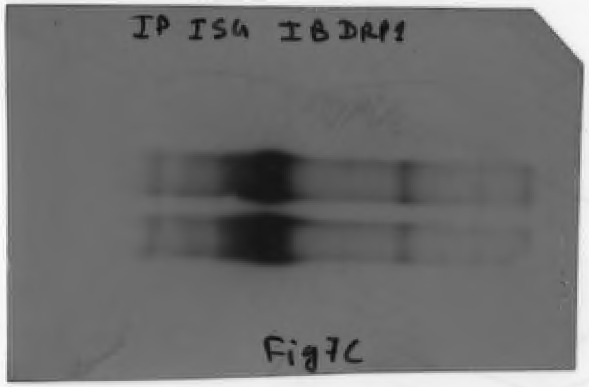
IP ISG IB DRP1
Fig7C

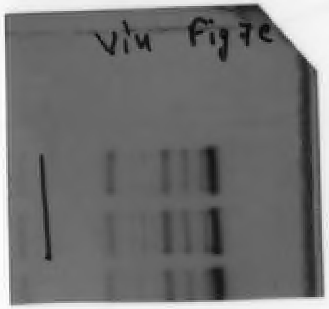
Vin Fig7C

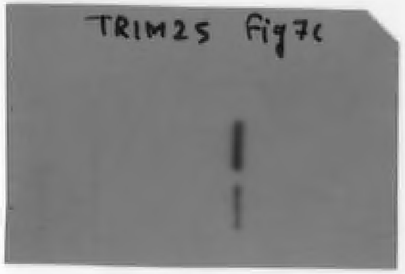
TRIM25 Fig7C

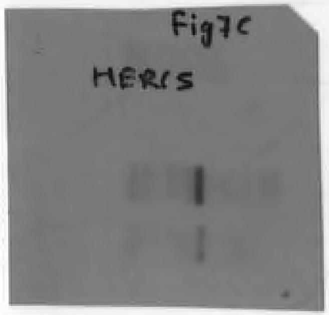
Fig7C
HERC5

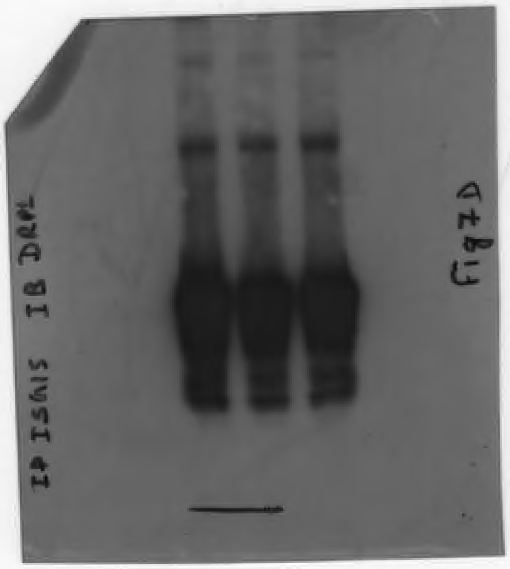
IP ISG15 IB Drp1
Fig7D

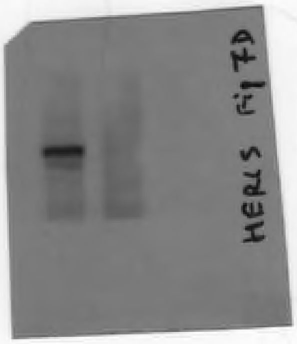
HERC5 Fig 7D

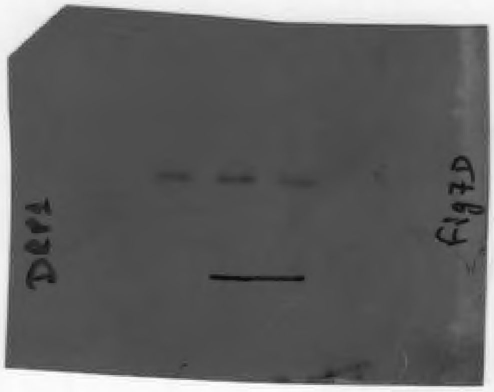
Drp1
Fig7D

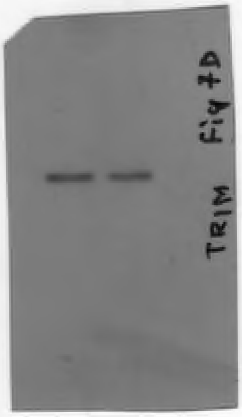
TRIM Fig 7D

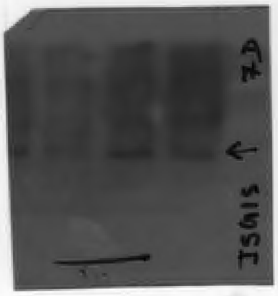
ISG15
7D

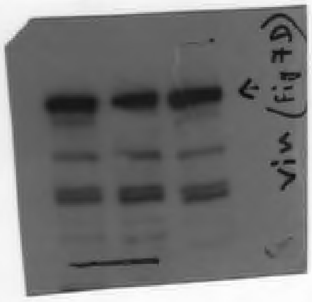
Vin (Fig 7D)

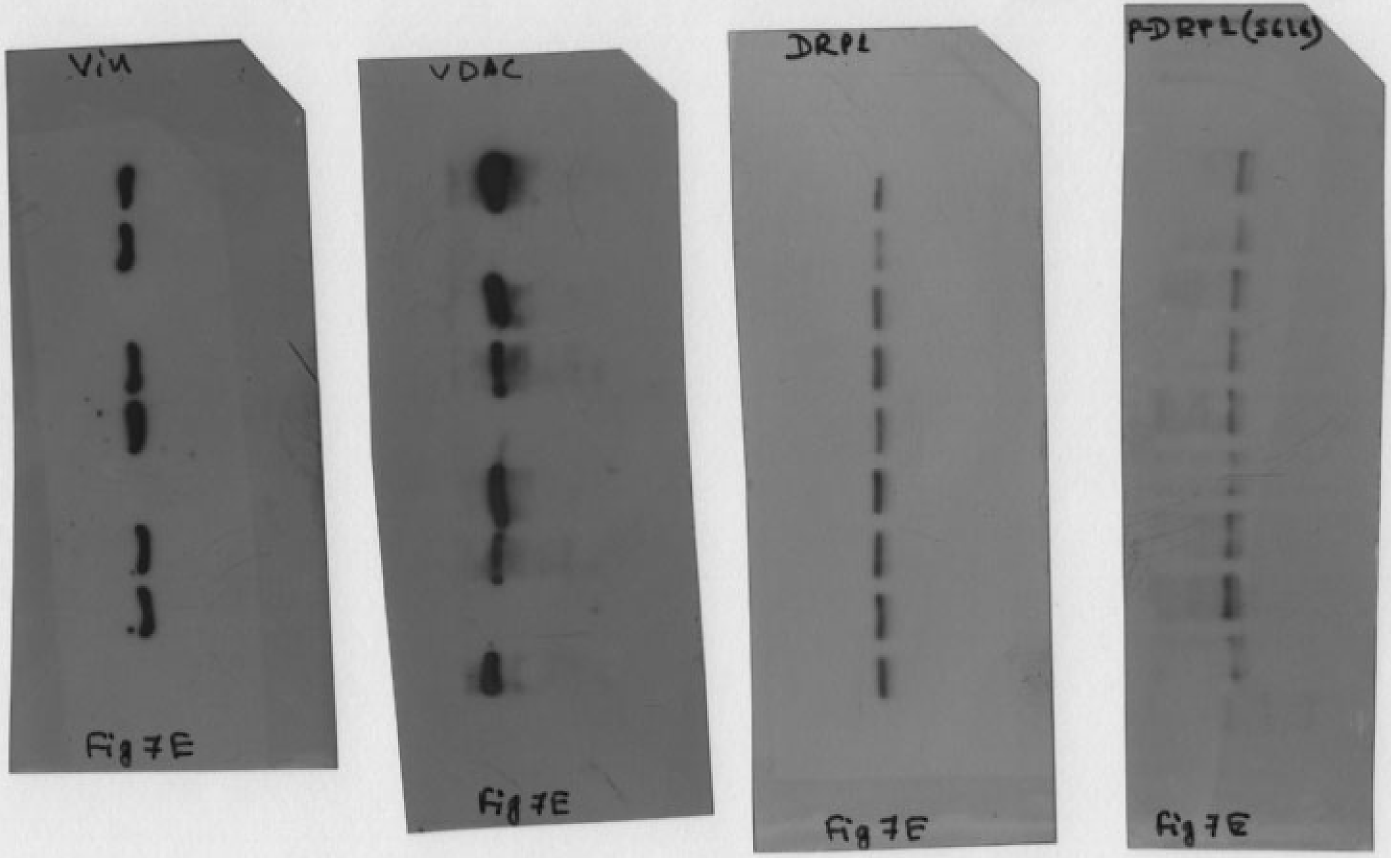
Vin
Fig 7E
VDAC
Fig 7E
DRP1
Fig 7E
P-DRP1(S616)
Fig 7E

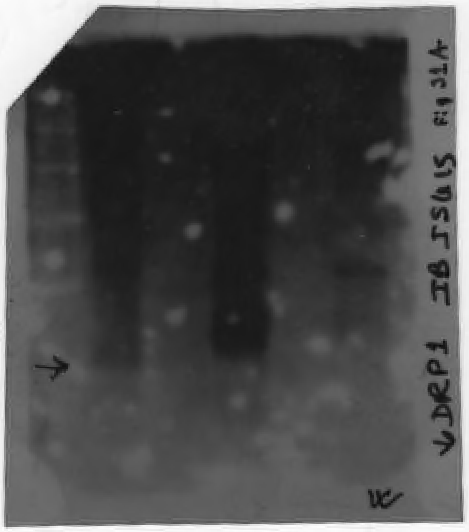
VDRP1 IB ISG15 Fig 31A

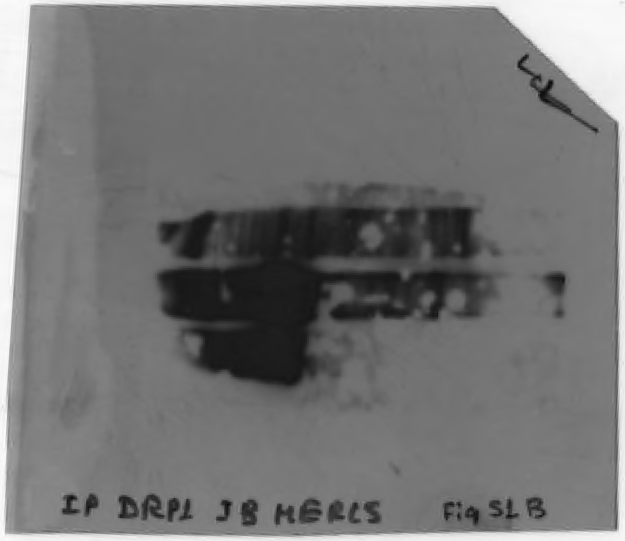
IP DRP1 IB HERLS Fig S1B

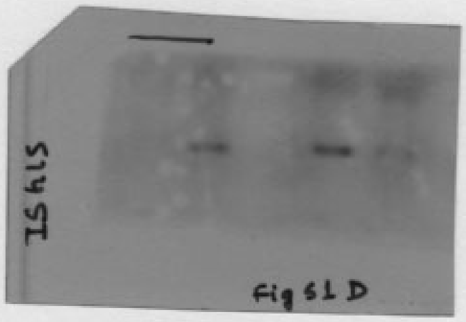
ISG15
Fig S1 D

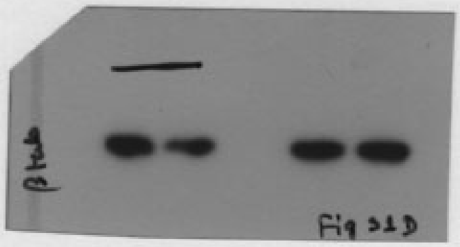
β tub
Fig S1 D

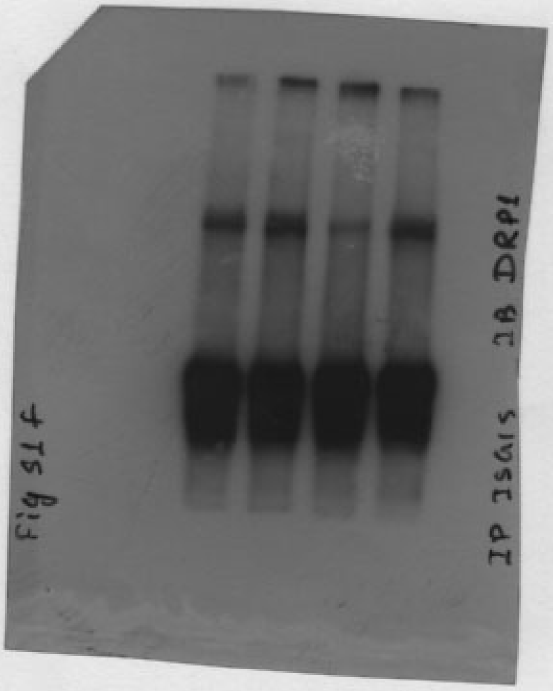
Fig S1 F
IP ISG15 IB DRP1

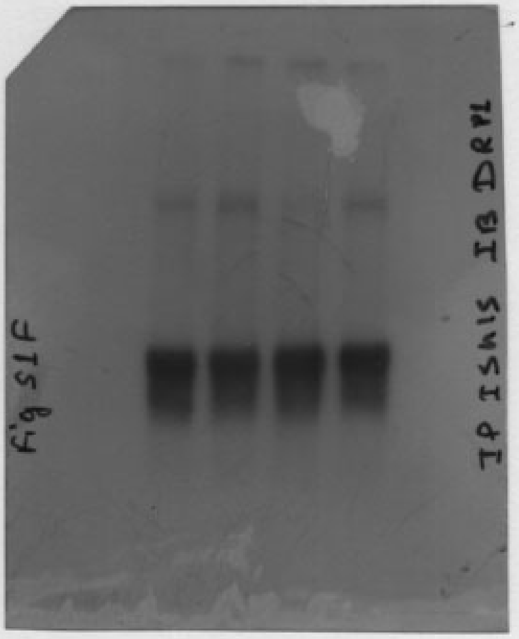
Fig S1F
IP ISG15 IB DRP1

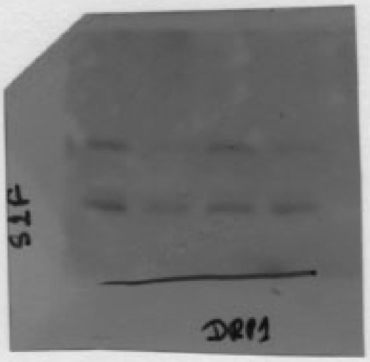
S1F
DRP1

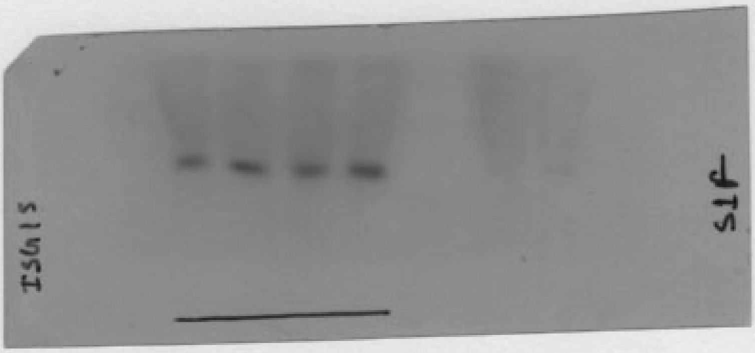
ISG15
S1F

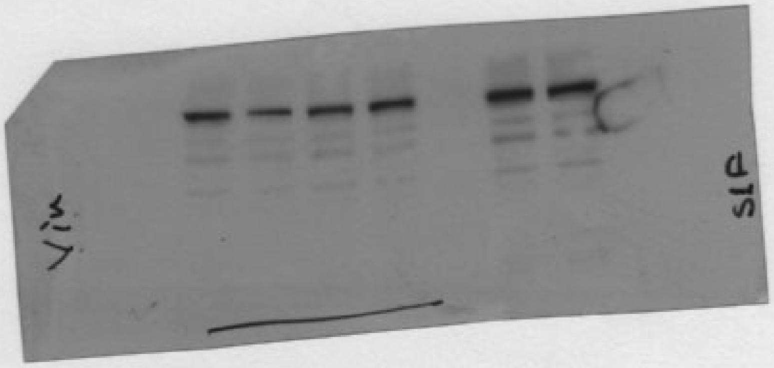
Vim
S1F

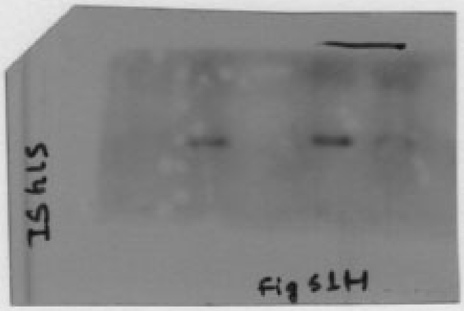
Fig S1H

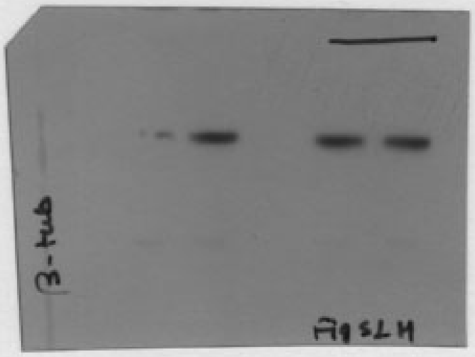
Fig S1H

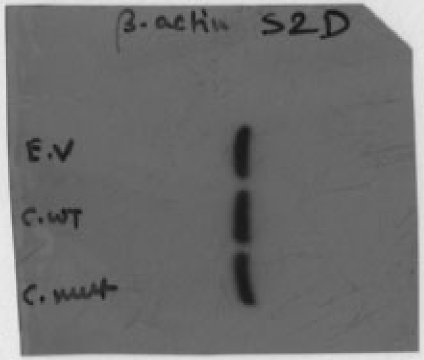
β-actin S2D
E.V
C.WT
C.mut

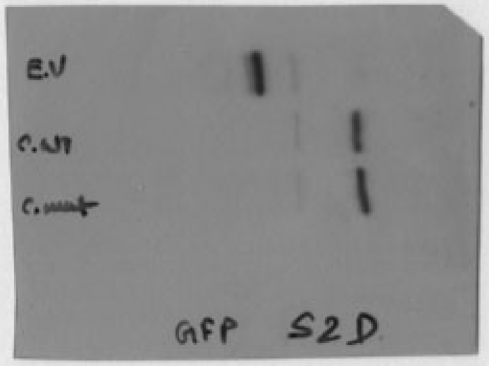
E.V
C.WT
C.mut
GFP S2D

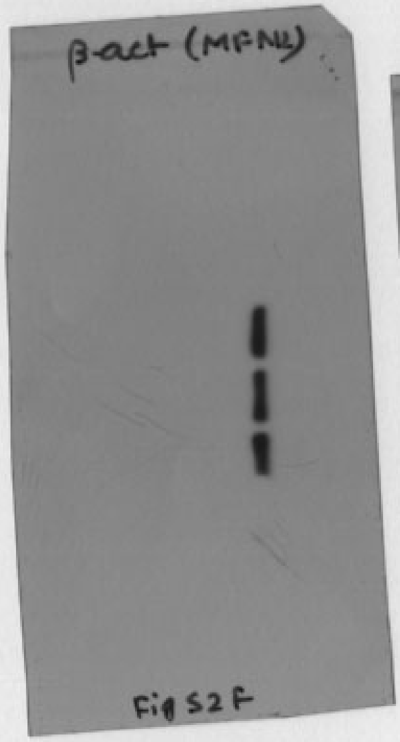
β-act (MFN2)
Fig S2F

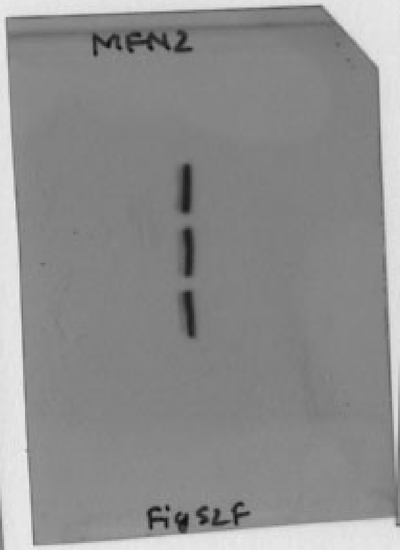
MFN2
Fig S2F

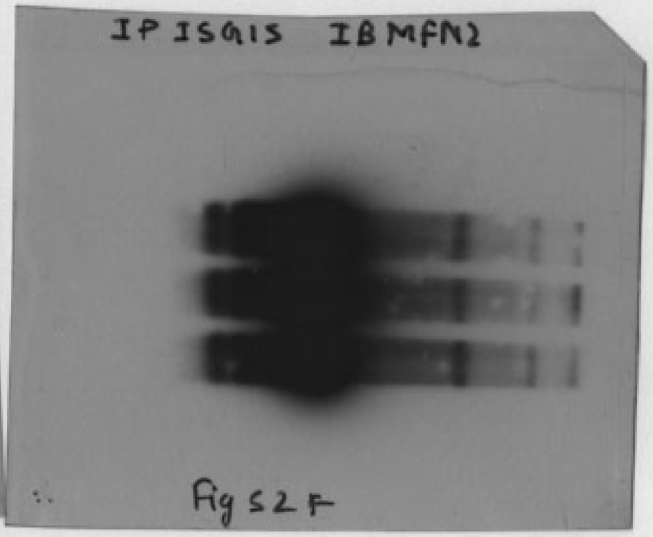
IP ISG15 IB MFN2
Fig S2F

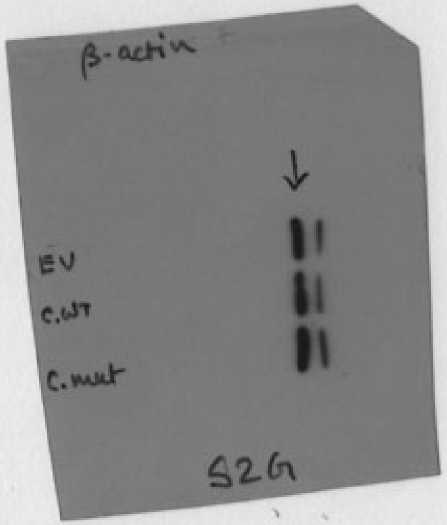
β-actin
EV
C.WT
C.mut
S2G

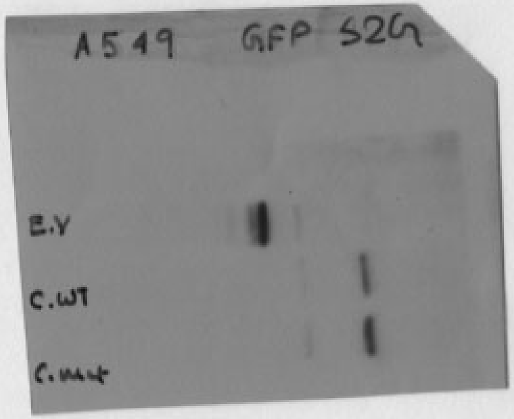
A549 GFP S2G
E.V
C.WT
C.mut

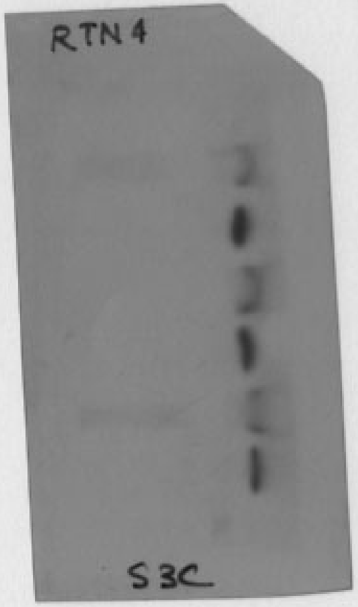
RTN4
S3C
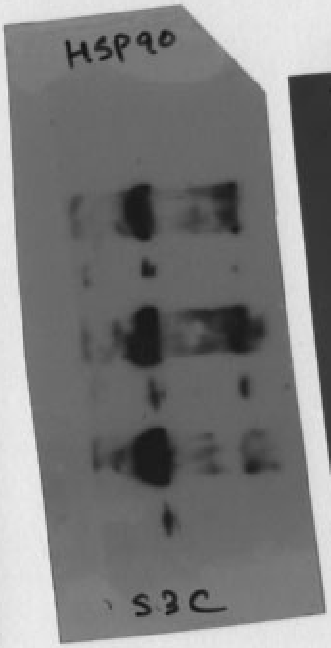
HSP90
S3C
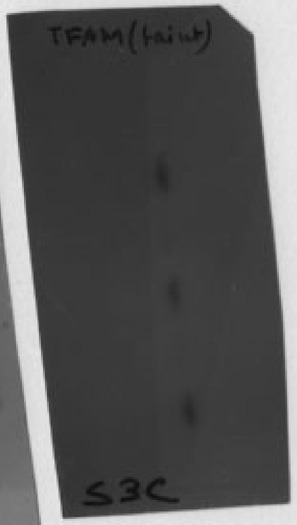
TFAM (faint)
S3C
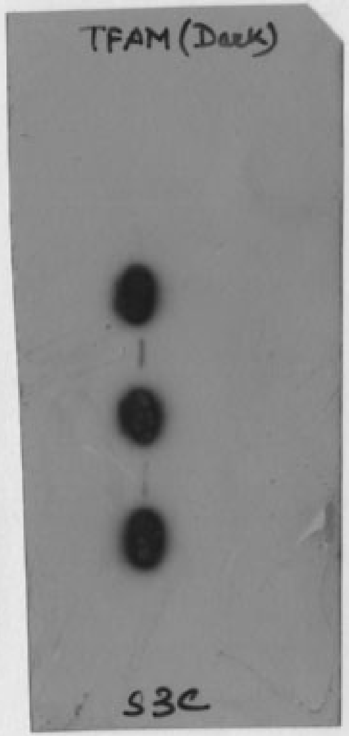
TFAM (Dark)
S3C

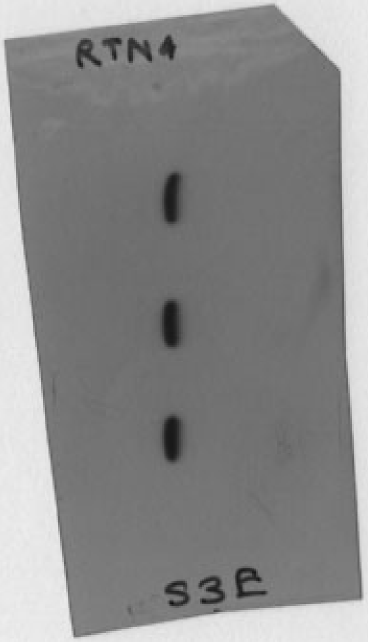
RTN4
S3E
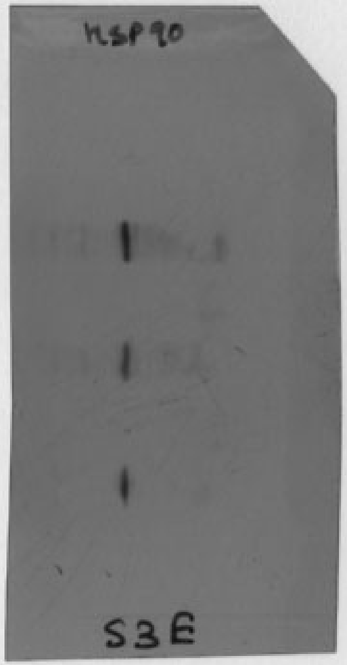
HSP90
S3E
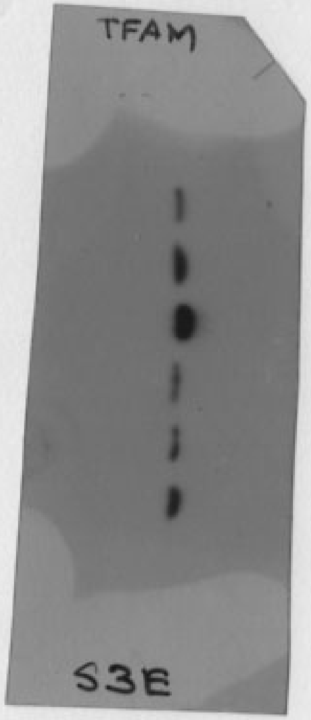
TFAM
S3E

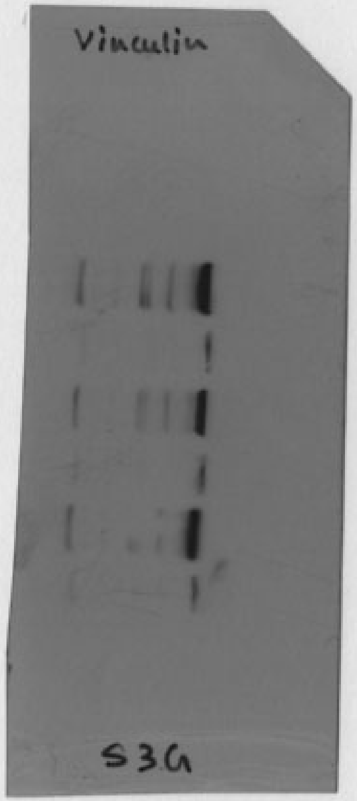
Vinculin
S3G

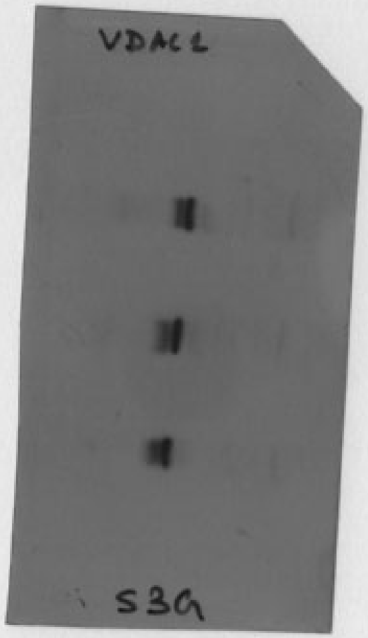
VDAC1
S3G

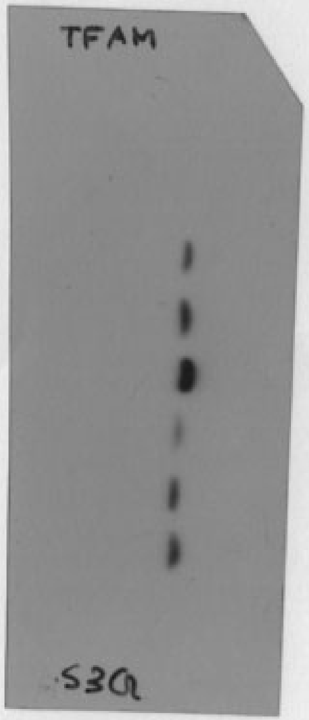
TFAM
S3G

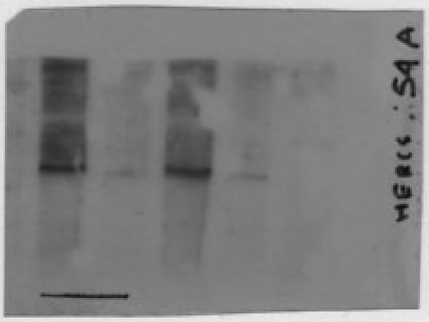
HERCS :: S4A

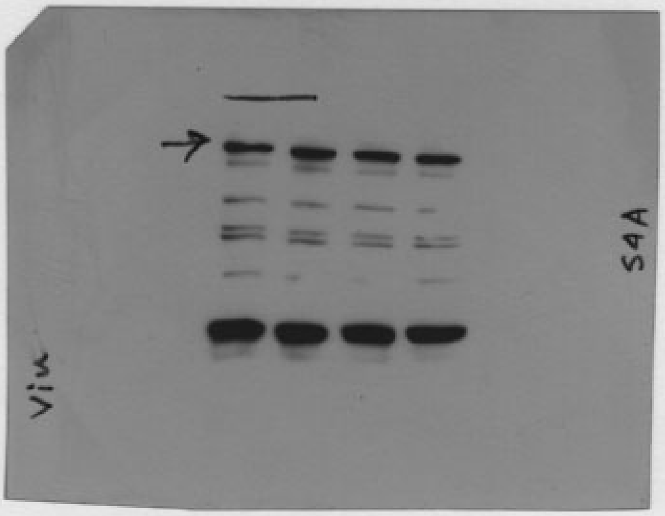
S4A
Vin

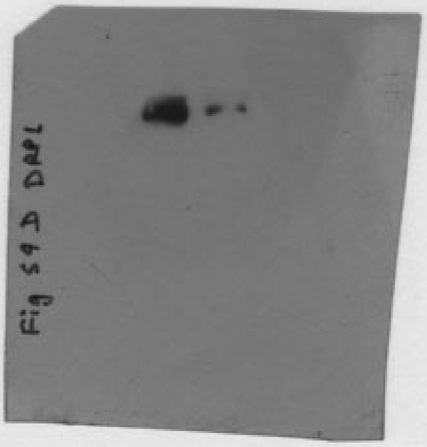
Fig S4D DRP1

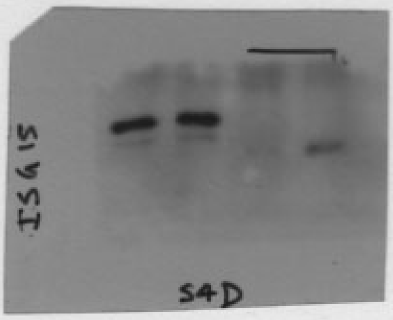
ISG15
S4D

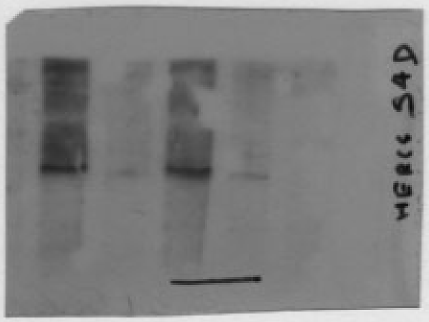
S4D

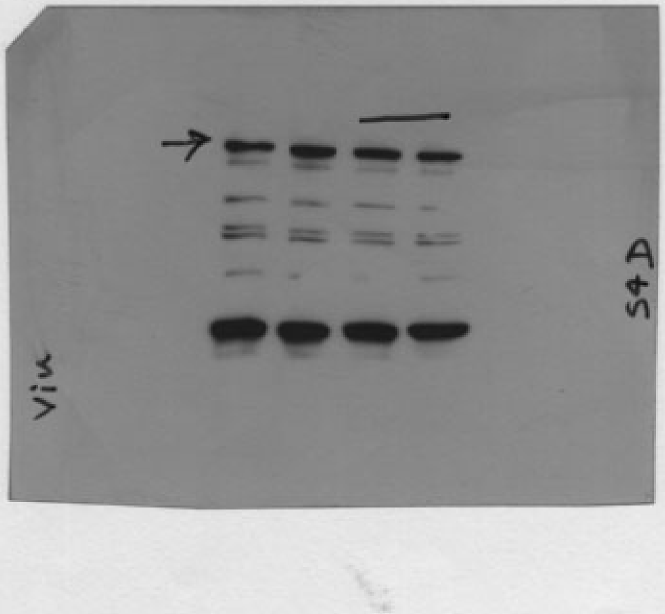
Vin
S4D

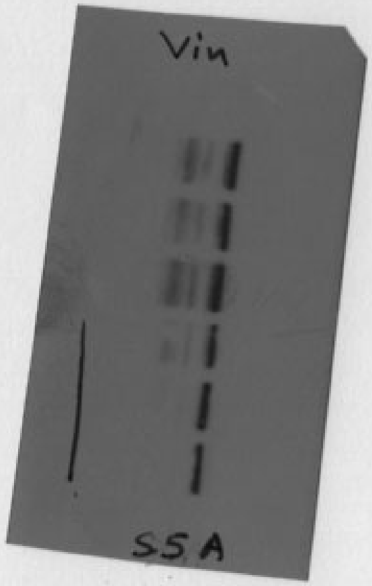
Vin
S5A

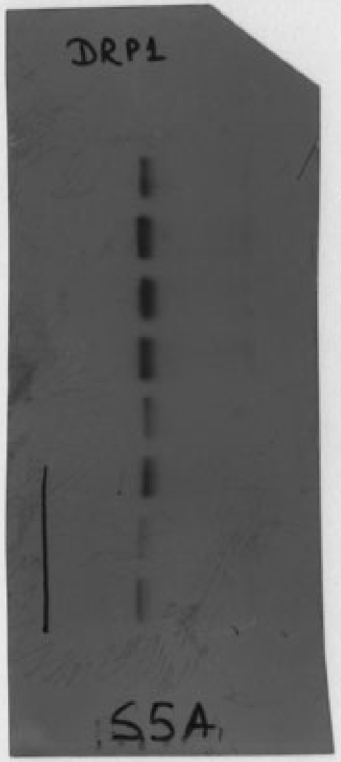
DRP1
S5A

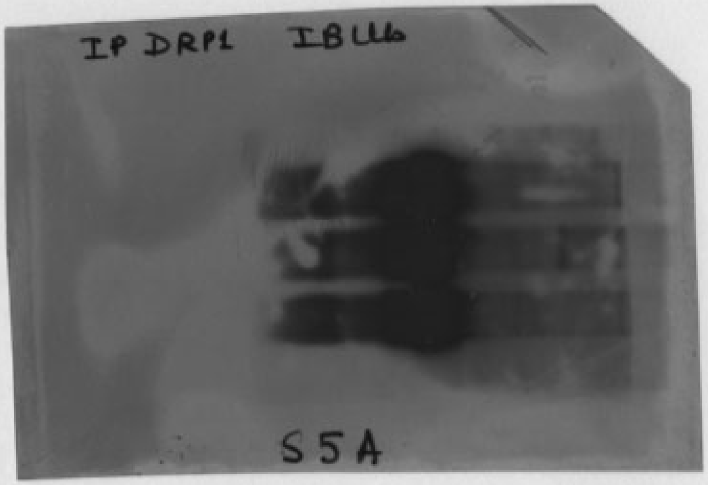
IP DRP1 IB Ub
S5A

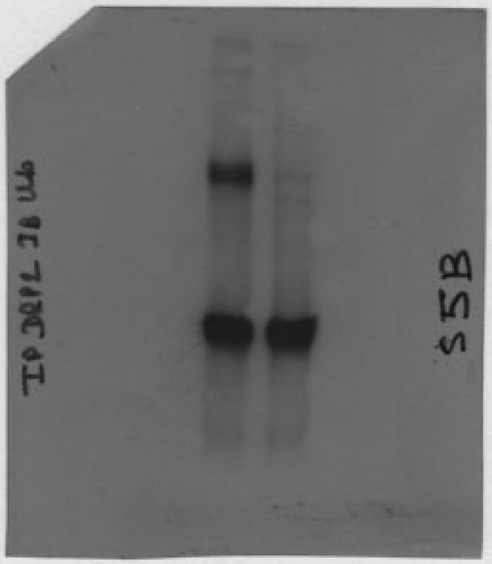
IP DRP1 IB Ub
S5B

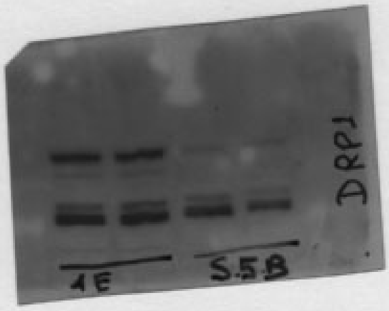
DRP1
1E
S.5.B

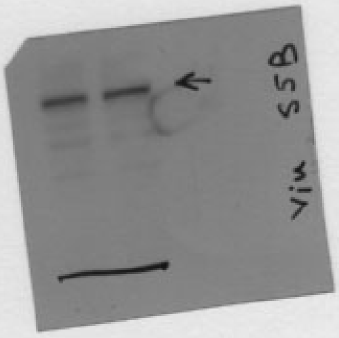
Vim S5B

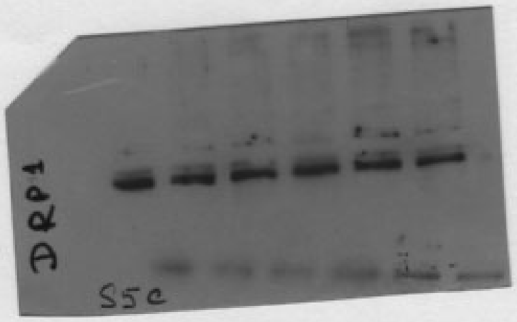
DRP1
S5e

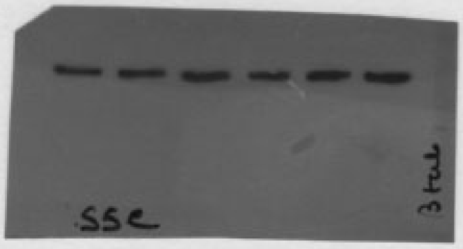
S5e

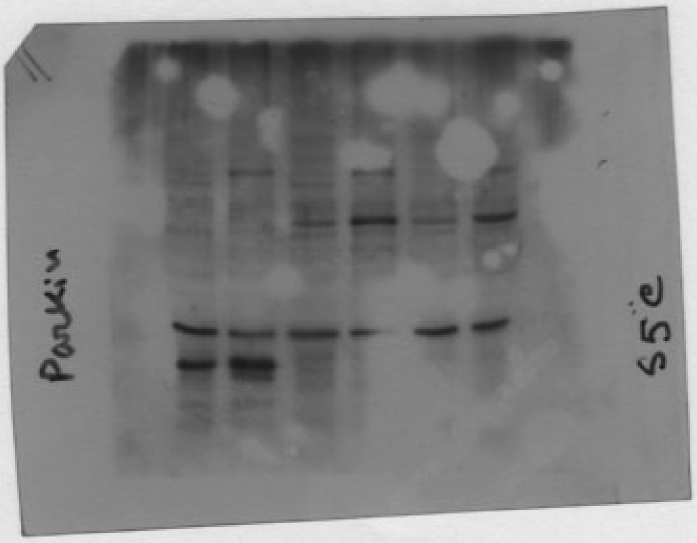
Parkin
S5e

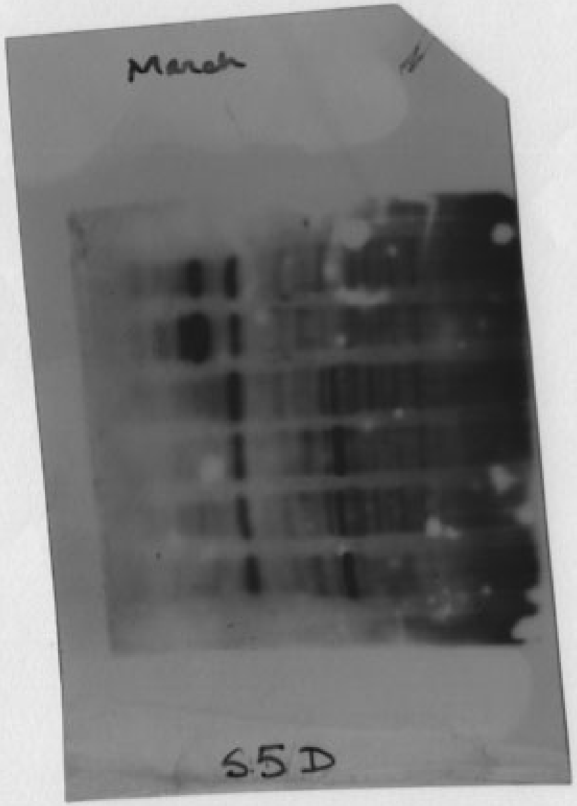
March
S5D

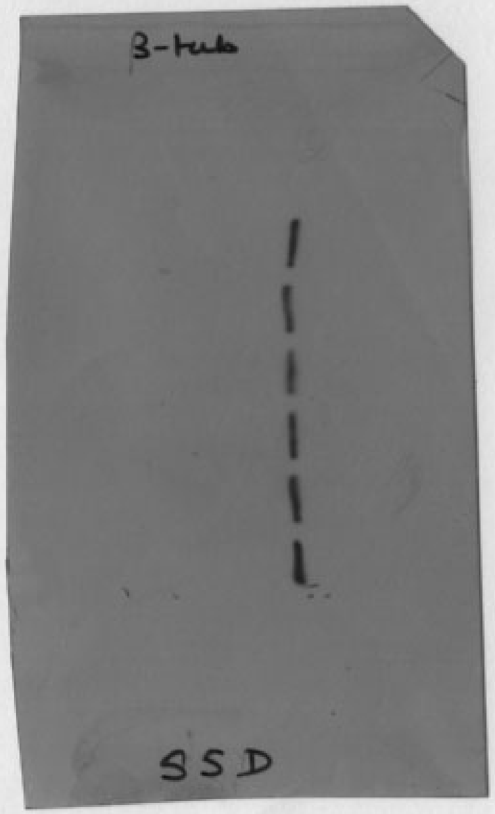
β-tub
S5D

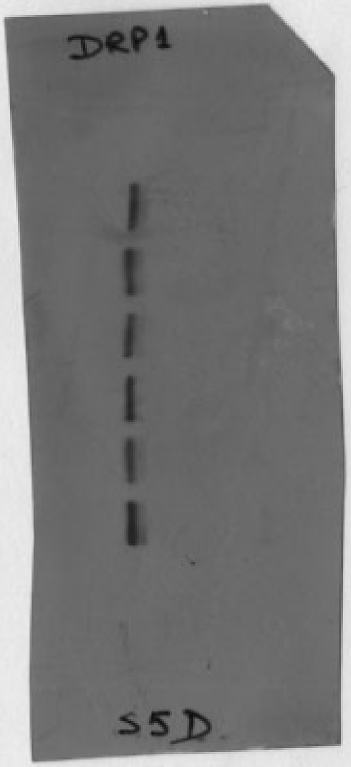
DRP1
S5D

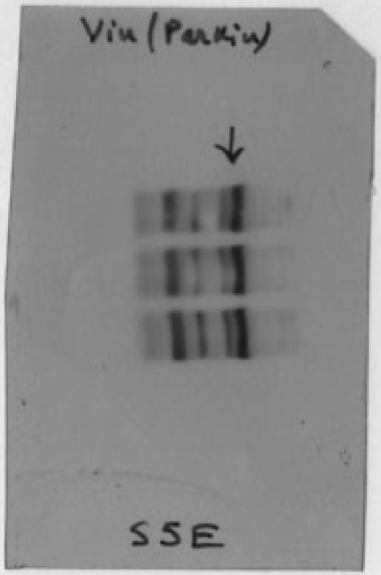
Vin (Parkin)
S5E

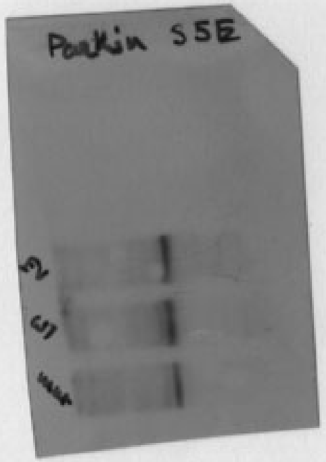
Parkin S5E
EV
WT

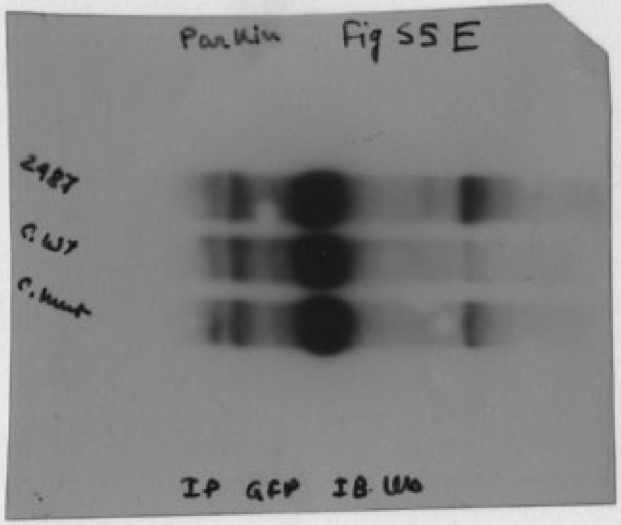
Parkin Fig S5E
2487
C.WT
C.mut
IP GFP IB Ub

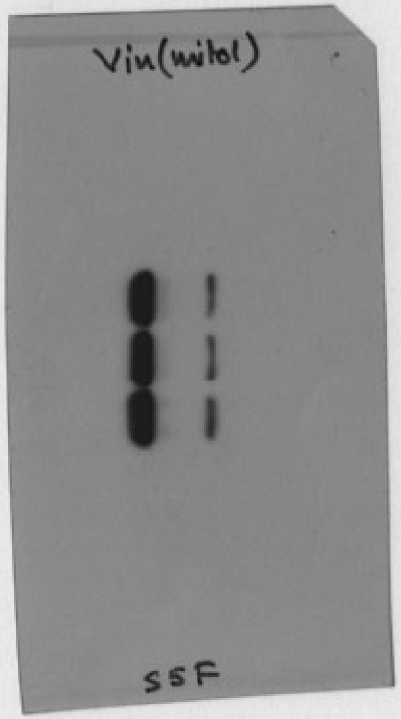
Vin(mitol)
SSF

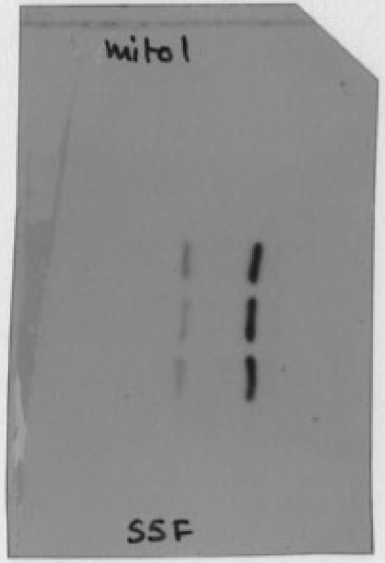
mitol
SSF

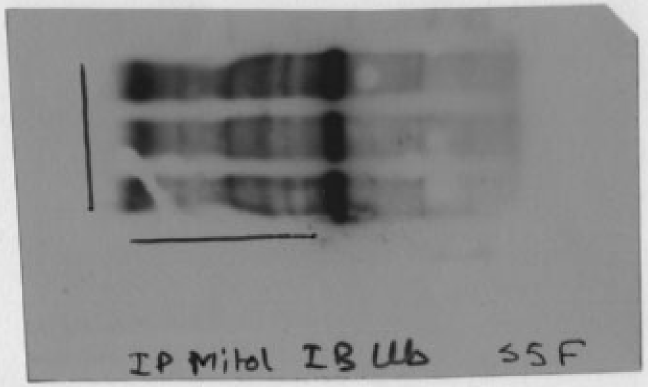
IP Mitol IB Ub
SSF

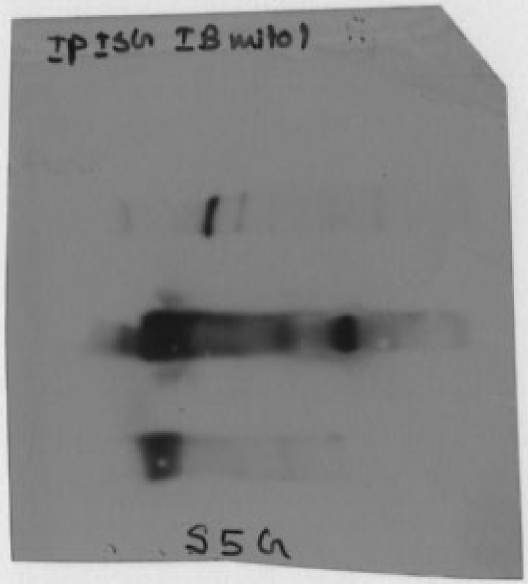
IP ISG IB mito1
S5G

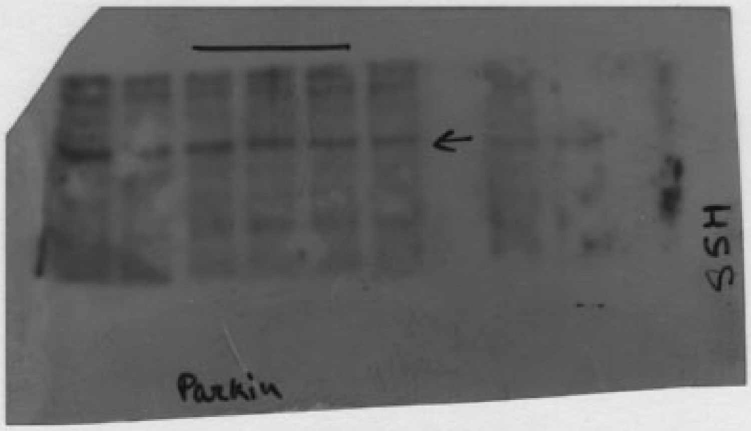
SSH
Parkin

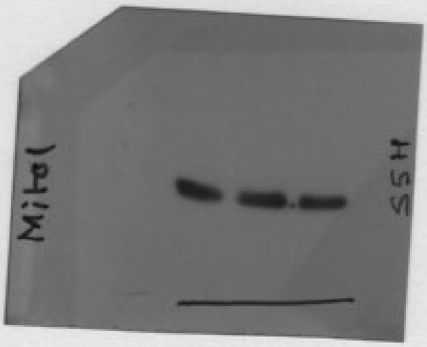
Mitol
SSH

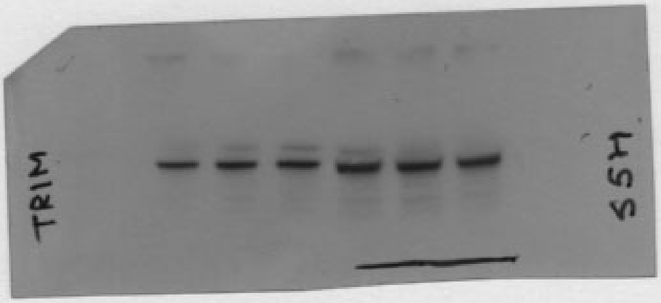
TRIM
SSH

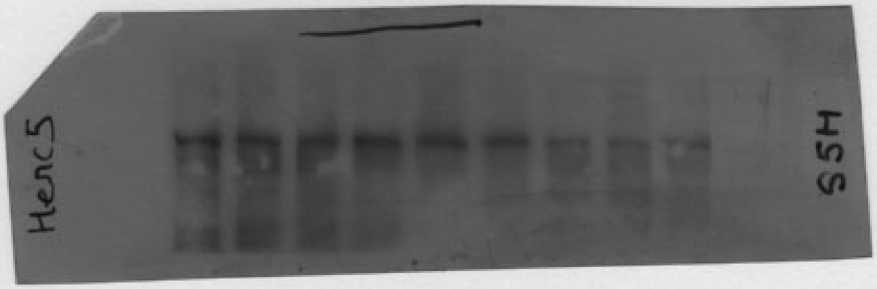
SSH

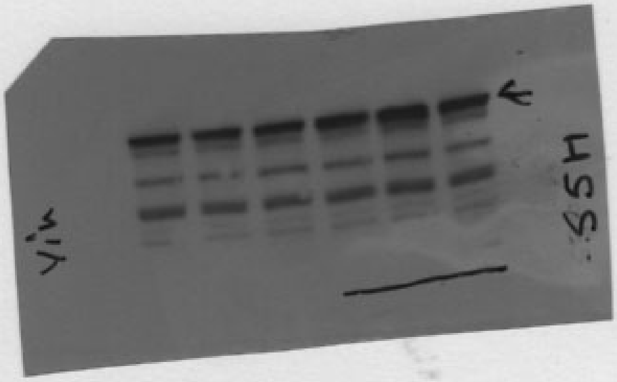
SSH

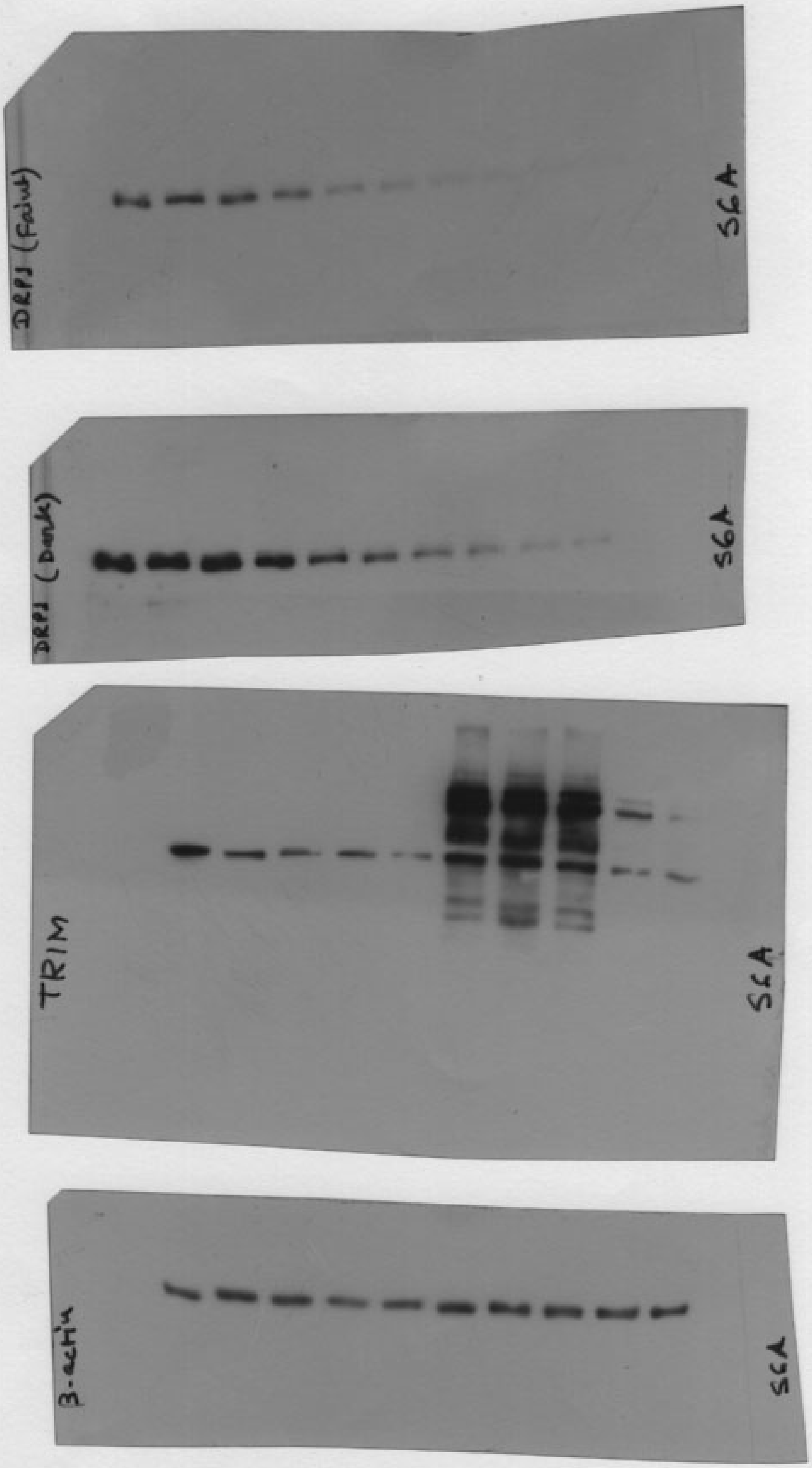

DRP1 (Faint)
SGA
DRP1 (Dark)
SGA
TRIM
SGA
β-actin
SGA

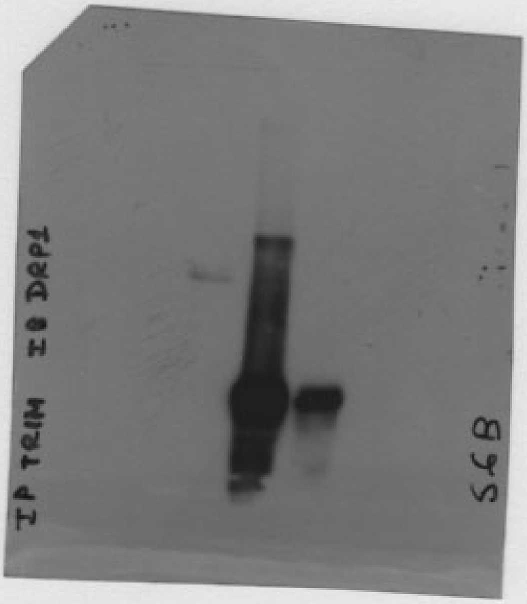
IP TRIM IB DRP1

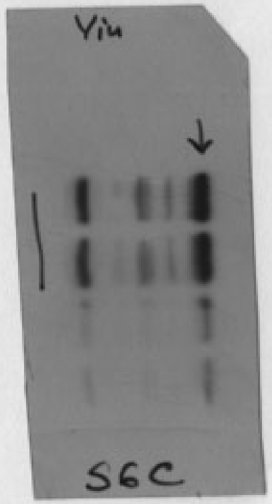
Vin
S6C
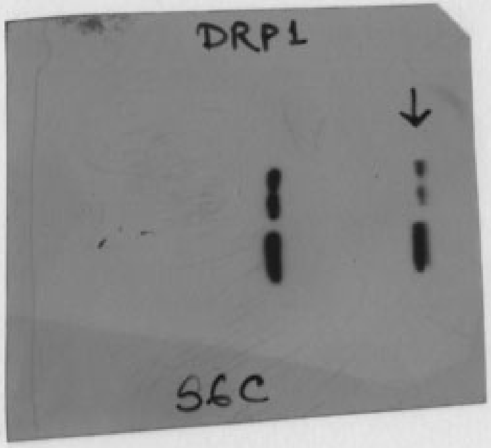
DRP1
S6C
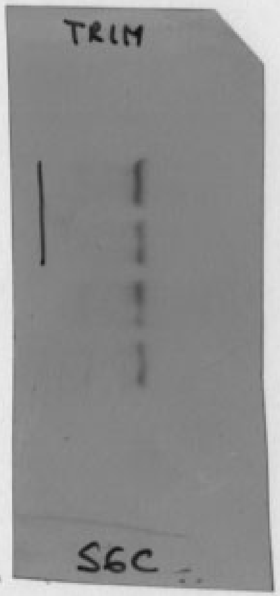
TRIM
S6C
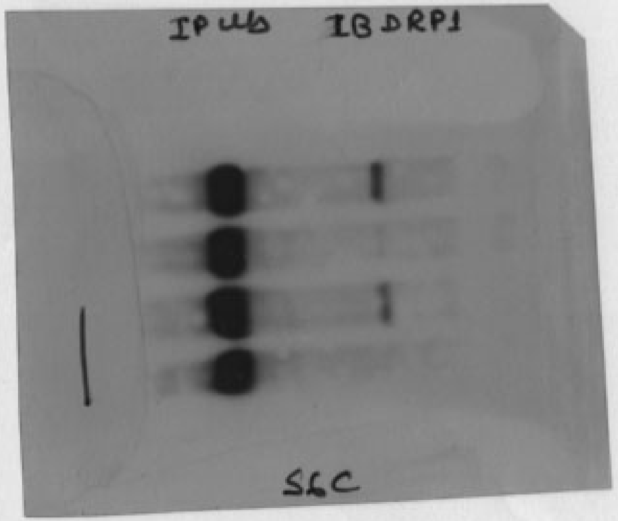
IP ub IB DRP1
S6C

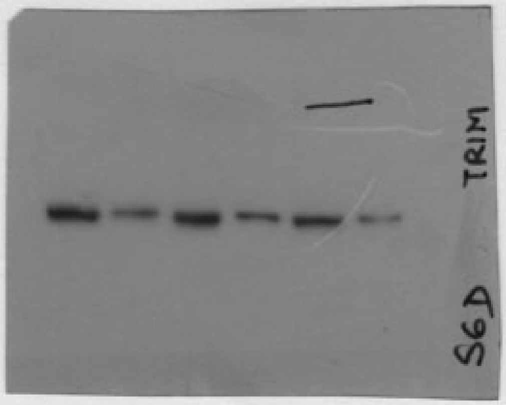
TRIM
S6D

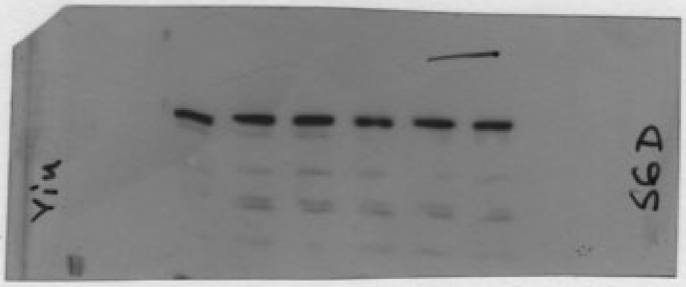
Yin
S6D

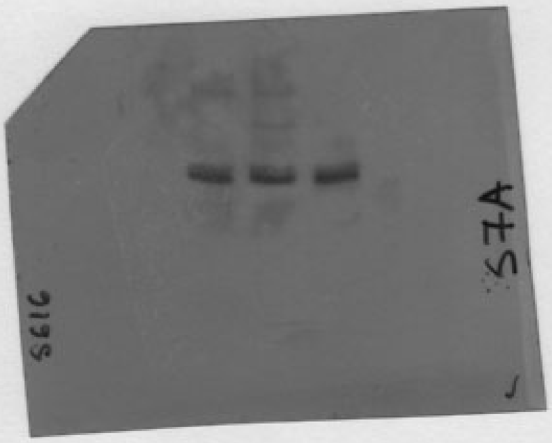
S616
S7A

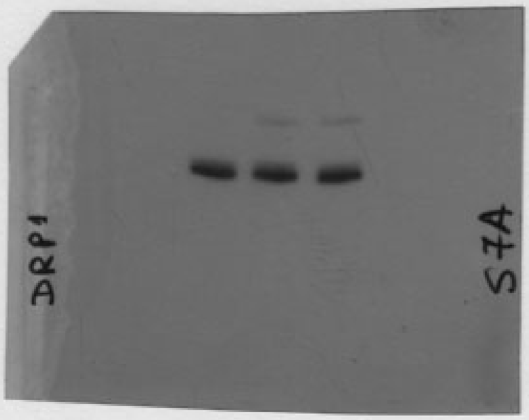
DRP1
S7A

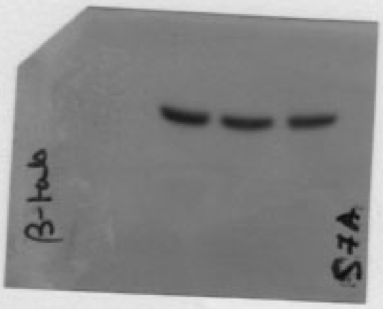
β-tub
S7A

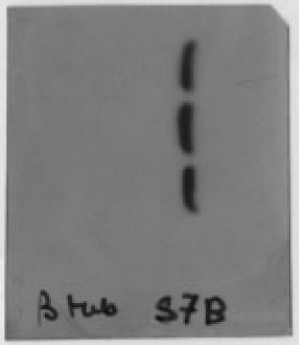
β tub S7B
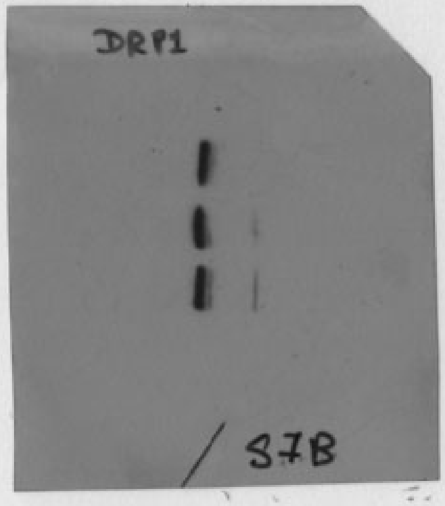
DRP1
S7B
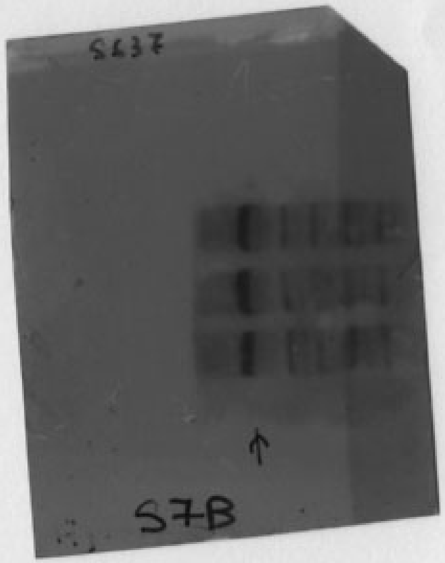
S7B

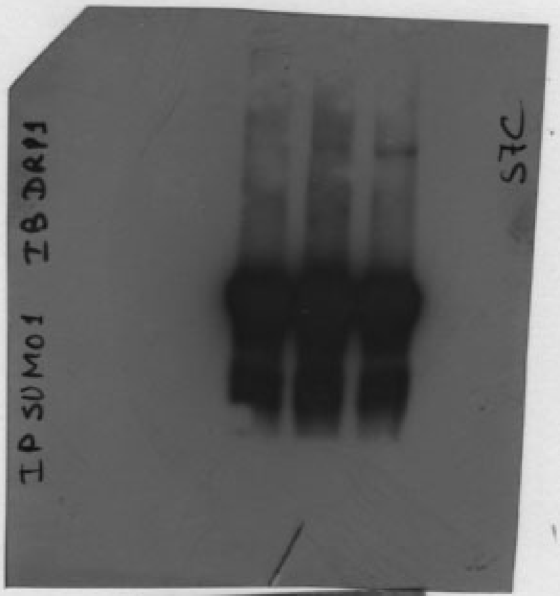
IP SUMO1 IB DRP1
S7C
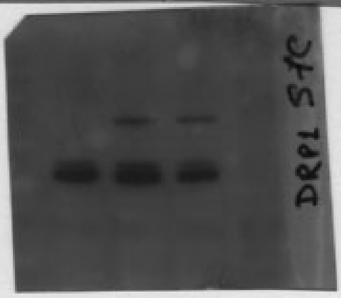
DRP1 S7C
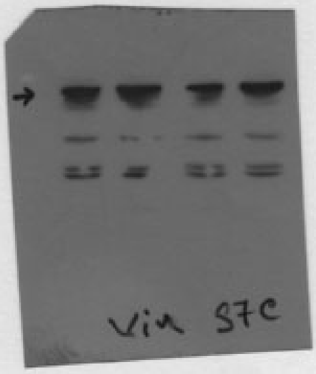
Vin S7C

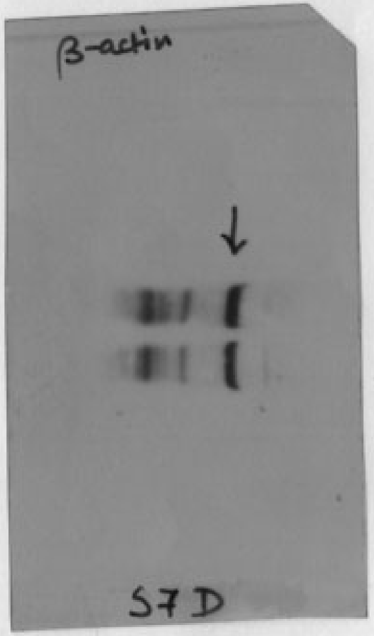
β-actin
S7D
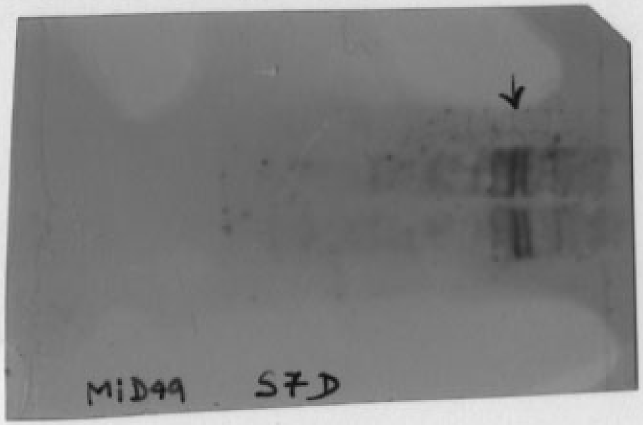
MiD49
S7D
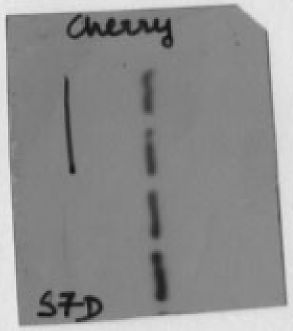
Cherry
S7D
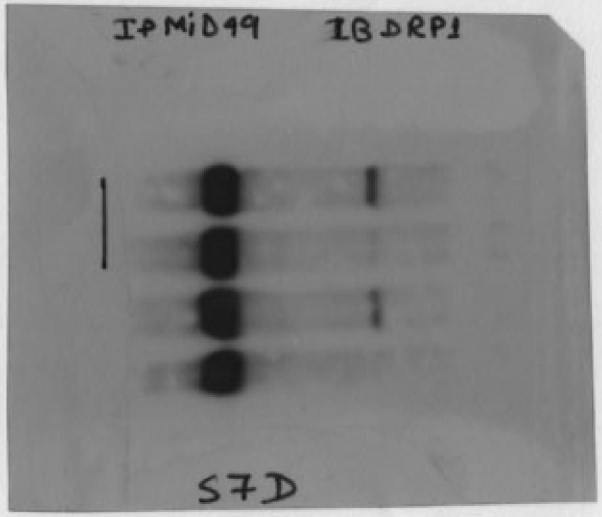
IP MiD49
IB DRP1
S7D

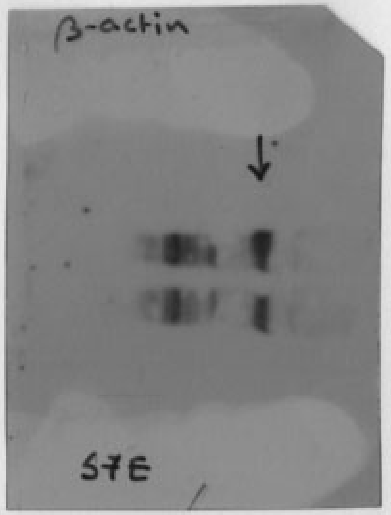
β-actin
S7E

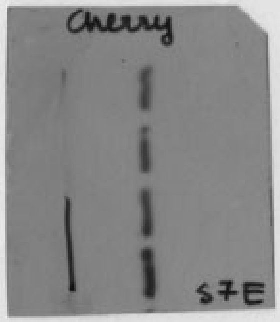
Cherry
S7E

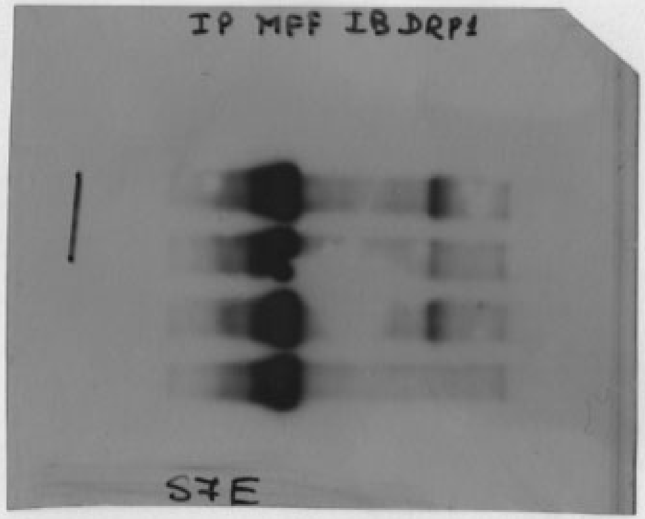
IP MFF IB DRP1
S7E

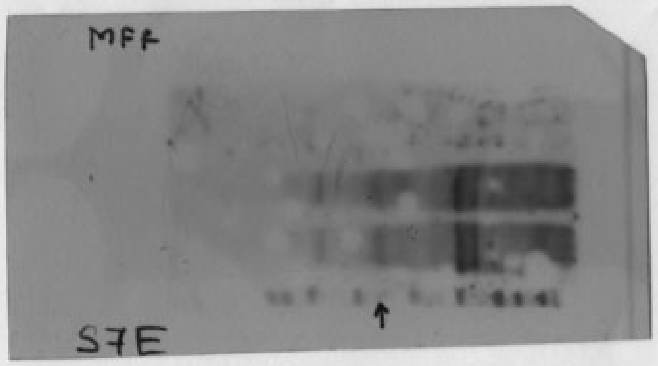
MFF
S7E

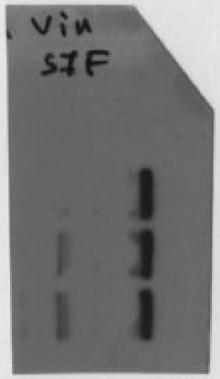
Vin
S7F

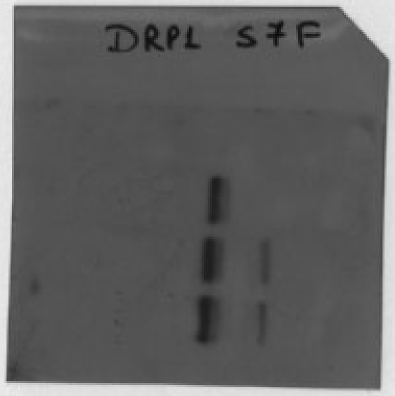
DRPL S7F

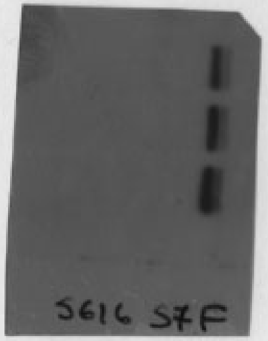
S616 S7F

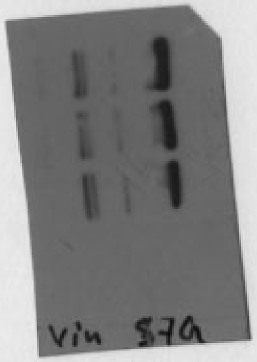
Vin S7G

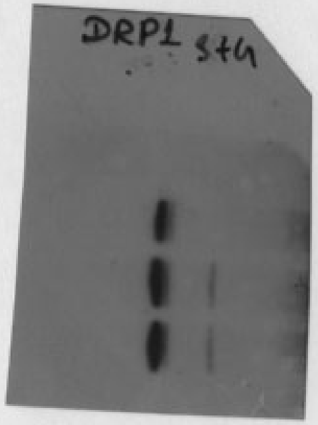
DRP1 S7G

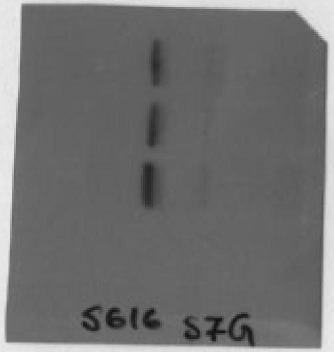
S616 S7G
